# Analysis of Pharmacological Activities and Mechanisms of Essential Oil in Leaves of *C. grandis* ‘Tomentosa’ by GC-MS/MS and Network Pharmacology

**DOI:** 10.2174/1386207325666220610182644

**Published:** 2023-04-27

**Authors:** Jie-Shu You, Sheng-Cai He, Liang Chen, Zhen-Hui Guo, Fei Gao, Min-Yue Zhang, Liu Dan, Wei Chen

**Affiliations:** 1 School of Basic Medical Sciences, Guangzhou University of Chinese Medicine, Guangzhou, Guangdong Province, China;; 2 College of Pharmacy, Shenzhen Technology University, Shenzhen, Guangdong Province, China;; 3 School of Physical Education and Health, Guangzhou University of Chinese Medicine, Guangzhou, Guangdong Province, China;; 4 College of Pharmacy, Chengdu University of Traditional Chinese Medicine, Chengdu, Sichuan Province, China;; 5 Division of Hematology, Renji Hospital, School of Medicine, Shanghai Jiaotong University, Shanghai, China;; 6 Galactophore Department, Guangdong Provincial Hospital of Chinese Medicine, Guangzhou, Guangdong Province, China

**Keywords:** Leaves of *C. grandis* ‘Tomentosa,’ essential oils, GC-MS/MS, network pharmacology, component-target-disease, tomentosa

## Abstract

**Background:**

*Citrus grandis* ‘Tomentosa,’ a fruit epicarp of *C. grandis* ‘Tomentosa’ or *C. grandis* (L.) Osbeck is widely used in health food and medicine. Based on our survey results, there are also rich essential oils with bioactivities in leaves, but the chemical compounds in this part and relevant pharmacological activities have never been studied systematically. Therefore, this study was to preliminarily decipher the pharmacological activities and mechanisms of the essential oil in leaves of *C. grandis* ‘Tomentosa’ by an integrated network pharmacology approach.

**Methods:**

Essential oil compositions from leaves of *C. grandis* ‘Tomentosa’ were identified using GC-MS/MS. And then, the targets of these oil compositions were predicted and screened from TCMSP, SwissTargetPrediction, STITCH and SEA databases. STRING database was used to construct the protein-protein interaction networks, and the eligible protein targets were input into WebGestalt 2019 to carry out GO enrichment and KEGG pathway enrichment analysis. Based on the potential targets, disease enrichment information was obtained by TTD databases. Cytoscape software was used to construct the component-target-disease network diagrams.

**Results:**

Finally, 61 essential oil chemical components were identified by GC-MS/MS, which correspond to 679 potential targets. Biological function analysis showed 12, 19, and 12 GO entries related to biological processes, cell components and molecular functions, respectively. 43 KEGG pathways were identified, of which the most significant categories were terpenoid backbone biosynthesis, TNF signaling pathway and leishmaniasis. The component-target-disease network diagram revealed that the essential oil compositions in leaves of *C. grandis* ‘Tomentosa’ could treat tumors, immune diseases, neurodegenerative diseases and respiratory diseases, which were highly related to CHRM1, PTGS2, CASP3, MAP2K1 and CDC25B.

**Conclusion:**

This study may provide new insight into *C. grandis* ‘Tomentosa’ or *C. grandis* (L.) Osbeck and may provide useful information for future utilization and development.

## 1 INTRODUCTION

*Citri Grandis* Exocarpium (Huajuhong), recorded officially in the current Chinese Pharmacopoeia (2020 edition), is the fruit epicarp of *C. grandis* ‘Tomentosa’ or *C. grandis* (L.) Osbeck particularly originated from Huazhou town in Guangdong province, southern China. *Citri Grandis* Exocarpium is a medicinal and edible food known for its nutritional benefits and pharmaceutical effects. It is a rich source of flavonoids, essential oil, polysaccharides, coumarins and limonoids [1-3]. Its therapeutic effects in traditional Chinese medicine include regulating qi-flowing and eliminating dampness and phlegm. Modern research further demonstrated that *Citri grandis* Exocarpium has anti-tussive, anti-oxidant, anti-inflammatory, anti-microbial, anti-proliferative and anti-atherosclerotic activities [4, 5].

Fruit epicarp and whole fruits are the most commonly used parts of *C. grandis* ‘Tomentosa’ or *C. grandis* (L.) Osbeck, in which essential oils play an important role in pharmacological effects. Essential oil is a concentrated hydrophobic liquid containing volatile aroma compounds usually extracted from plants. Essential oils are often used for aromatherapy to induce relaxation, and proper application can effectively treat diseases [6]. Based on our survey results, there is also rich essential oil in the leaves of *C. grandis* ‘Tomentosa,’ but the chemical compounds in this part and relevant pharmacological activities have never been studied systematically. Due to the limited availability of reference substances, gas chromatography coupled with tandem mass spectrometry (GC-MS/MS) was applied to characterize components in this study, which have been widely accepted to be the predominant tool for the analysis of essential oil contents. It provides significant advantages for unequivocal identification and quantification of very low limits of ingredients [7]. Network pharmacology, proposed by Andrew L Hopkins [8], can build a “compound-target-disease” multilevel network to analyze the active ingredients, relevant pharmacological activities and possible molecular network mechanisms, which is in accordance with the connotation of holistic theory, multi-components and multi-targets of Chinese medicine [9-11].

In this study, essential oil contents in the leaves of *C. grandis* ‘Tomentosa’ were identified by gas chromatography coupled with tandem mass spectrometry (GC-MS/MS) and network pharmacology established the compound-target-disease network to explore the potential pharmacological activities and mechanism. The results will provide direct and reliable evidence for the broader research and application of *C. grandis* ‘Tomentosa,’ which will reduce resource waste and bring economic benefits.

## 2 MATERIALS AND METHODS

### 2.1 Identification of Essential Oil Composition

**Fig. (1):**
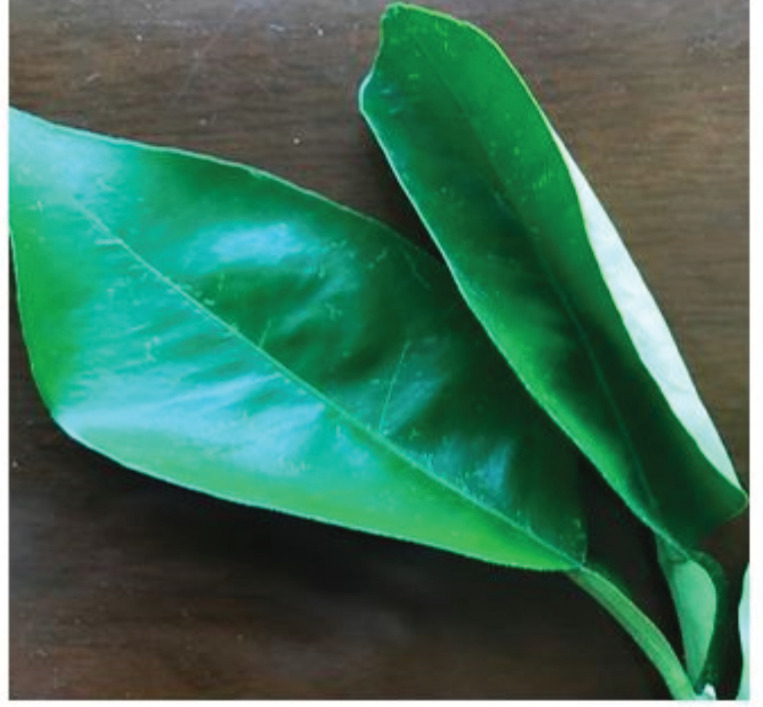
Leaves of *C. grandis* ‘Tomentosa’.

The leaves of *C. grandis* ‘Tomentosa’ (Fig. **1**) were obtained from the genuine producing area of Huazhou city and authenticated by Professor Huan-lan Liu from Guangdong University of Chinese Medicine. 50 g leaf pieces were extracted by Soxhlet extractor with 150 mL anhydrous ether for 18 h. Then the extract was dried with anhydrous sodium sulfate and concentrated to dryness with a termovap sample concentrator. The ether was evaporated, and the volume was adjusted to 1 mL with n-hexane. The sample was filtered through a 0.22 μm PTFE syringe filter. GC-MS/MS determined the chemical composition of essential oil. Moreover, GC analysis was performed on an Agilent 5977B GC-MS/MS system equipped with an Agilent Multimode injector. The column used was an HP-5ms, 30 m × 0.25 mm i.d., 0.25μm film thickness. The carrier gas was helium at a constant flow rate of 1.0 mL/min. The injection was conducted in splitless mode at 250°C for 3 min, and the injected volume was 1 µL. The oven temperature program consisted of the following steps: an initial temperature of 60°C maintained for 3 min, heating from 60°C to 110°C at a rate of 5°C/min, and then raised to 150°C at a rate of 4°C/min, where the final temperature of 240°C was held for 5 min. The temperature of the transfer line was 240°C. The mass spectrometer was operated in electron ionization mode at 70 eV, and a triple quadrupole mass spectrometer detected the ions. Data acquisition and analyses were performed using the MassHunter Workstation software.

### 2.2 Targets Screening of Essential Oil Composition

The protein targets of the essential oil composition in leaves of *C. grandis* ‘Tomentosa’ were searched *via* the Traditional Chinese Medicine Systems Pharmacology Database and Analysis Platform (TCMSP, http://tcmspw.com/tcmsp.php), SwissTargetPrediction (http://www.swisstargetpre diction.ch/), STITCH (http://stitch.embl.de/), and Similarity ensemble approach [12] (SEA, http://sea.bkslab.org/) for each chemical component. *Homo sapiens* were the only species for the targets, and the repetitive targets collected were removed. Then the component-target network of essential oil composition was constructed using Cytoscape software (Version 3.2.1) [13]. The network was analyzed using the Cytoscape plugin CentiScaPe to calculate topological parameters, including the degree, betweenness centrality, closeness centrality and average shortest path length [14]. A significant node represented the major ingredients and targets, and edges encoded the interactions.

### 2.3 Construction and Analysis of Protein-Protein Interaction (PPI)

The PPI analysis was constructed by the open Search Tool for the Retrieval of Interacting Genes (STRING) database (https://string-db.org/cgi/input.pl), which contains information on protein/gene interactions, including verified experimental data, computationally predicted data, and public text collections [15]. The targets of the essential oil composition were imported into the STRING database, and the species was defined as *Homo sapiens*. Then only the data on PPIs with high confidence scores (scores ≥ 0.9) were adopted for further analysis.

### 2.4 Gene Ontology (GO) Functional and Pathway Enrichment Analyses

From a systematic point of view, the interaction of target proteins is more likely to participate in different biological processes and other cellular components under the cells instead of performing their functions independently [16]. In this study, the targets obtained from PPI network analysis were input into the WEB-based Gene SeT AnaLysis Toolkit (WebGestalt, www.webgestalt.org) 2019 to carry out GO enrichment and Kyoto Encyclopedia of Genes and Genomes (KEGG) pathway enrichment analysis. WebGestalt [17] supports 12 organisms, 342 gene identifiers and 155175 functional categories and is widely used for gene set enrichment analysis. The GO and KEGG pathway enrichment analyses were carried out for the top 10 hub genes with a threshold of FDR < 0.05.

### 2.5 Network Construction of Active Component-Target-Disease

Diseases were obtained from the Therapeutic Target Database (TTD, http://db.idrblab.net/ttd/) [18] based on the successful clinical trial targets *via* PPI network analysis. Then the component-target-disease network was constructed using Cytoscape software (Version 3.2.1).

## 3 RESULTS

### 3.1 Determination of Essential Oil Composition in Leaves of *C. grandis* ‘Tomentosa’

**Fig. (2):**
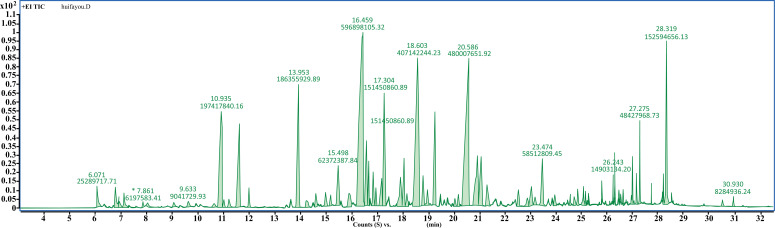
A total ion chromatogram of essential oil from leaves of *C. grandis* ‘Tomentosa’.

A total ion chromatogram obtained by GC-MS/MS for essential oil composition in leaves of *C. grandis* ‘Tomentosa’ was shown in (Fig. **2**). In total, 61 essential oil chemical components were identified and listed in (Table **1**). The families of detected essential oils in leaves mainly included terpenes (48.41%) and the oxygen-containing derivatives of the terpenes-alcohols (34.73), which were consistent with the previous reports, but the contents were higher than those extracted from the *Citri grandis* Exocarpium (fruit) [19, 20]. The major compound was β-caryophyllene (15.75%), followed by (3R,6E)-nerolidol (12.66%), bicyclogermacrene (10.74%), β-citronellol (5.21%), 1-Methyl-4-(1-methyl ethylidene)-2-(1-methylvinyl)-1-vinylcyclohexane (4.92%), geraniol (4.12%) and phytol (4.03%).

### 3.2 Potential Targets of Essential Oil Composition

**Fig. (3):**
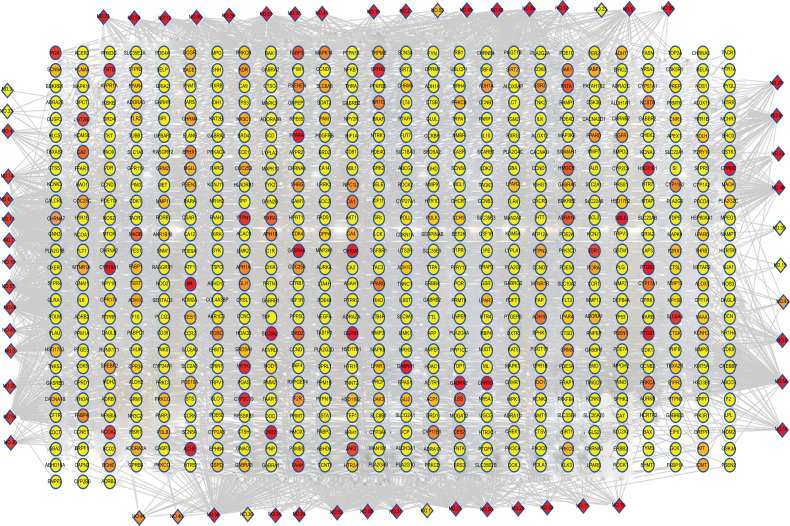
The component-target network of essential oil composition. The diamond nodes represent ingredients, and the circular nodes represent targets. The colors of the nodes are illustrated from red to yellow in descending order of degree values.

A total of 2417 potential targets of all essential oil compositions for *Homo sapiens* were obtained from the TCMSP, SwissTargetPrediction, STITCH and SEA databases after deleting repetitions (Supplementary file, Table **S1**). (Fig. **3**) shows the component-target network of essential oil composition in leaves of *C. grandis* ‘Tomentosa,’ which contained 679 targets. The circular nodes represent the targets of essential oil composition, and the diamond nodes represent the chemical composition of essential oil. Each edge represents the interaction between the active component and the target. Only the targets with higher values of “degree” (above two-fold of the median value), “betweenness centrality” and “closeness centrality” (above the median value), and “average shortest path length” (below the median value) were identified as the candidate targets of the essential oil composition in leaves of *C. grandis* ‘Tomentosa.’ Ultimately, 4 direct targets were found to be highly correlated with the essential oil composition, among which PTGS2, CHRM1, GGPS1 and MAPK14 were associated with 34, 32, 20 and 9 chemical components, respectively (Table **2**).

### 3.3 Construction and Analysis of the PPI Network

**Fig. (4):**
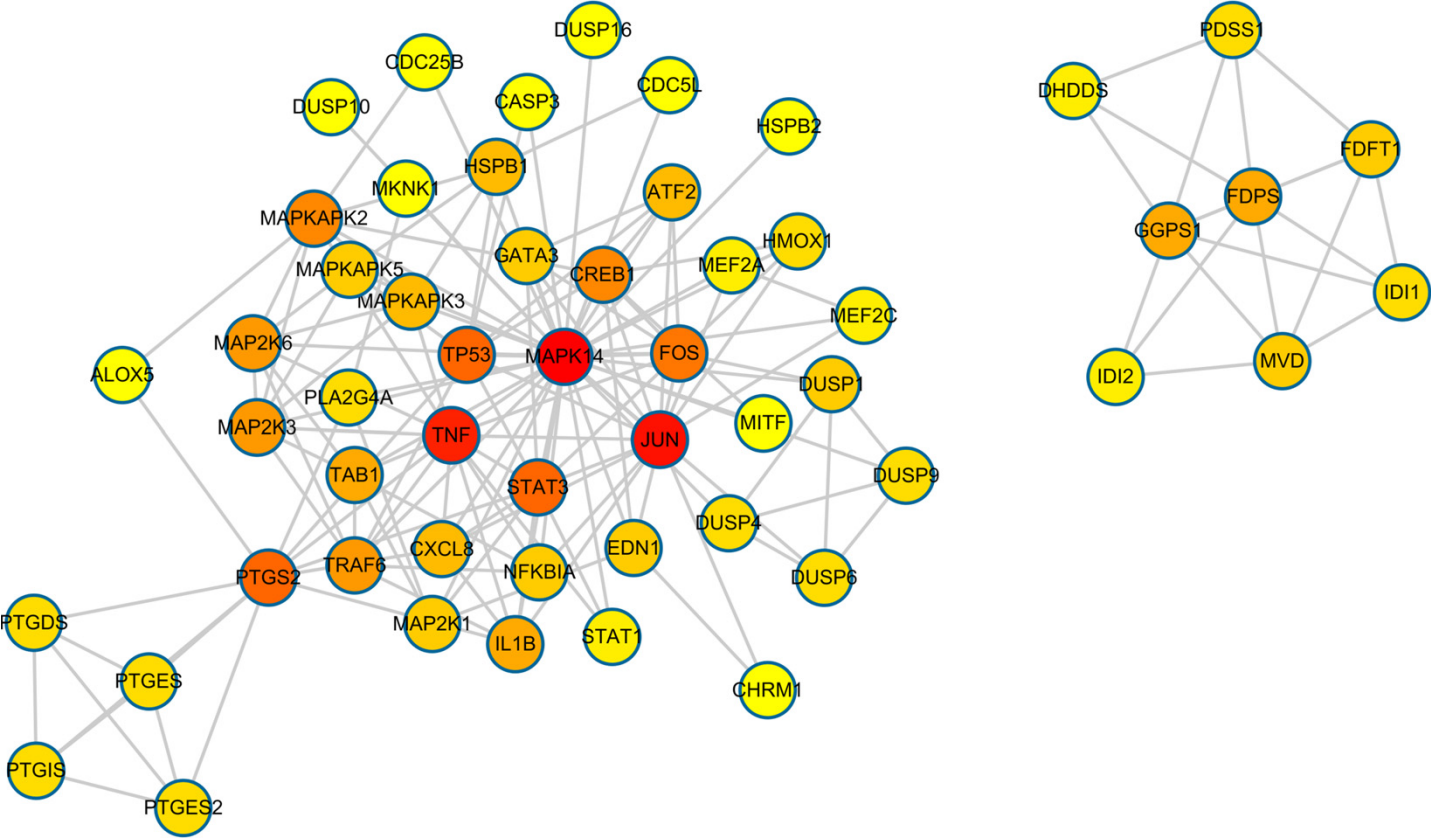
Protein-protein interaction network for essential oil composition. The colors of the nodes are illustrated from red to yellow in descending order of degree values.

The PPI network is shown in (Fig. **4**). The network contained 54 nodes (representing the action target) and 161 edges (representing the association between a pair of action targets). Based on the calculation results from the STRING database, JUN and FOS were found to have the strongest combination ability, and the combined score reached 0.999. According to the PPI network diagram, MAPK14 was in the center of the targets, which could be associated with 38 proteins, followed by JUN and TNF, associating with 16 and 15, respectively.

### 3.4 Functional Enrichment Analysis of Target Protein

**Fig. (5):**
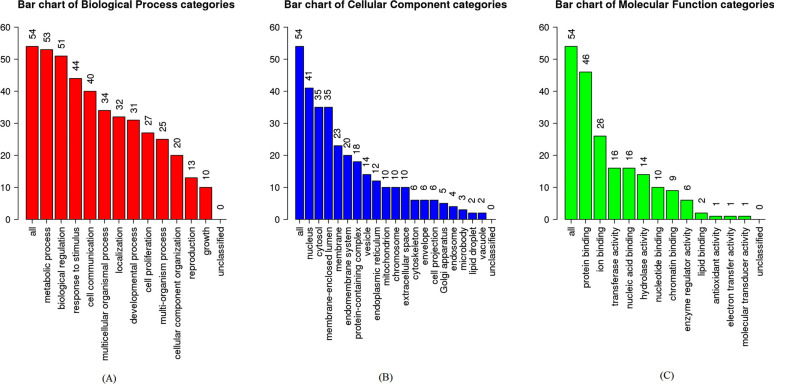
GO enrichment analyses of target proteins. (**A**) biologic process, (**B**) cellular component, (**C**) molecular functions.

**Fig. (6):**
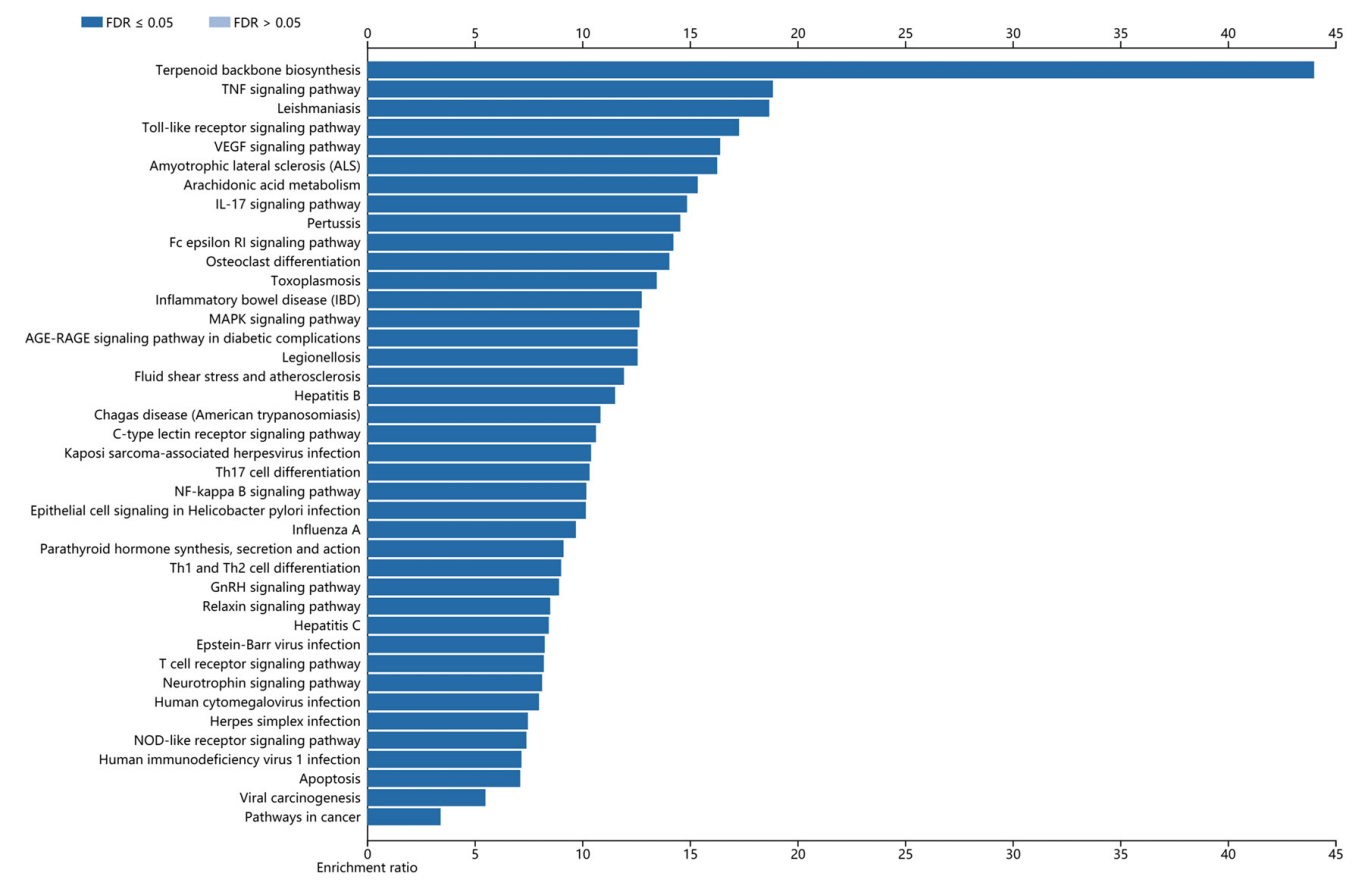
Enriched KEGG pathway of potential targets.

For the biological process, the target proteins were mainly enriched in metabolic process, biological regulation and response to stimulus (Fig. **5A**). In terms of cellular components, it was revealed that these target proteins were mainly enriched in the nucleus, cytosol and membrane-enclosed lumen (Fig. **5B**). For molecular function, it was uncovered that the most target protein was enriched in protein binding, ion binding and transferase activity (Fig. **5C**). The enrichment analysis of the KEGG pathway revealed that 43 enriched categories were identified, of which 40 most significant categories, such as terpenoid backbone biosynthesis, TNF signaling pathway and leishmaniasis, are shown in (Fig. **6**).

### 3.5 Network Construction of Active Component-Target-Disease

Diseases achieved from TTD are shown in (Table **3**). These diseases could be classified as tumors, immune, neurodegenerative, and respiratory diseases. Tumors mainly included oral cancer, solid tumor/cancer, Paget's disease, melanoma, non-small-cell lung cancer, neoplasm and colorectal cancer. Immune diseases include rheumatoid arthritis, inflammation, autoimmune diabetes, immune system disease and arthritis. Neurodegenerative diseases involve Alzheimer’s disease and cognitive impairment. Respiratory diseases mainly included asthma and chronic obstructive pulmonary disease.

**Fig. (7):**
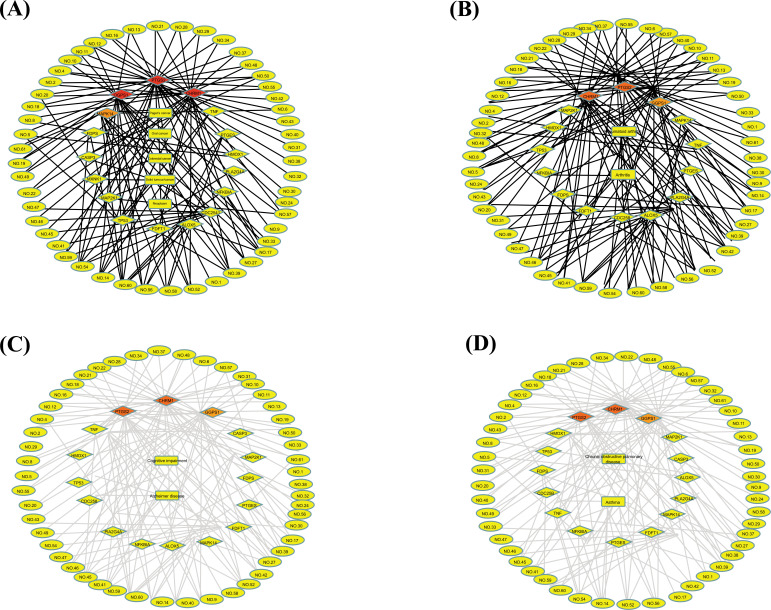
The component-target-disease networks. (**A**) component-target-tumor network, (**B**) component-target-immune disease network, (**C**) component-target-neurodegenerative disease network, (**D**) component-target-respiratory network. The diamond nodes represent ingredients, the circular nodes represent targets, and the rectangle represents diseases. The colors of the nodes are illustrated from red to yellow in descending order of degree values.

Active component-target-tumor network, active component-target-immune disease network, active component-target-neurodegenerative disease network and active component-target-respiratory disease network were established, respectively (Fig. **7**). As shown in (Fig. **7A**), 9 essential oil compositions could act on CASP3, MAP2K1 and CDC25B, respectively and be associated with solid tumor/cancer. These 9 essential oil compositions were farnesol, geranyl linalool, α-Cadinol, T-Muurolol, torreyol, globulol, phytol, methyl palmitate and oleamide. Besides, butyl isodecyl phthalate and methyl linoleate could be linked to neoplasm by acting on PTGES. Tetradecanal, methyl palmitate and oleamide could be linked to Paget's disease by acting on FDPS. Linalool and β-elemene could act on TP53 and be associated with oral cancer and solid tumor/cancer. As shown in (Fig. **7B**), 34 essential oil compositions could act on PTGS2 and be linked to arthritis. The essential oil compositions included phytol, isophytol, farnesol, cadalin, germacrene B, caryophyllene oxide, (-)-γ-cadinene, bicyclogermacrene, (-)-germ acrene D, (1R,4aR,8aS)-7-methyl-4-methylidene-1-propan-2-yl-2,3,4a,5,6,8a-hexahydro-1H-naphthalene, (Z)-caryophy llene, γ-elemene, β-caryophyllene, isocaryophyllene, geranyl acetate, α-copaene, α-cubebene, geraniol, neral, isogeraniol, nerol, β-citronellol, terpinolene, D-limonene, β-elemene, linalool, methyl palmitate, citral, bis(2-ethylhexyl) adipate, oleamide, methyllinolenate, farnesol, (3R,6E)-nerolidol and neryl acetate. There was also an interaction among the essential oil compositions citral and methyl palmitate, the target TNF, and rheumatoid arthritis. (Fig. **7C**) revealed that butyl isodecyl phthalate and methyl linoleate could be connected to Alzheimer's disease by acting on PTGES. Besides, CHRM1 was forecasted as the major target for the treatment of cognitive impairment, of which 32 essential oil compositions (isospathulenol, spathulenol, β-lonone, α-cadinol, torreyol, T-muurolol, globulol, (-)-cis-Carveol, (R)-(+)-citrone llal, geranyl linalool, phytol, isophytol, cadalin, caryophy llene oxide, (-)-γ-cadinene, (1R,4aR,8aS)-7-methyl-4-methy lidene-1-propan-2-yl-2,3,4a,5,6,8a-hexahydro-1H-naphthalene, (Z)-caryophyllene, β-caryophyllene, isocaryophyllene, α-copaene, α-cubebene, neral, terpinolene, D-limonene, β-elemene, citral, butyl isodecyl phthalate, farnesol, viridiflorol, (3R,6E)-nerolidol, geranyl acetone, and neryl acetate) were connected with them. As shown in (Fig. **7D**), 6 essential oil compositions could act on PLA2G4A and ALOX5 and be associated with asthma. These 6 essential oil compositions were methyl palmitate, oleamide, farnesol, methyllinolenate, butyl isodecyl phthalate and neryl acetate. 32 essential oil compositions (isospathulenol, spathulenol, β-lonone, α-cadinol, torreyol, T-muurolol, globulol, (-)-cis-carveol, geranyl linalool, (R)-(+)-citronellal, phytol, isophytol, cada lin, caryophyllene oxide, (-)-γ-cadinene, (1R,4aR,8aS)-7-methyl-4-methylidene-1-propan-2-yl-2, 3,4a,5,6,8a-hexahy dro-1H-naphthalene, (Z)-Caryophyllene, β-Caryophyllene, isocaryophyllene, α-copaene, α-cubebene, neral, terpinolene, D-limonene, β-elemene, citral, butyl isodecyl phthalate, farnesol, viridiflorol, (3R,6E)-nerolidol, geranyl acetone and neryl acetate) were predicted as the major ingredients in leaves of *C. grandis* ‘Tomentosa’ for the treatment of chronic obstructive pulmonary disease, and CHRM1 was regarded as the key target.

## 4 DISCUSSION

Traditional Chinese medicine is an important resource bank for developing innovative new drugs. However, Chinese compound medicine has the characteristics of multiple components, multiple targets, and multiple levels. Its mechanism is wide and difficult to elucidate, so all of these greatly limit the use and development of Chinese medicine. In recent years, combining Chinese medicine and network pharmacology has become a research hotspot, which contributes to systematically exploring the target and synergistic effects of the components of Chinese medicine, further realizing the development and modernization of Chinese medicine.

This study was to excavate the potential targets of the essential oil in leaves of *C. grandis* ‘Tomentosa,’ explore its pharmacological mechanism and predict its treatable diseases based on the network pharmacology method. The results showed that a total of 61 chemical components in the essential oil had their corresponding target proteins in the TCMS, Swiss Target Prediction, STITCH and SEA database, and a total of 679 potential targets were obtained. Many of these identified essential oils have been studied in *C. grandis* ‘Tomentosa’ fruits and exerted some pharmacological and beneficial properties like anti-microbial, anti-tumor, anti-oxidant, anti-inflammatory, cardiac stimulant, cytotoxic, hepatoprotective, nephroprotective, and anti-diabetic effects [21, 22]. After filtering by condition, 4 direct targets were found to be highly correlated with the essential oil composition, of which were PTGS2, CHRM1, GGPS1 and MAPK14. The PPI network was successfully constructed, which contained 54 nodes and 161 edges. Functional enrichment analysis showed 12, 19, and 12 GO entries related to biological processes, cell components and molecular functions, respectively. A total of 43 KEGG pathways were obtained, of which the most significant was terpenoid backbone biosynthesis. 22 diseases were achieved from TTD, mainly classified as tumors, immune, neurodegenerative, and respiratory diseases.

In the KEGG pathways, several experimental and clinical evidence reveal that TNF signaling pathway, toll-like receptor signaling pathway, VEGF signaling pathway, arachidonic acid metabolism, osteoclast differentiation, MAPK signaling pathway, IL-17 signaling pathway, Th17 cell differentiation, NF-kappa B signaling pathway, GnRH signaling pathway, relaxin signaling pathway, Epstein-Barr virus infection, human cytomegalovirus infection, herpes simplex infection, NOD-like receptor signaling pathway, apoptosis and viral carcinogenesis were involved in tumors, suggesting that these pathways may be the mechanisms of the essential oil from leaves of *C. grandis* ‘Tomentosa’ in treating tumors. Besides, TNF signaling pathway, IL-17 signaling pathway, toll-like receptor signaling pathway, VEGF signaling pathway, inflammatory bowel disease, MAPK signaling pathway, Th17 cell differentiation, NF-kappa B signaling pathway, Th1 and Th2 cell differentiation, Epstein-Barr virus infection, T cell receptor signaling pathway, human cytomegalovirus infection, NOD-like receptor signaling pathway, human immunodeficiency virus 1 infection and apoptosis were demonstrated to correlate with immune diseases. In addition, the potential target proteins of the essential oil in leaves of *C. grandis* ‘Tomentosa’ were enriched in TNF signaling pathway, toll-like receptor signaling pathway, amyotrophic lateral sclerosis, neurotrophin signaling pathway, human cytomegalovirus infection, apoptosis, which were involved in neurodegenerative diseases. Pertussis, Th17 cell differentiation, NF-kappa B signaling pathway, influenza A, T cell receptor signaling pathway, herpes simplex infection and apoptosis were involved in the signal transduction of respiratory diseases, which may also be the main mechanism of the essential oil in leaves of *C. grandis* ‘Tomentosa’ in treating respiratory diseases.

In these pathways, apoptosis was involved in the regulation of four diseases. Th17 cell differentiation and NF-kappa B signaling pathway were connected with 3 diseases, including tumors, immune diseases and respiratory diseases. TNF signaling pathway, toll-like receptor signaling pathway and human cytomegalovirus infection also connect with 3 diseases, including tumors, immune diseases and neurodegenerative diseases. Cell apoptosis, sometimes called programmed cell death, is an active process commanding mutated cells to commit suicide. Apoptosis plays an important role in several diseases, such as tumors/cancer, autoimmune diseases, AIDS, ischemia, and neurodegenerative diseases. Malignant tumors are generally believed to be caused by the uncontrolled growth of cells and excessive proliferation. From the perspective of cell apoptosis, it is believed that the occurrence of malignant tumors results from the inhibition of tumor apoptosis [23]. Autoreactive T lymphocytes and antibody-producing B lymphocytes are the main immunopathological mechanisms that cause autoimmune diseases. Under the stimulation of self-antigens, immune cells that recognize self-antigens are activated and eliminated by apoptosis [24]. However, if this mechanism is disturbed, the clearance of immune-competent cells that recognize self-antigens will be obstructed. Cohen observed that *Lpr* and *gld* mice developed lymphadenopathy and splenomegaly age-dependent by accumulating activated T and B lymphocytes [25]. Alzheimer's disease is an irreversible degenerative neurological disease caused by the acceleration of nerve cell apoptosis. Studies found that presenilin-1 (PS1) and presenilin-2 (PS2) mutations lead to familial Alzheimer's disease [26]. Meanwhile, presenilin was demonstrated to regulate neuronal apoptosis [27]. Apoptosis is also relevant to the pathogenesis of different respiratory diseases. Asthma has been demonstrated to be associated with defective activation of T-cell apoptosis through the FAS death receptor [28]. Reactive oxygen species-induced cell apoptosis has also been known to play a factor in the pathogenesis of acute respiratory distress syndrome [29].

In the network of component-target-disease, component-target-tumor network, component-target-immune disease network, component-target-neurodegenerative disease network and component-target-respiratory disease network were established. CHRM1 was regarded as the main target because 32 essential oil compositions could act on CHRM1 and be highly associated with cognitive impairment and chronic obstructive pulmonary disease. Pharmacological evidence suggests that cholinergic receptors are vital members of the cholinergic system, in which CHRM1 plays an important role in cognitive processes, hippocampal synaptic plasticity and neuronal excitability [30]. Some studies also found that CHRM1 was associated with bronchoconstriction of the airways, asthma, nicotine dependence and chronic obstructive pulmonary disease [31-33]. Farnesol was regarded as the most active essential oil composition in leaves of *C. grandis* ‘Tomentosa,’ which involved solid tumor/cancer, arthritis, cognitive impairment, asthma and chronic obstructive pulmonary disease. Farnesol, a natural terpene, is frequently found in essential oils [34]. A systematic review summarized that farnesol possessed broad pharmacological activities, including antimicrobial effects, preventing and treating cancer, promoting neuroprotective and behavioral effects, cardioprotective and hypotensive effects, and antioxidant and anti-inflammatory properties [35].

## CONCLUSION

In summary, based on network pharmacology, this study was to study the pharmacological activity and mechanism of the essential oil in leaves of *C. grandis* ‘Tomentosa.’ GC-MS/MS identified the specific chemical components of the essential oil, so the prediction accuracy is relatively high. The results revealed that the essential oil in leaves of *C. grandis* ‘Tomentosa’ could potentially treat tumors, immune diseases, neurodegenerative diseases and respiratory diseases by multi-pathways and multi-targets in which the most promising evidence was that farnesol might treat cognitive impairment and chronic obstructive pulmonary disease by regulating apoptosis *via* targeting CHRM1. However, further pharmacological experimental verification is needed.

The essential oils of leaves in *C. grandis* ‘Tomentosa’ have not been studied before. This study may provide new insight into *C. grandis* ‘Tomentosa’ or *C. grandis* (L.) Osbeck and may provide useful information for future utilization and development.

## AUTHORS’ CONTRIBUTIONS

YJS and CW created the study concept and design, acquired the data, conducted an analysis, interpreted the data, drafted the manuscript, critically revised the manuscript for important intellectual content, and completed the statistical analysis. HSC, CL and GZH conducted the GC-MS/MS experiment and analysed and interpreted the data.

Additionally, GF, managed the material, analysed and interpreted the data and critically revised the manuscript. Lastly, all authors read and approved the final manuscript.

## Figures and Tables

**Table 1:** Major essential oil components of leaves from *C. grandis* ‘Tomentosa’.

**No.**	**RT(min)**	**Compound**	**Content (%)**	**CAS**
1	6.071	Myrcene	0.67	123-35-3
2	6.780	D-Limonene	0.62	5989-27-5
3	6.929	trans-β-Ocimene	0.79	3779-61-1
4	7.861	Terpinolene	0.16	586-62-9
5	8.048	Linalool	0.19	78-70-6
6	9.059	(R)-(+)-Citronellal	0.23	2385-77-5
7	9.633	4-Terpineol	0.24	562-74-3
8	10.643	(-)-cis-Carveol	0.22	1197-06-4
9	10.935	β-Citronellol	5.21	106-22-9
10	10.973	Nerol	0.91	106-25-2
11	11.029	Isogeraniol	0.19	5944-20-7
12	11.228	Neral	0.35	106-26-3
13	11.641	Geraniol	4.12	106-24-1
14	12.008	Citral	0.55	141-27-5
15	13.953	1-Methyl-4-(1-methylethylidene)-2-(1-methylvinyl)-1-vinylcyclohexane	4.92	3242-08-8
16	14.261	α-Cubebene	0.37	17699-14-8
17	14.625	Neryl acetate	0.44	141-12-8
18	15.009	α-Copaene	0.47	3856-25-5
19	15.203	Geranyl acetate	0.35	105-87-3
20	15.498	β-Elemene	1.65	515-13-9
21	15.918	Isocaryophyllene	0.75	118-65-0
22	16.459	β-Caryophyllene	15.75	87-44-5
23	16.595	β-Copaene	1.83	18252-44-3
24	16.682	γ-Elemene	1.2	29873-99-2
25	16.856	(+)-Aromadenderne	1.08	489-39-4
26	16.962	Naphthalene, 1,2,3,4,4a,5,6,7-octahydro-1,4a-dimethyl-7-(1-methylethenyl)-, (1S,4aR,7R)-	0.43	52026-55-8
27	17.189	Geranylacetone	1.05	689-67-8
28	17.304	(Z)-Caryophyllene	4	6753-98-6
29	17.926	(1R,4aR,8aS)-7-methyl-4-methylidene-1-propan-2-yl-2,3,4a,5,6,8a-hexahydro-1H-naphthalene	1.37	30021-74-0
30	18.059	(-)-Germacrene D	1.44	23986-74-5
31	18.183	β-Lonone	0.47	79-77-6
32	18.603	Bicyclogermacrene	10.74	24703-35-3
33	18.799	α-Farnesene	0.78	502-61-4
34	18.986	(-)-γ-Cadinene	0.5	39029-41-9
35	19.278	(-)-δ-Cadinene	3.06	483-76-1
36	19.489	1,4-Cadinadiene	0.26	29837-12-5
37	20.049	Caryophyllene oxide	0.21	1139-30-6
38	20.179	Germacrene B	0.32	15423-57-1
39	20.586	(3R,6E)-Nerolidol	12.66	40716-66-3
40	20.941	Spathulenol	2.93	6750-60-3
41	21.071	Globulol	2.58	51371-47-2
42	21.31	Viridiflorol	0.75	552-02-3
43	22.529	Isospathulenol	0.69	88395-46-4
44	22.883	Spathulenol	0.39	77171-55-2
45	23.042	T-Muurolol	0.92	19912-62-0
46	23.182	Torreyol	0.24	19435-97-3
47	23.474	α-Cadinol	1.54	481-34-5
48	23.962	Cadalin	0.44	483-78-3
49	24.717	Tetradecanal	0.23	124-25-4
50	24.844	Farnesol	0.29	4602-84-0
51	25.081	(1S,3aR,4R,8R,8aS)-1-Isopropyl-3a-methyl-7-methylenedecahydro-4,8-epithioazulene	0.35	72445-42-2
52	25.146	Farnesal	0.21	502-67-0
53	26.243	Neophytadiene	0.39	504-96-1
54	26.977	Methyl palmitate	0.52	112-39-0
55	27.144	Isophytol	0.45	505-32-8
56	27.275	Butyl isodecyl phthalate	1.28	89-18-9
57	27.719	Geranyllinalool	0.21	1113-21-9
58	28.201	Methyllinolenate	0.37	301-00-8
59	28.319	Phytol	4.03	150-86-7
60	30.517	Oleamide	0.18	301-02-0
61	30.93	Bis(2-ethylhexyl) adipate	0.22	103-23-1

**Table 2:** The major targets of essential oil components in leaves from *C. grandis* ‘Tomentosa’ and its relevant topologicalparameters.

**Uniprot ID**	**Protein Name**	**Gene Name**	**Degree**	**Betweenness Centrality**	**Closeness Centrality**	**Average Shortest Path Length**
P35354	Prostaglandin G/H synthase 2	PTGS2	34	0.033479	0.425274	2.351425
P11229	Muscarinic acetylcholine receptor M1	CHRM1	32	0.035744	0.417564	2.394844
O95749	Geranylgeranyl pyrophosphate synthase	GGPS1	20	0.017712	0.405168	2.468114
Q16539	Mitogen-activated protein kinase 14	MAPK14	9	0.013179	0.413812	2.416554

**Table 3:** Diseases obtained from TTD based on the targets *via* PPI network analysis.

**Target**	**Disease**	**-**	**Target**	**Disease**
TNF	Rheumatoid arthritis; Intermittent claudication	-	DUSP1	Non-small-cell lung cancer
TP53	Oral cancer; Solid tumour/cancer	-	MAP2K1	Solid tumour/cancer
STAT3	Inflammation; Immune System disease	-	PTGES	Alzheimer disease; Neoplasm
PTGS2	Pain; Arthritis	-	HMOX1	Neonatal hyperbilirubinemia
MAPKAPK2	Inflammation; Autoimmune diabetes	-	PLA2G4A	Asthma
FDPS	Hypercalcaemia; Paget's disease	-	CDC25B	Solid tumour/cancer
IL1B	Immune System disease; Hypercalcaemia	-	CASP3	Solid tumour/cancer
CXCL8	Melanoma; Pain	-	MKNK1	Solid tumour/cancer; Colorectal cancer
ALOX5	Asthma; Lymphatic filariasis	-	CHRM1	Cognitive impairment; Chronic obstructive pulmonary disease

## Data Availability

All data generated or analysed during this study are included in this published article and its supplementary information files.

## LIST OF ABBREVIATIONS

GC-MS/MS: Gas Chromatography Coupled with Tandem Mass Spectrometry
GO: Gene Ontology
KEGG: Kyoto Encyclopedia of Genes and Genomes
PPT: Protein-Protein Interaction
SEA: Similarity Ensemble Approach
STRING: Search Tool for the Retrieval of Interacting Genes
TCMSP: Traditional Chinese Medicine Systems Pharmacology Database and Analysis Platform
TTD: Therapeutic Target Database
WebGestalt: WEB-based Gene SeT AnaLysis Toolkit

## RESEARCH INVOLVING PLANTS

The authors confirm that the fruit *C. grandis* used in this study is not endangered.
