# Supplementary material for: Analysis of Pharmacological Activities and Mechanisms of Essential Oil in Leaves of C. grandis ‘Tomentosa’ by GC-MS/MS and Network Pharmacology
Source: Comb Chem High Throughput Screen. 2023 Apr 27;26(9):1689–700. doi: 10.2174/1386207325666220610182644 (PMC10245803; doi:10.2174/1386207325666220610182644)
Supplement: Supplementary file 1 [file CCHTS-26-1689_SD1.docx]

Supplementary Material

Analysis of Pharmacological Activities and Mechanisms of Essential Oil in Leaves of *C. grandis* ‘Tomentosa’ by GC-MS/MS and Network Pharmacology

Jie-shu You^1,2^, Sheng-cai He^3^, Liang Chen^3^, Zhen-hui Guo^1^, Fei Gao^4^, Min-yue Zhang^5^, Liu Dan^6,*^, Wei Chen^1,*^

^1^School of Basic Medical Sciences, Guangzhou University of Chinese Medicine, Guangzhou, Guangdong Province, China; ^2^College of Pharmacy, Shenzhen Technology University, Shenzhen, Guangdong Province, China; ^3^School of Physical Education and Health, Guangzhou University of Chinese Medicine, Guangzhou, Guangdong Province, China; ^4^College of Pharmacy, Chengdu University of Traditional Chinese Medicine, Chengdu, Sichuan Province, China; ^5^Division of Hematology, Renji Hospital, School of Medicine, Shanghai Jiaotong University, Shanghai, China; ^6^Galactophore Department, Guangdong Provincial Hospital of Chinese Medicine, Guangzhou, Guangdong Province, China

Table S1. Information on potential targets of the essential oil composition in leaves of *C. grandis* ‘Tomentosa’.

| **Ingredients** | **Targets** | **Uniprot ID** | **Description** |
| --- | --- | --- | --- |
| NO.1 | ADH1C | [P00326](https://www.uniprot.org/uniprot/P00326) | Alcohol dehydrogenase 1C |
| NO.1 | GABRA1 | [P14867](https://www.uniprot.org/uniprot/P14867) | Gamma-aminobutyric acid receptor subunit alpha-1 |
| NO.1 | PPARA | Q07869 | Peroxisome proliferator-activated receptor alpha |
| NO.1 | CNR2 | P34972 | Cannabinoid receptor 2 |
| NO.1 | GGPS1 | O95749 | Geranylgeranyl pyrophosphate synthase |
| NO.1 | SQLE | Q14534 | Squalene monooxygenase |
| NO.2 | PTGS2 | P35354 | Prostaglandin G/H synthase 2 |
| NO.2 | GABRA1 | [P14867](https://www.uniprot.org/uniprot/P14867) | Gamma-aminobutyric acid receptor subunit alpha-1 |
| NO.2 | ADH1B | A0A024RDE3 | Alcohol dehydrogenase 1B |
| NO.2 | ADH1C | P00326 | Alcohol dehydrogenase 1C |
| NO.2 | NCOA2 | [Q15596](https://www.uniprot.org/uniprot/Q15596) | Nuclear receptor coactivator 2 |
| NO.2 | NCOA1 | Q15788 | Nuclear receptor coactivator 1 |
| NO.2 | CHRM2 | [P08172](https://www.uniprot.org/uniprot/P08172) | Muscarinic acetylcholine receptor M2 |
| NO.2 | GABRA2 | [P47869](https://www.uniprot.org/uniprot/P47869) | Gamma-aminobutyric-acid receptor alpha-2 subunit |
| NO.2 | CHRM1 | [P11229](https://www.uniprot.org/uniprot/P11229) | Muscarinic acetylcholine receptor M1 |
| NO.2 | GABRA5 | [P31644](https://www.uniprot.org/uniprot/P31644) | Gamma-aminobutyric-acid receptor alpha-5 subunit |
| NO.2 | GABRA3 | [P34903](https://www.uniprot.org/uniprot/P34903) | Gamma-aminobutyric-acid receptor alpha-3 subunit |
| NO.2 | IGHG1 | [P01857](https://www.uniprot.org/uniprot/P01857) | Ig gamma-1 chain C region |
| NO.2 | GABRA6 | [Q16445](https://www.uniprot.org/uniprot/Q16445) | Gamma-aminobutyric-acid receptor subunit alpha-6 |
| NO.2 | IPP | Q9Y573 | Actin-binding protein IPP |
| NO.2 | MTRR | Q9UBK8 | Methionine synthase reductase |
| NO.2 | POR | P16435 | NADPH-cytochrome P450 reductase |
| NO.2 | PPARG | P37231 | Peroxisome proliferator-activated receptor gamma |
| NO.2 | NOS1 | P29475 | Nitric oxide synthase, brain |
| NO.2 | NOS2 | P35228 | Nitric oxide synthase, inducible |
| NO.2 | NOS3 | P29474 | Nitric oxide synthase, endothelial |
| NO.2 | PPARA | Q07869 | Peroxisome proliferator-activated receptor alpha |
| NO.2 | CNR2 | P34972 | Cannabinoid receptor 2 |
| NO.2 | NR1H3 | Q13133 | LXR-alpha |
| NO.2 | CYP19A1 | P11511 | Cytochrome P450 19A1 |
| NO.3 | [PPARA](http://www.genecards.org/cgi-bin/carddisp.pl?gene=PPARA&search=PPARA) | [Q07869](http://www.uniprot.org/uniprot/Q07869) | Peroxisome proliferator-activated receptor alpha |
| NO.3 | [CNR2](http://www.genecards.org/cgi-bin/carddisp.pl?gene=CNR2&search=CNR2) | [P34972](http://www.uniprot.org/uniprot/P34972) | Cannabinoid receptor 2 |
| NO.4 | CHRM3 | [P20309](https://www.uniprot.org/uniprot/P20309) | Muscarinic acetylcholine receptor M3 |
| NO.4 | CHRM1 | [P11229](https://www.uniprot.org/uniprot/P11229) | Muscarinic acetylcholine receptor M1 |
| NO.4 | GABRA2 | [P47869](https://www.uniprot.org/uniprot/P47869) | Gamma-aminobutyric-acid receptor alpha-2 subunit |
| NO.4 | GABRA5 | [P31644](https://www.uniprot.org/uniprot/P31644) | Gamma-aminobutyric-acid receptor alpha-5 subunit |
| NO.4 | SLC6A2 | [P23975](https://www.uniprot.org/uniprot/P23975) | Sodium-dependent noradrenaline transporter |
| NO.4 | GABRA3 | [P34903](https://www.uniprot.org/uniprot/P34903) | Gamma-aminobutyric-acid receptor alpha-3 subunit |
| NO.4 | CHRM2 | [P08172](https://www.uniprot.org/uniprot/P08172) | Muscarinic acetylcholine receptor M2 |
| NO.4 | CHRNA2 | [Q15822](https://www.uniprot.org/uniprot/Q15822) | Neuronal acetylcholine receptor subunit alpha-2 |
| NO.4 | GABRA1 | [P14867](https://www.uniprot.org/uniprot/P14867) | Gamma-aminobutyric acid receptor subunit alpha-1 |
| NO.4 | IGHG1 | [P01857](https://www.uniprot.org/uniprot/P01857) | Ig gamma-1 chain C region |
| NO.4 | GABRA6 | [Q16445](https://www.uniprot.org/uniprot/Q16445) | Gamma-aminobutyric-acid receptor subunit alpha-6 |
| NO.4 | PTGS2 | P35354 | Prostaglandin G/H synthase 2 |
| NO.4 | ADH1A | [P07327](https://www.uniprot.org/uniprot/P07327) | Alcohol dehydrogenase 1A |
| NO.4 | ADH1B | A0A024RDE3 | Alcohol dehydrogenase 1B |
| NO.4 | ADH1C | P00326 | Alcohol dehydrogenase 1C |
| NO.4 | PPARA | Q07869 | Peroxisome proliferator-activated receptor alpha |
| NO.4 | CNR2 | P34972 | Cannabinoid receptor 2 |
| NO.5 | GABRA2 | [P47869](https://www.uniprot.org/uniprot/P47869) | Gamma-aminobutyric-acid receptor alpha-2 subunit |
| NO.5 | CHRM2 | [P08172](https://www.uniprot.org/uniprot/P08172) | Muscarinic acetylcholine receptor M2 |
| NO.5 | GABRA1 | [P14867](https://www.uniprot.org/uniprot/P14867) | Gamma-aminobutyric acid receptor subunit alpha-1 |
| NO.5 | GABRA6 | [Q16445](https://www.uniprot.org/uniprot/Q16445) | Gamma-aminobutyric-acid receptor subunit alpha-6 |
| NO.5 | GGPS1 | O95749 | Geranylgeranyl pyrophosphate synthase |
| NO.5 | MTRR | Q9UBK8 | Methionine synthase reductase |
| NO.5 | POR | P16435 | NADPH--cytochrome P450 reductase |
| NO.5 | TRPA1 | O75762 | Transient receptor potential cation channel subfamily A member 1 |
| NO.5 | NOS2 | P35228 | Nitric oxide synthase, inducible |
| NO.5 | NOS1 | P29475 | Nitric oxide synthase, brain |
| NO.5 | NOS3 | P29474 | Nitric oxide synthase, endothelial |
| NO.5 | TP53 | P04637 | Cellular tumor antigen p53 |
| NO.5 | HMGCR | P04035 | 3-hydroxy-3-methylglutaryl-coenzyme A reductase |
| NO.5 | CA2 | P00918 | Carbonic anhydrase II |
| NO.5 | CA1 | P00915 | Carbonic anhydrase I |
| NO.5 | CA4 | P22748 | Carbonic anhydrase IV |
| NO.5 | TRPV3 | Q8NET8 | Transient receptor potential cation channel subfamily V member 3 |
| NO.5 | TRPM8 | Q7Z2W7 | Transient receptor potential cation channel subfamily M member 8 |
| NO.5 | NR3C2 | P08235 | Mineralocorticoid receptor |
| NO.5 | NR3C1 | P04150 | Glucocorticoid receptor |
| NO.5 | PGR | P06401 | Progesterone receptor |
| NO.5 | SLC6A3 | Q01959 | Dopamine transporter (by homology) |
| NO.5 | SIGMAR1 | Q99720 | Sigma opioid receptor |
| NO.5 | HSD17B2 | P37059 | Estradiol 17-beta-dehydrogenase 2 |
| NO.5 | SQLE | Q14534 | Squalene monooxygenase |
| NO.5 | HMOX1 | P09601 | Heme oxygenase 1 (by homology) |
| NO.5 | IDO1 | P14902 | Indoleamine 2,3-dioxygenase |
| NO.5 | DRD2 | P14416 | Dopamine D2 receptor (by homology) |
| NO.5 | ADRA2C | P18825 | Adrenergic receptor alpha-2 |
| NO.5 | PTGS2 | P35354 | Cyclooxygenase-2 |
| NO.5 | CHRM4 | P08173 | Muscarinic acetylcholine receptor M4 |
| NO.5 | OPRM1 | P35372 | Mu opioid receptor |
| NO.5 | OPRD1 | P41143 | Delta opioid receptor |
| NO.5 | OPRK1 | P41145 | Kappa Opioid receptor |
| NO.5 | SCN5A | Q14524 | Sodium channel protein type V alpha subunit |
| NO.5 | SCN9A | Q15858 | Sodium channel protein type IX alpha subunit |
| NO.5 | KCNA5 | P22460 | Voltage-gated potassium channel subunit Kv1.5 |
| NO.5 | PTAFR | P25105 | Platelet activating factor receptor |
| NO.5 | PARP1 | P09874 | Poly [ADP-ribose] polymerase-1 |
| NO.5 | ADRA1A | P35348 | Alpha-1a adrenergic receptor |
| NO.5 | JAK1 | P23458 | Tyrosine-protein kinase JAK1 |
| NO.5 | JAK2 | O60674 | Tyrosine-protein kinase JAK2 |
| NO.5 | AR | P10275 | Androgen Receptor |
| NO.5 | MAPK8 | P45983 | c-Jun N-terminal kinase 1 |
| NO.5 | LRRK2 | Q5S007 | Leucine-rich repeat serine/threonine-protein kinase 2 |
| NO.5 | TNNC1 | P63316 | Troponin C, slow skeletal and cardiac muscles |
| NO.5 | TNNT2 | P45379 | Troponin T, cardiac muscle |
| NO.5 | TNNI3 | P19429 | Troponin I, cardiac muscle |
| NO.5 | TYMS | P04818 | Thymidylate synthase |
| NO.5 | HRH3 | Q9Y5N1 | Histamine H3 receptor |
| NO.5 | HRH4 | Q9H3N8 | Histamine H4 receptor |
| NO.5 | LTA4H | P09960 | Leukotriene A4 hydrolase |
| NO.6 | FAAH | O00519 | Anandamide amidohydrolase |
| NO.6 | CYP19A1 | P11511 | Cytochrome P450 19A1 |
| NO.6 | TRPV1 | Q8NER1 | Vanilloid receptor |
| NO.6 | SRD5A1 | P18405 | Steroid 5-alpha-reductase 1 |
| NO.6 | ADH1A | P07327 | Alcohol dehydrogenase alpha chain |
| NO.6 | ADH1B | P00325 | Alcohol dehydrogenase beta chain |
| NO.6 | ADH7 | P40394 | Alcohol dehydrogenase class IV |
| NO.6 | TRPA1 | O75762 | Transient receptor potential cation channel subfamily A member 1 |
| NO.6 | HLCS | P50747 | Biotin--protein ligase |
| NO.6 | MAOB | P27338 | Monoamine oxidase B |
| NO.6 | PGR | P06401 | Progesterone receptor |
| NO.6 | DRD2 | P14416 | Dopamine D2 receptor |
| NO.6 | MAOA | P21397 | Monoamine oxidase A |
| NO.6 | SIGMAR1 | Q99720 | Sigma opioid receptor |
| NO.6 | DRD4 | P21917 | Dopamine D4 receptor |
| NO.6 | PTGS1 | P23219 | Cyclooxygenase-1 |
| NO.6 | ALDH1A1 | P00352 | Aldehyde dehydrogenase 1A1 |
| NO.6 | CTSK | P43235 | Cathepsin K |
| NO.6 | CTSL | P07711 | Cathepsin L |
| NO.6 | CTSB | P07858 | Cathepsin (B and K) |
| NO.6 | SRD5A2 | P31213 | Steroid 5-alpha-reductase 2 |
| NO.6 | ABCG2 | Q9UNQ0 | ATP-binding cassette sub-family G member 2 |
| NO.6 | SIRT2 | Q8IXJ6 | NAD-dependent deacetylase sirtuin 2 |
| NO.6 | PPARG | P37231 | Peroxisome proliferator-activated receptor gamma |
| NO.6 | MTNR1A | P48039 | Melatonin receptor 1A |
| NO.6 | MTNR1B | P49286 | Melatonin receptor 1B |
| NO.6 | ADH1C | P00326 | Alcohol dehydrogenase gamma chain |
| NO.6 | PSEN2 | P49810 | Presenilin-2 |
| NO.6 | PSENEN | Q9NZ42 | Gamma-secretase subunit PEN-2 |
| NO.6 | NCSTN | Q92542 | Nicastrin |
| NO.6 | APH1A | Q96BI3 | Gamma-secretase subunit APH-1A |
| NO.6 | PSEN1 | P49768 | Presenilin-1 |
| NO.6 | APH1B | Q8WW43 | Gamma-secretase |
| NO.6 | PPARA | Q07869 | Peroxisome proliferator-activated receptor alpha |
| NO.6 | CTSD | P07339 | Cathepsin D |
| NO.6 | FABP5 | Q01469 | Fatty acid binding protein epidermal |
| NO.6 | PPARD | Q03181 | Peroxisome proliferator-activated receptor delta |
| NO.6 | FABP1 | P07148 | Fatty acid-binding protein, liver |
| NO.6 | AR | P10275 | Androgen Receptor |
| NO.6 | HDAC6 | Q9UBN7 | Histone deacetylase 6 |
| NO.6 | HDAC1 | Q13547 | Histone deacetylase 1 |
| NO.6 | MMP13 | P45452 | Matrix metalloproteinase 13 |
| NO.6 | PARP1 | P09874 | Poly [ADP-ribose] polymerase-1 |
| NO.6 | MMP1 | P03956 | Matrix metalloproteinase 1 |
| NO.6 | CHRM1 | P11229 | Muscarinic acetylcholine receptor M1 (by homology) |
| NO.6 | CACNA1B | Q00975 | Voltage-gated N-type calcium channel alpha-1B subunit |
| NO.6 | PSMB5 | P28074 | Proteasome Macropain subunit MB1 |
| NO.6 | CCND1 | P24385 | Cyclin-dependent cyclin D1 |
| NO.6 | CDK4 | P11802 | Cyclin-dependent kinase 4 |
| NO.6 | MMP8 | P22894 | Matrix metalloproteinase 8 |
| NO.6 | IKBKB | O14920 | Inhibitor of nuclear factor kappa B kinase beta subunit |
| NO.6 | EPHX2 | P34913 | Epoxide hydratase |
| NO.6 | JAK1 | P23458 | Tyrosine-protein kinase JAK1 |
| NO.6 | JAK2 | O60674 | Tyrosine-protein kinase JAK2 |
| NO.6 | FABP4 | P15090 | Fatty acid binding protein adipocyte |
| NO.6 | FABP3 | P05413 | Fatty acid binding protein muscle |
| NO.6 | DBF4 | Q9UBU7 | Protein DBF4 homolog A |
| NO.6 | CDC7 | O00311 | Cell division cycle 7-related protein kinase |
| NO.6 | CYP11B1 | P15538 | Cytochrome P450 11B1 |
| NO.6 | CYP11B2 | P19099 | Cytochrome P450 11B2 |
| NO.6 | HTR2A | P28223 | Serotonin 2a (5-HT2a) receptor |
| NO.6 | ADRB2 | [P07550](https://www.uniprot.org/uniprot/P07550) | Beta-2 adrenergic receptor |
| NO.6 | MSR1 | P21757 | Macrophage scavenger receptor types I and II |
| NO.6 | GGPS1 | O95749 | Geranylgeranyl pyrophosphate synthase |
| NO.7 | AR | P10275 | Androgen Receptor |
| NO.7 | CYP19A1 | P11511 | Cytochrome P450 19A1 |
| NO.7 | CA2 | P00918 | Carbonic anhydrase II |
| NO.7 | CA1 | P00915 | Carbonic anhydrase I |
| NO.7 | CA4 | P22748 | Carbonic anhydrase IV |
| NO.7 | RORC | P51449 | Nuclear receptor ROR-gamma |
| NO.7 | SREBF2 | Q12772 | Sterol regulatory element-binding protein 2 |
| NO.7 | NPC1L1 | Q9UHC9 | Niemann-Pick C1-like protein 1 |
| NO.7 | NR1H3 | Q13133 | LXR-alpha |
| NO.7 | HMGCR | P04035 | HMG-CoA reductase |
| NO.7 | ESR2 | Q92731 | Estrogen receptor beta |
| NO.7 | TRPM8 | Q7Z2W7 | Transient receptor potential cation channel subfamily M member 8 |
| NO.7 | CHRM2 | P08172 | Muscarinic acetylcholine receptor M2 |
| NO.7 | CYP17A1 | P05093 | Cytochrome P450 17A1 |
| NO.7 | PTPN1 | P18031 | Protein-tyrosine phosphatase 1B |
| NO.7 | DRD2 | P14416 | Dopamine D2 receptor (by homology) |
| NO.7 | RORA | P35398 | Nuclear receptor ROR-alpha |
| NO.7 | SQLE | Q14534 | Squalene monooxygenase |
| NO.7 | CYP51A1 | Q16850 | Cytochrome P450 51 |
| NO.7 | PPARA | Q07869 | Peroxisome proliferator-activated receptor alpha |
| NO.7 | PPARD | Q03181 | Peroxisome proliferator-activated receptor delta |
| NO.7 | SLC6A4 | P31645 | Serotonin transporter |
| NO.7 | G6PD | P11413 | Glucose-6-phosphate 1-dehydrogenase |
| NO.7 | ACHE | P22303 | Acetylcholinesterase |
| NO.7 | ESR1 | P03372 | Estrogen receptor alpha |
| NO.7 | ADRA2C | P18825 | Adrenergic receptor alpha-2 |
| NO.7 | HSD17B2 | P37059 | Estradiol 17-beta-dehydrogenase 2 |
| NO.7 | NR3C1 | P04150 | Glucocorticoid receptor |
| NO.7 | SHBG | P04278 | Testis-specific androgen-binding protein |
| NO.7 | SLC6A2 | P23975 | Norepinephrine transporter |
| NO.7 | NR3C2 | P08235 | Mineralocorticoid receptor |
| NO.7 | BCHE | P06276 | Butyrylcholinesterase |
| NO.7 | PTPRF | P10586 | Receptor-type tyrosine-protein phosphatase F (LAR) |
| NO.7 | PTPN2 | P17706 | T-cell protein-tyrosine phosphatase |
| NO.7 | PLA2G1B | P04054 | Phospholipase A2 group 1B |
| NO.7 | ACP1 | P24666 | Low molecular weight phosphotyrosine protein phosphatase |
| NO.7 | AKR1B10 | O60218 | Aldo-keto reductase family 1 member B10 |
| NO.7 | GLI2 | P10070 | Zinc finger protein GLI2 |
| NO.7 | GLI1 | P08151 | Zinc finger protein GLI1 |
| NO.7 | GABRA2 | [P47869](https://www.uniprot.org/uniprot/P47869) | Gamma-aminobutyric-acid receptor alpha-2 subunit |
| NO.7 | GABRA1 | [P14867](https://www.uniprot.org/uniprot/P14867) | Gamma-aminobutyric acid receptor subunit alpha-1 |
| NO.8 | GABRA2 | [P47869](https://www.uniprot.org/uniprot/P47869) | Gamma-aminobutyric-acid receptor alpha-2 subunit |
| NO.8 | SLC6A2 | [P23975](https://www.uniprot.org/uniprot/P23975) | Sodium-dependent noradrenaline transporter |
| NO.8 | CHRM2 | [P08172](https://www.uniprot.org/uniprot/P08172) | Muscarinic acetylcholine receptor M2 |
| NO.8 | GABRA1 | [P14867](https://www.uniprot.org/uniprot/P14867) | Gamma-aminobutyric acid receptor subunit alpha-1 |
| NO.8 | CHRM1 | [P11229](https://www.uniprot.org/uniprot/P11229) | Muscarinic acetylcholine receptor M1 |
| NO.8 | GABRA3 | [P34903](https://www.uniprot.org/uniprot/P34903) | Gamma-aminobutyric-acid receptor alpha-3 subunit |
| NO.8 | ADRA2C | P18825 | Adrenergic receptor alpha-2 |
| NO.8 | AR | P10275 | Androgen Receptor |
| NO.8 | CA1 | P00915 | Carbonic anhydrase I |
| NO.8 | CA2 | P00918 | Carbonic anhydrase II |
| NO.8 | CA4 | P22748 | Carbonic anhydrase IV |
| NO.8 | CHRM4 | P08173 | Muscarinic acetylcholine receptor M4 |
| NO.8 | CYP19A1 | P11511 | Cytochrome P450 19A1 |
| NO.8 | DRD2 | P14416 | Dopamine D2 receptor (by homology) |
| NO.8 | ESR2 | Q92731 | Estrogen receptor beta |
| NO.8 | HMOX1 | P09601 | Heme oxygenase 1 (by homology) |
| NO.8 | HSD17B2 | P37059 | Estradiol 17-beta-dehydrogenase 2 |
| NO.8 | KCNA5 | P22460 | Voltage-gated potassium channel subunit Kv1.5 |
| NO.8 | NPC1L1 | Q9UHC9 | Niemann-Pick C1-like protein 1 |
| NO.8 | NR1H3 | Q13133 | LXR-alpha |
| NO.8 | NR3C1 | P04150 | Glucocorticoid receptor |
| NO.8 | PTAFR | P25105 | Platelet activating factor receptor |
| NO.8 | RORA | P35398 | Nuclear receptor ROR-alpha |
| NO.8 | SHBG | P04278 | Testis-specific androgen-binding protein |
| NO.8 | SQLE | Q14534 | Squalene monooxygenase |
| NO.8 | TOP1 | P11387 | DNA topoisomerase I |
| NO.8 | TRPM8 | Q7Z2W7 | Transient receptor potential cation channel subfamily M member 8 |
| NO.8 | TRPV3 | Q8NET8 | Transient receptor potential cation channel subfamily V member 3 |
| NO.9 | ADH1C | P00326 | Alcohol dehydrogenase 1C |
| NO.9 | PTGS2 | P35354 | Prostaglandin G/H synthase 2 |
| NO.9 | NCOA2 | [Q15596](https://www.uniprot.org/uniprot/Q15596) | Nuclear receptor coactivator 2 |
| NO.9 | AR | P10275 | Androgen Receptor |
| NO.9 | CYP19A1 | P11511 | Cytochrome P450 19A1 |
| NO.9 | SQLE | Q14534 | Squalene monooxygenase |
| NO.9 | SLC6A2 | P23975 | Norepinephrine transporter |
| NO.9 | BCHE | P06276 | Butyrylcholinesterase |
| NO.9 | HMGCR | P04035 | HMG-CoA reductase |
| NO.9 | NR1I3 | Q14994 | Nuclear receptor subfamily 1 group I member 3 |
| NO.9 | ESR1 | P03372 | Estrogen receptor alpha |
| NO.9 | SLC6A4 | P31645 | Serotonin transporter |
| NO.9 | CHRM2 | P08172 | Muscarinic acetylcholine receptor M2 |
| NO.9 | CYP2C19 | P33261 | Cytochrome P450 2C19 |
| NO.9 | CYP17A1 | P05093 | Cytochrome P450 17A1 |
| NO.9 | HSD11B1 | P28845 | 11-beta-hydroxysteroid dehydrogenase 1 |
| NO.9 | PTGS1 | P23219 | Cyclooxygenase-1 |
| NO.9 | EPHX2 | P34913 | Epoxide hydratase |
| NO.9 | RORC | P51449 | Nuclear receptor ROR-gamma |
| NO.9 | SREBF2 | Q12772 | Sterol regulatory element-binding protein 2 |
| NO.9 | NPC1L1 | Q9UHC9 | Niemann-Pick C1-like protein 1 |
| NO.9 | FABP4 | P15090 | Fatty acid binding protein adipocyte |
| NO.9 | PPARA | Q07869 | Peroxisome proliferator-activated receptor alpha |
| NO.9 | TERT | O14746 | Telomerase reverse transcriptase |
| NO.9 | FABP3 | P05413 | Fatty acid binding protein muscle |
| NO.9 | FABP5 | Q01469 | Fatty acid binding protein epidermal |
| NO.9 | PPARD | Q03181 | Peroxisome proliferator-activated receptor delta |
| NO.9 | FABP1 | P07148 | Fatty acid-binding protein, liver |
| NO.9 | CYP51A1 | Q16850 | Cytochrome P450 51 (by homology) |
| NO.9 | KCNA3 | P22001 | Voltage-gated potassium channel subunit Kv1.3 |
| NO.9 | ACHE | P22303 | Acetylcholinesterase |
| NO.9 | SCD | O00767 | Acyl-CoA desaturase |
| NO.9 | JAK1 | P23458 | Tyrosine-protein kinase JAK1 |
| NO.9 | JAK2 | O60674 | Tyrosine-protein kinase JAK2 |
| NO.9 | MSR1 | P21757 | Macrophage scavenger receptor types I and II |
| NO.10 | ADH1C | P00326 | Alcohol dehydrogenase 1C |
| NO.10 | DDC | P20711 | Aromatic-L-amino-acid decarboxylase |
| NO.10 | CDK2 | P24941 | Cyclin-dependent kinase 2 |
| NO.10 | IPP | Q9Y573 | Actin-binding protein IPP |
| NO.10 | SQLE | Q14534 | Squalene monooxygenase |
| NO.10 | PTGS1 | P23219 | Cyclooxygenase-1 |
| NO.10 | PTGS2 | P35354 | Cyclooxygenase-2 |
| NO.10 | PGR | P06401 | Progesterone receptor |
| NO.10 | HMGCR | P04035 | HMG-CoA reductase (by homology) |
| NO.10 | KCNH2 | Q12809 | HERG |
| NO.10 | UGT2B7 | P16662 | UDP-glucuronosyltransferase 2B7 |
| NO.10 | EPHX2 | P34913 | Epoxide hydratase |
| NO.10 | JAK1 | P23458 | Tyrosine-protein kinase JAK1 |
| NO.10 | JAK2 | O60674 | Tyrosine-protein kinase JAK2 |
| NO.10 | CYP11B1 | P15538 | Cytochrome P450 11B1 |
| NO.10 | CYP11B2 | P19099 | Cytochrome P450 11B2 |
| NO.10 | PIM1 | P11309 | Serine/threonine-protein kinase PIM1 |
| NO.10 | PIM3 | Q86V86 | Serine/threonine-protein kinase PIM3 |
| NO.10 | GGPS1 | O95749 | Geranylgeranyl pyrophosphate synthase |
| NO.10 | LSS | P48449 | Lanosterol synthase |
| NO.10 | FNTB | P49356 | Protein farnesyltransferase subunit beta |
| NO.10 | FNTA | P49354 | Protein farnesyltransferase/geranylgeranyltransferase type-1 subunit alpha |
| NO.10 | CDC25C | P30307 | M-phase inducer phosphatase 3 |
| NO.11 | DRD2 | P14416 | Dopamine D2 receptor (by homology) |
| NO.11 | SIGMAR1 | Q99720 | Sigma opioid receptor (by homology) |
| NO.11 | PTGS1 | P23219 | Cyclooxygenase-1 |
| NO.11 | PTGS2 | P35354 | Cyclooxygenase-2 |
| NO.11 | CA2 | P00918 | Carbonic anhydrase II |
| NO.11 | CA1 | P00915 | Carbonic anhydrase I |
| NO.11 | CA4 | P22748 | Carbonic anhydrase IV |
| NO.11 | EPHX2 | P34913 | Epoxide hydratase |
| NO.11 | IDO1 | P14902 | Indoleamine 2,3-dioxygenase |
| NO.11 | NR3C1 | P04150 | Glucocorticoid receptor |
| NO.11 | JAK1 | P23458 | Tyrosine-protein kinase JAK1 |
| NO.11 | JAK2 | O60674 | Tyrosine-protein kinase JAK2 |
| NO.11 | KCNH2 | Q12809 | HERG |
| NO.11 | JAK3 | P52333 | Tyrosine-protein kinase JAK3 |
| NO.11 | TYK2 | P29597 | Tyrosine-protein kinase TYK2 |
| NO.11 | SQLE | Q14534 | Squalene monooxygenase |
| NO.11 | GGPS1 | O95749 | Geranylgeranyl pyrophosphate synthase |
| NO.12 | ALDH1A1 | P00352 | Aldehyde dehydrogenase 1A1 |
| NO.12 | DRD2 | P14416 | Dopamine D2 receptor |
| NO.12 | ADH1A | P07327 | Alcohol dehydrogenase alpha chain |
| NO.12 | MAOA | P21397 | Monoamine oxidase A |
| NO.12 | MAOB | P27338 | Monoamine oxidase B |
| NO.12 | CHRM2 | P08172 | Muscarinic acetylcholine receptor M2 |
| NO.12 | CHRM1 | P11229 | Muscarinic acetylcholine receptor M1 |
| NO.12 | CHRM3 | P20309 | Muscarinic acetylcholine receptor M3 |
| NO.12 | ADH1B | P00325 | Alcohol dehydrogenase beta chain |
| NO.12 | ADH7 | P40394 | Alcohol dehydrogenase class IV |
| NO.12 | DRD4 | P21917 | Dopamine D4 receptor |
| NO.12 | SRD5A1 | P18405 | Steroid 5-alpha-reductase 1 |
| NO.12 | CCND1 | P24385 | G1/S-specific cyclin-D1 |
| NO.12 | CDK4 | P11802 | Cyclin-dependent kinase 4 |
| NO.12 | DBF4 | Q9UBU7 | Protein DBF4 homolog A |
| NO.12 | CDC7 | O00311 | Cell division cycle 7-related protein kinase |
| NO.12 | CYP2C19 | P33261 | Cytochrome P450 2C19 |
| NO.12 | PTGS2 | P35354 | Cyclooxygenase-2 |
| NO.12 | CYP2A6 | P11509 | Cytochrome P450 2A6 |
| NO.12 | SIRT2 | Q8IXJ6 | NAD-dependent deacetylase sirtuin 2 |
| NO.12 | FABP4 | P15090 | Fatty acid binding protein adipocyte |
| NO.12 | PSEN2 | P49810 | Presenilin-2 |
| NO.12 | PSENEN | Q9NZ42 | Gamma-secretase subunit PEN-2 |
| NO.12 | NCSTN | Q92542 | Nicastrin |
| NO.12 | APH1A | Q96BI3 | Gamma-secretase subunit APH-1A |
| NO.12 | PSEN1 | P49768 | Presenilin-1 |
| NO.12 | APH1B | Q8WW43 | Gamma-secretase subunit APH-1B |
| NO.12 | FABP3 | P05413 | Fatty acid binding protein muscle |
| NO.12 | GSR | P00390 | Glutathione reductase |
| NO.12 | SQLE | Q14534 | Squalene monooxygenase |
| NO.12 | GGPS1 | O95749 | Geranylgeranyl pyrophosphate synthase |
| NO.12 | LSS | P48449 | Lanosterol synthase |
| NO.12 | FNTB | P49356 | Protein farnesyltransferase subunit beta |
| NO.12 | FNTA | P49354 | Protein farnesyltransferase/geranylgeranyltransferase type-1 subunit alpha |
| NO.13 | ADH1B | A0A024RDE3 | Alcohol dehydrogenase 1B |
| NO.13 | ADH1C | P00326 | Alcohol dehydrogenase 1C |
| NO.13 | PGR | [P06401](https://www.uniprot.org/uniprot/P06401) | Progesterone receptor |
| NO.13 | CCND1 | P24385 | G1/S-specific cyclin-D1 |
| NO.13 | MAPK3 | [P27361](https://www.uniprot.org/uniprot/P27361) | Mitogen-activated protein kinase 3 |
| NO.13 | CDK4 | P11802 | Cell division protein kinase 4 |
| NO.13 | BAK1 | [Q16611](https://www.uniprot.org/uniprot/Q16611) | Bcl-2 homologous antagonist/killer |
| NO.13 | PRKCB | [P05771](https://www.uniprot.org/uniprot/P05771) | Protein kinase C beta type |
| NO.13 | HMGCR | [P04035](https://www.uniprot.org/uniprot/P04035) | 3-hydroxy-3-methylglutaryl-coenzyme A reductase |
| NO.13 | CYP2B6 | [P20813](https://www.uniprot.org/uniprot/P20813) | Cytochrome P450 2B6 |
| NO.13 | SI | [P14410](https://www.uniprot.org/uniprot/P14410) | Sucrase-isomaltase, intestinal |
| NO.13 | LCT | [P09848](https://www.uniprot.org/uniprot/P09848) | Lactase-phlorizin hydrolase |
| NO.13 | SQLE | Q14534 | Squalene monooxygenase |
| NO.13 | PTGS1 | P23219 | Cyclooxygenase-1 |
| NO.13 | PTGS2 | P35354 | Cyclooxygenase-2 |
| NO.13 | KCNH2 | Q12809 | HERG |
| NO.13 | UGT2B7 | P16662 | UDP-glucuronosyltransferase 2B7 |
| NO.13 | EPHX2 | P34913 | Epoxide hydratase |
| NO.13 | JAK1 | P23458 | Tyrosine-protein kinase JAK1 |
| NO.13 | JAK2 | O60674 | Tyrosine-protein kinase JAK2 |
| NO.13 | CYP11B1 | P15538 | Cytochrome P450 11B1 |
| NO.13 | CYP11B2 | P19099 | Cytochrome P450 11B2 |
| NO.13 | PIM1 | P11309 | Serine/threonine-protein kinase PIM1 |
| NO.13 | PIM3 | Q86V86 | Serine/threonine-protein kinase PIM3 |
| NO.13 | DDC | P20711 | Aromatic-L-amino-acid decarboxylase |
| NO.13 | CDK2 | P24941 | Cyclin-dependent kinase 2 |
| NO.13 | IPP | Q9Y573 | Actin-binding protein IPP |
| NO.13 | GGPS1 | O95749 | Geranylgeranyl pyrophosphate synthase |
| NO.13 | LSS | P48449 | Lanosterol synthase |
| NO.13 | FNTB | P49356 | Protein farnesyltransferase subunit beta |
| NO.13 | FNTA | P49354 | Protein farnesyltransferase/geranylgeranyltransferase type-1 subunit alpha |
| NO.13 | CDC25C | P30307 | M-phase inducer phosphatase 3 |
| NO.14 | RELA | [Q04206](https://www.uniprot.org/uniprot/Q04206) | Transcription factor p65 |
| NO.14 | TNF | [P01375](https://www.uniprot.org/uniprot/P01375) | Tumor necrosis factor |
| NO.14 | NFKBIA | [O15111](https://www.uniprot.org/uniprot/O15111) | NF-kappa-B inhibitor alpha |
| NO.14 | CYP2B6 | [P20813](https://www.uniprot.org/uniprot/P20813) | Cytochrome P450 2B6 |
| NO.14 | ADH1A | P07327 | Alcohol dehydrogenase alpha chain |
| NO.14 | ADH1B | P00325 | Alcohol dehydrogenase beta chain |
| NO.14 | ADH7 | P40394 | Alcohol dehydrogenase class IV |
| NO.14 | ALDH1A1 | P00352 | Aldehyde dehydrogenase 1A1 |
| NO.14 | CCND1 | P24385 | G1/S-specific cyclin-D1 |
| NO.14 | CDK4 | P11802 | Cyclin-dependent kinase 4 |
| NO.14 | CHRM1 | P11229 | Muscarinic acetylcholine receptor M1 |
| NO.14 | CHRM2 | P08172 | Muscarinic acetylcholine receptor M2 |
| NO.14 | CHRM3 | P20309 | Muscarinic acetylcholine receptor M3 |
| NO.14 | CYP2A6 | P11509 | Cytochrome P450 2A6 |
| NO.14 | CYP2C19 | P33261 | Cytochrome P450 2C19 |
| NO.14 | DBF4 | Q9UBU7 | Protein DBF4 homolog A |
| NO.14 | CDC7 | O00311 | Cell division cycle 7-related protein kinase |
| NO.14 | DRD2 | P14416 | Dopamine D2 receptor |
| NO.14 | DRD4 | P21917 | Dopamine D4 receptor |
| NO.14 | FABP3 | P05413 | Fatty acid binding protein muscle |
| NO.14 | FABP4 | P15090 | Fatty acid binding protein adipocyte |
| NO.14 | GSR | P00390 | Glutathione reductase |
| NO.14 | MAOA | P21397 | Monoamine oxidase A |
| NO.14 | MAOB | P27338 | Monoamine oxidase B |
| NO.14 | PSEN2 | P49810 | Presenilin-2 |
| NO.14 | PSENEN | Q9NZ42 | Gamma-secretase subunit PEN-2 |
| NO.14 | NCSTN | Q92542 | Nicastrin |
| NO.14 | APH1A | Q96BI3 | Gamma-secretase subunit APH-1A |
| NO.14 | PSEN1 | P49768 | Presenilin-1 |
| NO.14 | APH1B | Q8WW43 | Gamma-secretase |
| NO.14 | PTGS2 | P35354 | Cyclooxygenase-2 |
| NO.14 | SIRT2 | Q8IXJ6 | NAD-dependent deacetylase sirtuin 2 |
| NO.14 | SRD5A1 | P18405 | Steroid 5-alpha-reductase 1 |
| NO.14 | SQLE | Q14534 | Squalene monooxygenase |
| NO.14 | GGPS1 | O95749 | Geranylgeranyl pyrophosphate synthase |
| NO.14 | LSS | P48449 | Lanosterol synthase |
| NO.14 | FNTB | P49356 | Protein farnesyltransferase subunit beta |
| NO.14 | FNTA | P49354 | Protein farnesyltransferase/geranylgeranyltransferase type-1 subunit alpha |
| NO.15 | [PPARA](http://www.genecards.org/cgi-bin/carddisp.pl?gene=PPARA&search=PPARA) | [Q07869](http://www.uniprot.org/uniprot/Q07869) | Peroxisome proliferator-activated receptor alpha |
| NO.15 | CNR2 | [P34972](http://www.uniprot.org/uniprot/P34972) | Cannabinoid receptor 2 |
| NO.15 | [CXCR3](http://www.genecards.org/cgi-bin/carddisp.pl?gene=CXCR3&search=CXCR3) | [P49682](http://www.uniprot.org/uniprot/P49682) | C-X-C chemokine receptor type 3 |
| NO.15 | [NR1H3](http://www.genecards.org/cgi-bin/carddisp.pl?gene=NR1H3&search=NR1H3) | Q13133 | LXR-alpha |
| NO.16 | CHRM3 | [P20309](https://www.uniprot.org/uniprot/P20309) | Muscarinic acetylcholine receptor M3 |
| NO.16 | CHRM1 | [P11229](https://www.uniprot.org/uniprot/P11229) | Muscarinic acetylcholine receptor M1 |
| NO.16 | PTGS2 | P35354 | Prostaglandin G/H synthase 2 |
| NO.16 | CHRM2 | [P08172](https://www.uniprot.org/uniprot/P08172) | Muscarinic acetylcholine receptor M2 |
| NO.16 | CHRNA2 | [Q15822](https://www.uniprot.org/uniprot/Q15822) | Neuronal acetylcholine receptor subunit alpha-2 |
| NO.16 | GABRA1 | [P14867](https://www.uniprot.org/uniprot/P14867) | Gamma-aminobutyric acid receptor subunit alpha-1 |
| NO.16 | BCHE | P06276 | Butyrylcholinesterase |
| NO.16 | PPARA | Q07869 | Peroxisome proliferator-activated receptor alpha |
| NO.16 | CNR2 | P34972 | Cannabinoid receptor 2 |
| NO.16 | NR1I3 | Q14994 | Nuclear receptor subfamily 1 group I member 3 (by homology) |
| NO.16 | AR | P10275 | Androgen Receptor (by homology) |
| NO.16 | CYP19A1 | P11511 | Cytochrome P450 19A1 |
| NO.16 | ESR1 | P03372 | Estrogen receptor alpha |
| NO.16 | ACHE | P22303 | Acetylcholinesterase |
| NO.16 | SLC6A2 | P23975 | Norepinephrine transporter |
| NO.16 | SLC6A4 | P31645 | Serotonin transporter |
| NO.16 | CYP2C19 | P33261 | Cytochrome P450 2C19 |
| NO.17 | PTGS1 | [P23219](https://www.uniprot.org/uniprot/P23219) | Prostaglandin G/H synthase 1 |
| NO.17 | CHRM3 | [P20309](https://www.uniprot.org/uniprot/P20309) | Muscarinic acetylcholine receptor M3 |
| NO.17 | CHRM1 | [P11229](https://www.uniprot.org/uniprot/P11229) | Muscarinic acetylcholine receptor M1 |
| NO.17 | SCN5A | [Q14524](https://www.uniprot.org/uniprot/Q14524) | Sodium channel protein type 5 subunit alpha |
| NO.17 | PTGS2 | P35354 | Prostaglandin G/H synthase 2 |
| NO.17 | PDE3A | [Q14432](https://www.uniprot.org/uniprot/Q14432) | CGMP-inhibited 3',5'-cyclic phosphodiesterase A |
| NO.17 | ADRA1B | [P35368](https://www.uniprot.org/uniprot/P35368) | Alpha-1B adrenergic receptor |
| NO.17 | ADRB2 | [P07550](https://www.uniprot.org/uniprot/P07550) | Beta-2 adrenergic receptor |
| NO.17 | SLC6A4 | [P31645](https://www.uniprot.org/uniprot/P31645) | Sodium-dependent serotonin transporter |
| NO.17 | LTA4H | [P09960](https://www.uniprot.org/uniprot/P09960) | Leukotriene A-4 hydrolase |
| NO.17 | MAOB | [P27338](https://www.uniprot.org/uniprot/P27338) | Amine oxidase [flavin-containing] B |
| NO.17 | CHRNA7 | [P36544](https://www.uniprot.org/uniprot/P36544) | Neuronal acetylcholine receptor protein, alpha-7 chain |
| NO.17 | PRKACA | [P17612](https://www.uniprot.org/uniprot/P17612) | mRNA of PKA Catalytic Subunit C-alpha |
| NO.17 | GRIA2 | P42262 | Glutamate receptor 2 |
| NO.17 | ACHE | P22303 | Acetylcholinesterase |
| NO.17 | NPY5R | Q15761 | Neuropeptide Y receptor type 5 |
| NO.17 | HSD11B1 | P28845 | 11-beta-hydroxysteroid dehydrogenase 1 |
| NO.17 | EPHX1 | P07099 | Epoxide hydrolase 1 |
| NO.17 | CYP17A1 | P05093 | Cytochrome P450 17A1 |
| NO.17 | F2R | P25116 | Proteinase-activated receptor 1 |
| NO.17 | F2 | P00734 | Thrombin |
| NO.17 | PRSS1 | P07477 | Trypsin I |
| NO.17 | EGFR | P00533 | Epidermal growth factor receptor erbB1 |
| NO.17 | GSK3B | P49841 | Glycogen synthase kinase-3 beta |
| NO.17 | GABRB3 | P28472 | Gamma-aminobutyric acid receptor subunit beta-3 |
| NO.17 | GABRA3 | P34903 | Gamma-aminobutyric acid receptor subunit alpha-3 |
| NO.17 | GABRG2 | P18507 | Gamma-aminobutyric acid receptor subunit gamma-2 |
| NO.17 | GABRA1 | P14867 | Gamma-aminobutyric acid receptor subunit alpha-1 |
| NO.17 | GABRA5 | P31644 | Gamma-aminobutyric acid receptor subunit alpha-5 |
| NO.17 | GABRA2 | P47869 | Gamma-aminobutyric acid receptor subunit alpha-2 |
| NO.17 | MMP9 | P14780 | Matrix metalloproteinase 9 |
| NO.17 | MMP1 | P03956 | Matrix metalloproteinase 1 |
| NO.17 | MMP2 | P08253 | Matrix metalloproteinase 2 |
| NO.17 | TNKS2 | Q9H2K2 | Tankyrase-2 |
| NO.17 | ALOX5 | P09917 | Arachidonate 5-lipoxygenase |
| NO.17 | MAPK10 | P53779 | c-Jun N-terminal kinase 3 |
| NO.17 | HRH3 | Q9Y5N1 | Histamine H3 receptor |
| NO.17 | LRRK2 | Q5S007 | Leucine-rich repeat serine/threonine-protein kinase 2 |
| NO.17 | JAK3 | P52333 | Tyrosine-protein kinase JAK3 |
| NO.17 | JAK1 | P23458 | Tyrosine-protein kinase JAK1 |
| NO.17 | JAK2 | O60674 | Tyrosine-protein kinase JAK2 |
| NO.17 | MAPK9 | P45984 | c-Jun N-terminal kinase 2 |
| NO.17 | CYP11B1 | P15538 | Cytochrome P450 11B1 |
| NO.17 | CA2 | P00918 | Carbonic anhydrase II |
| NO.17 | CYP11B2 | P19099 | Cytochrome P450 11B2 |
| NO.17 | CA4 | P22748 | Carbonic anhydrase IV |
| NO.17 | CSF1R | P07333 | Macrophage colony stimulating factor receptor |
| NO.17 | CES1 | P23141 | Acyl coenzyme A: cholesterol acyltransferase |
| NO.17 | CTSK | P43235 | Cathepsin K |
| NO.17 | CES2 | O00748 | Carboxylesterase 2 |
| NO.17 | GRM2 | Q14416 | Metabotropic glutamate receptor 2 (by homology) |
| NO.17 | BRAF | P15056 | Serine/threonine-protein kinase B-raf |
| NO.17 | ABHD6 | Q9BV23 | Monoacylglycerol lipase ABHD6 |
| NO.17 | CNR1 | P21554 | Cannabinoid receptor 1 (by homology) |
| NO.17 | FAAH | O00519 | Anandamide amidohydrolase |
| NO.17 | MET | P08581 | Hepatocyte growth factor receptor |
| NO.17 | MGLL | Q99685 | Monoglyceride lipase |
| NO.17 | HTR2A | P28223 | Serotonin 2a (5-HT2a) receptor |
| NO.17 | HTR2C | P28335 | Serotonin 2c (5-HT2c) receptor |
| NO.17 | MAPK14 | Q16539 | MAP kinase p38 alpha (by homology) |
| NO.17 | CTSH | P09668 | Cathepsin (H and K) |
| NO.17 | HTR6 | P50406 | Serotonin 6 (5-HT6) receptor |
| NO.17 | CTSL | P07711 | Cathepsin L |
| NO.17 | CTSB | P07858 | Cathepsin (B and K) |
| NO.17 | TAAR1 | Q96RJ0 | Trace amine-associated receptor 1 (by homology) |
| NO.17 | HRH4 | Q9H3N8 | Histamine H4 receptor |
| NO.17 | SLC8B1 | Q6J4K2 | Sodium/potassium/calcium exchanger 6, mitochondrial |
| NO.17 | HSD17B3 | P37058 | Estradiol 17-beta-dehydrogenase 3 |
| NO.17 | CCNB3 | Q8WWL7 | G2/mitotic-specific cyclin-B3 |
| NO.17 | CDK1 | P06493 | Cyclin-dependent kinase 1 |
| NO.17 | CCNB1 | P14635 | G2/mitotic-specific cyclin-B1 |
| NO.17 | CCNB2 | O95067 | G2/mitotic-specific cyclin-B2 |
| NO.17 | CYP2C19 | P33261 | Cytochrome P450 2C19 |
| NO.17 | ADORA1 | P30542 | Adenosine A1 receptor |
| NO.17 | ADORA2A | P29274 | Adenosine A2a receptor |
| NO.17 | TMIGD3 | P0DMS9 | Transmembrane domain-containing protein TMIGD3 |
| NO.17 | NAAA | Q02083 | N-acylsphingosine-amidohydrolase (by homology) |
| NO.17 | CCNE1 | P24864 | G1/S-specific cyclin-E1 |
| NO.17 | CDK2 | P24941 | Cyclin-dependent kinase 2 |
| NO.17 | PIK3CD | O00329 | Phosphatidylinositol 4,5-bisphosphate 3-kinase catalytic subunit delta isoform |
| NO.17 | PIK3R1 | P27986 | Phosphatidylinositol 3-kinase regulatory subunit alpha |
| NO.17 | PTPRC | P08575 | Leukocyte common antigen |
| NO.17 | CDK5 | Q00535 | Cyclin-dependent kinase 5/CDK5 activator 1 |
| NO.17 | ALDH2 | P05091 | Aldehyde dehydrogenase |
| NO.17 | HPRT1 | P00492 | Hypoxanthine-guanine phosphoribosyltransferase |
| NO.17 | GRM5 | P41594 | Metabotropic glutamate receptor 5 (by homology) |
| NO.17 | ALDH1A1 | P00352 | Aldehyde dehydrogenase 1A1 |
| NO.17 | ALDH3A1 | P30838 | Aldehyde dehydrogenase dimeric NADP-preferring |
| NO.17 | ABCC9 | O60706 | Sulfonylurea receptor 2 |
| NO.17 | KCNJ11 | Q14654 | ATP-sensitive inward rectifier potassium channel 11 |
| NO.17 | ELANE | P08246 | Leukocyte elastase |
| NO.17 | PRKDC | P78527 | DNA-dependent protein kinase |
| NO.17 | UTS2R | Q9UKP6 | Urotensin II receptor |
| NO.17 | TSPO | P30536 | Translocator protein |
| NO.17 | FADS1 | O60427 | Fatty acid desaturase 1 |
| NO.17 | IDH1 | O75874 | Isocitrate dehydrogenase [NADP] cytoplasmic |
| NO.17 | PIM1 | P11309 | Serine/threonine-protein kinase PIM1 |
| NO.17 | KDR | P35968 | Vascular endothelial growth factor receptor 2 |
| NO.17 | PLAU | P00749 | Urokinase-type plasminogen activator |
| NO.17 | C1R | P00736 | Complement C1r |
| NO.17 | AURKA | O14965 | Serine/threonine-protein kinase Aurora-A |
| NO.17 | BACE1 | P56817 | Beta-secretase 1 |
| NO.17 | CDK5R1 | Q15078 | Cyclin-dependent kinase 5 activator 1 |
| NO.17 | TUBB1 | Q9H4B7 | Tubulin beta-1 chain |
| NO.17 | RAPGEF4 | Q8WZA2 | Rap guanine nucleotide exchange factor 4 |
| NO.17 | CYP2A6 | P11509 | Cytochrome P450 2A6 |
| NO.17 | ERBB2 | P04626 | Receptor protein-tyrosine kinase erbB-2 |
| NO.17 | ADRA2A | P08913 | Alpha-2a adrenergic receptor |
| NO.17 | LIPE | Q05469 | Hormone sensitive lipase |
| NO.17 | HTT | P42858 | Huntingtin |
| NO.17 | IKBKB | O14920 | Inhibitor of nuclear factor kappa B kinase beta subunit |
| NO.17 | P2RX7 | Q99572 | P2X purinoceptor 7 (by homology) |
| NO.17 | TBXA2R | P21731 | Thromboxane A2 receptor |
| NO.17 | PGGT1B | P53609 | Geranylgeranyl transferase type-1 subunit beta |
| NO.17 | FNTA | P49354 | Protein farnesyltransferase/geranylgeranyltransferase type-1 subunit alpha |
| NO.17 | GABRB2 | P47870 | Gamma-aminobutyric acid receptor subunit beta-2 |
| NO.17 | SQLE | Q14534 | Squalene monooxygenase |
| NO.17 | GGPS1 | O95749 | Geranylgeranyl pyrophosphate synthase |
| NO.17 | LSS | P48449 | Lanosterol synthase |
| NO.17 | FNTB | P49356 | Protein farnesyltransferase subunit beta |
| NO.17 | CDC25C | P30307 | M-phase inducer phosphatase 3 |
| NO.18 | CHRM3 | [P20309](https://www.uniprot.org/uniprot/P20309) | Muscarinic acetylcholine receptor M3 |
| NO.18 | CHRM1 | [P11229](https://www.uniprot.org/uniprot/P11229) | Muscarinic acetylcholine receptor M1 |
| NO.18 | PTGS2 | P35354 | Prostaglandin G/H synthase 2 |
| NO.18 | GABRA2 | P47869 | Gamma-aminobutyric acid receptor subunit alpha-2 |
| NO.18 | RXRA | [P19793](https://www.uniprot.org/uniprot/P19793) | Retinoic acid receptor RXR-alpha |
| NO.18 | GABRA6 | Q16445 | Gamma-aminobutyric-acid receptor subunit alpha-6 |
| NO.18 | ADRA1B | [P35368](https://www.uniprot.org/uniprot/P35368) | Alpha-1B adrenergic receptor |
| NO.18 | GABRA1 | [P14867](https://www.uniprot.org/uniprot/P14867) | Gamma-aminobutyric acid receptor subunit alpha-1 |
| NO.18 | PPARA | Q07869 | Peroxisome proliferator-activated receptor alpha |
| NO.18 | CNR2 | P34972 | Cannabinoid receptor 2 |
| NO.18 | AR | P10275 | Androgen Receptor (by homology) |
| NO.18 | CYP19A1 | P11511 | Cytochrome P450 19A1 |
| NO.18 | ESR1 | P03372 | Estrogen receptor alpha |
| NO.18 | CHRM2 | P08172 | Muscarinic acetylcholine receptor M2 |
| NO.18 | ACHE | P22303 | Acetylcholinesterase |
| NO.18 | CYP2C19 | P33261 | Cytochrome P450 2C19 |
| NO.18 | BCHE | P06276 | Butyrylcholinesterase |
| NO.18 | PTPN1 | P18031 | Protein-tyrosine phosphatase 1B |
| NO.18 | NR1I3 | Q14994 | Nuclear receptor subfamily 1 group I member 3 (by homology) |
| NO.18 | FAAH | O00519 | Anandamide amidohydrolase |
| NO.18 | TRPV1 | Q8NER1 | Vanilloid receptor |
| NO.19 | ACHE | P22303 | Acetylcholinesterase |
| NO.19 | NPY5R | Q15761 | Neuropeptide Y receptor type 5 |
| NO.19 | HSD11B1 | P28845 | 11-beta-hydroxysteroid dehydrogenase 1 |
| NO.19 | PTGS1 | P23219 | Cyclooxygenase-1 |
| NO.19 | EPHX1 | P07099 | Epoxide hydrolase 1 |
| NO.19 | CYP17A1 | P05093 | Cytochrome P450 17A1 |
| NO.19 | PTGS2 | P35354 | Cyclooxygenase-2 |
| NO.19 | F2R | P25116 | Proteinase-activated receptor 1 |
| NO.19 | SQLE | Q14534 | Squalene monooxygenase |
| NO.19 | GGPS1 | O95749 | Geranylgeranyl pyrophosphate synthase |
| NO.19 | LSS | P48449 | Lanosterol synthase |
| NO.19 | FNTB | P49356 | Protein farnesyltransferase subunit beta |
| NO.19 | CDC25C | P30307 | M-phase inducer phosphatase 3 |
| NO.19 | FNTA | P49354 | Protein farnesyltransferase/geranylgeranyltransferase type-1 subunit alpha |
| NO.19 | ICMT | O60725 | Protein-S-isoprenylcysteine O-methyltransferase |
| NO.20 | CXCR3 | P49682 | C-X-C chemokine receptor type 3 |
| NO.20 | HTR2A | P28223 | Serotonin 2a (5-HT2a) receptor |
| NO.20 | PTGS2 | P35354 | Prostaglandin G/H synthase 2 |
| NO.20 | GABRA2 | P47869 | Gamma-aminobutyric acid receptor subunit alpha-2 |
| NO.20 | RXRA | [P19793](https://www.uniprot.org/uniprot/P19793) | Retinoic acid receptor RXR-alpha |
| NO.20 | SLC6A2 | P23975 | Sodium-dependent noradrenaline transporter |
| NO.20 | GABRA3 | P34903 | Gamma-aminobutyric-acid receptor alpha-3 subunit |
| NO.20 | CHRM2 | P08172 | Muscarinic acetylcholine receptor M2 |
| NO.20 | GABRA1 | [P14867](https://www.uniprot.org/uniprot/P14867) | Gamma-aminobutyric acid receptor subunit alpha-1 |
| NO.20 | GABRA6 | Q16445 | Gamma-aminobutyric-acid receptor subunit alpha-6 |
| NO.20 | PTGS1 | P23219 | Prostaglandin G/H synthase 1 |
| NO.20 | CHRM3 | [P20309](https://www.uniprot.org/uniprot/P20309) | Muscarinic acetylcholine receptor M3 |
| NO.20 | CHRM1 | [P11229](https://www.uniprot.org/uniprot/P11229) | Muscarinic acetylcholine receptor M1 |
| NO.20 | ADRA1A | P35348 | Alpha-1A adrenergic receptor |
| NO.20 | CHRNA7 | P36544 | Neuronal acetylcholine receptor protein, alpha-7 chain |
| NO.20 | NCOA2 | Q15596 | Nuclear receptor coactivator 2 |
| NO.20 | GABRA5 | P31644 | Gamma-aminobutyric-acid receptor alpha-5 subunit |
| NO.20 | BCL2 | P10415 | Apoptosis regulator Bcl-2 |
| NO.20 | CDKN1A | P38936 | Cyclin-dependent kinase inhibitor 1 |
| NO.20 | EIF6 | P56537 | Eukaryotic translation initiation factor 6 |
| NO.20 | RB1 | P06400 | Retinoblastoma-associated protein |
| NO.20 | TP53 | P04637 | Cellular tumor antigen p53 |
| NO.20 | TEP1 | Q99973 | Telomerase protein component 1 |
| NO.20 | RUNX1T1 | Q06455 | Protein CBFA2T1 |
| NO.20 | CDK1 | P06493 | Cell division control protein 2 homolog |
| NO.20 | CCNB1 | P14635 | G2/mitotic-specific cyclin-B1 |
| NO.20 | RHOA | P61586 | Transforming protein RhoA |
| NO.21 | PTGS1 | P23219 | Prostaglandin G/H synthase 1 |
| NO.21 | CHRM3 | [P20309](https://www.uniprot.org/uniprot/P20309) | Muscarinic acetylcholine receptor M3 |
| NO.21 | CHRM1 | [P11229](https://www.uniprot.org/uniprot/P11229) | Muscarinic acetylcholine receptor M1 |
| NO.21 | PTGS2 | P35354 | Prostaglandin G/H synthase 2 |
| NO.21 | GABRA2 | P47869 | Gamma-aminobutyric acid receptor subunit alpha-2 |
| NO.21 | SLC6A2 | P23975 | Sodium-dependent noradrenaline transporter |
| NO.21 | ADRA1A | P35348 | Alpha-1A adrenergic receptor |
| NO.21 | CHRM2 | P08172 | Muscarinic acetylcholine receptor M2 |
| NO.21 | ADRA1B | P35368 | Alpha-1B adrenergic receptor |
| NO.21 | GABRA1 | [P14867](https://www.uniprot.org/uniprot/P14867) | Gamma-aminobutyric acid receptor subunit alpha-1 |
| NO.21 | NCOA2 | Q15596 | Nuclear receptor coactivator 2 |
| NO.21 | GABRA6 | Q16445 | Gamma-aminobutyric-acid receptor subunit alpha-6 |
| NO.21 | PPARA | Q07869 | Peroxisome proliferator-activated receptor alpha |
| NO.21 | CNR2 | P34972 | Cannabinoid receptor 2 |
| NO.21 | FAAH | O00519 | Anandamide amidohydrolase |
| NO.21 | TRPV1 | Q8NER1 | Vanilloid receptor |
| NO.21 | GLI2 | P10070 | Zinc finger protein GLI2 |
| NO.21 | GLI1 | P08151 | Zinc finger protein GLI1 |
| NO.22 | PTGS1 | P23219 | Prostaglandin G/H synthase 1 |
| NO.22 | CHRM3 | [P20309](https://www.uniprot.org/uniprot/P20309) | Muscarinic acetylcholine receptor M3 |
| NO.22 | CHRM1 | [P11229](https://www.uniprot.org/uniprot/P11229) | Muscarinic acetylcholine receptor M1 |
| NO.22 | PTGS2 | P35354 | Prostaglandin G/H synthase 2 |
| NO.22 | GABRA2 | P47869 | Gamma-aminobutyric acid receptor subunit alpha-2 |
| NO.22 | RXRA | [P19793](https://www.uniprot.org/uniprot/P19793) | Retinoic acid receptor RXR-alpha |
| NO.22 | CHRM2 | P08172 | Muscarinic acetylcholine receptor M2 |
| NO.22 | ADRA1B | P35368 | Alpha-1B adrenergic receptor |
| NO.22 | CHRNA2 | Q15822 | Neuronal acetylcholine receptor subunit alpha-2 |
| NO.22 | GABRA1 | [P14867](https://www.uniprot.org/uniprot/P14867) | Gamma-aminobutyric acid receptor subunit alpha-1 |
| NO.22 | NCOA2 | Q15596 | Nuclear receptor coactivator 2 |
| NO.22 | GABRA6 | Q16445 | Gamma-aminobutyric-acid receptor subunit alpha-6 |
| NO.22 | SLC6A2 | P23975 | Sodium-dependent noradrenaline transporter |
| NO.22 | ADRA1A | P35348 | Alpha-1A adrenergic receptor |
| NO.22 | SLC6A3 | Q01959 | Sodium-dependent dopamine transporter |
| NO.22 | IL6 | P05231 | Interleukin-6 |
| NO.22 | PPARA | Q07869 | Peroxisome proliferator-activated receptor alpha |
| NO.22 | CNR2 | P34972 | Cannabinoid receptor 2 |
| NO.22 | FAAH | O00519 | Anandamide amidohydrolase |
| NO.22 | TRPV1 | Q8NER1 | Vanilloid receptor |
| NO.22 | GLI2 | P10070 | Zinc finger protein GLI2 |
| NO.22 | GLI1 | P08151 | Zinc finger protein GLI1 |
| NO.23 | PPARA | Q07869 | Peroxisome proliferator-activated receptor alpha |
| NO.23 | CNR2 | P34972 | Cannabinoid receptor 2 |
| NO.24 | PTGS1 | P23219 | Prostaglandin G/H synthase 1 |
| NO.24 | CHRM3 | [P20309](https://www.uniprot.org/uniprot/P20309) | Muscarinic acetylcholine receptor M3 |
| NO.24 | PTGS2 | P35354 | Prostaglandin G/H synthase 2 |
| NO.24 | GABRA2 | P47869 | Gamma-aminobutyric acid receptor subunit alpha-2 |
| NO.24 | RXRA | [P19793](https://www.uniprot.org/uniprot/P19793) | Retinoic acid receptor RXR-alpha |
| NO.24 | SLC6A2 | P23975 | Sodium-dependent noradrenaline transporter |
| NO.24 | ADRA1A | P35348 | Alpha-1A adrenergic receptor |
| NO.24 | GABRA3 | P34903 | Gamma-aminobutyric-acid receptor alpha-3 subunit |
| NO.24 | CHRM2 | P08172 | Muscarinic acetylcholine receptor M2 |
| NO.24 | GABRA1 | [P14867](https://www.uniprot.org/uniprot/P14867) | Gamma-aminobutyric acid receptor subunit alpha-1 |
| NO.24 | GABRA6 | Q16445 | Gamma-aminobutyric-acid receptor subunit alpha-6 |
| NO.24 | PPARA | Q07869 | Peroxisome proliferator-activated receptor alpha |
| NO.24 | CNR2 | P34972 | Cannabinoid receptor 2 |
| NO.24 | CXCR3 | P49682 | C-X-C chemokine receptor type 3 |
| NO.24 | NR1H3 | Q13133 | LXR-alpha |
| NO.26 | PTPN2 | P17706 | T-cell protein-tyrosine phosphatase |
| NO.26 | MAOB | P27338 | Monoamine oxidase B |
| NO.26 | AR | P10275 | Androgen Receptor |
| NO.26 | ESR1 | P03372 | Estrogen receptor alpha |
| NO.26 | ESR2 | Q92731 | Estrogen receptor beta |
| NO.26 | CYP19A1 | P11511 | Cytochrome P450 19A1 |
| NO.26 | CHRM2 | P08172 | Muscarinic acetylcholine receptor M2 |
| NO.26 | ACHE | P22303 | Acetylcholinesterase |
| NO.26 | SLC6A2 | P23975 | Norepinephrine transporter |
| NO.26 | SLC6A4 | P31645 | Serotonin transporter |
| NO.26 | CYP2C19 | P33261 | Cytochrome P450 2C19 |
| NO.26 | TRPV1 | Q8NER1 | Vanilloid receptor |
| NO.27 | ACHE | P22303 | Acetylcholinesterase |
| NO.27 | ADH1A | P07327 | Alcohol dehydrogenase alpha chain |
| NO.27 | ADH1B | P00325 | Alcohol dehydrogenase beta chain |
| NO.27 | ADH1C | P00326 | Alcohol dehydrogenase gamma chain |
| NO.27 | ADORA1 | P30542 | Adenosine A1 receptor (by homology) |
| NO.27 | ADORA2A | P29274 | Adenosine A2a receptor |
| NO.27 | ADORA2B | P29275 | Adenosine A2b receptor |
| NO.27 | ADORA3 | P0DMS8 | Adenosine A3 receptor |
| NO.27 | AKT1 | P31749 | Serine/threonine-protein kinase AKT |
| NO.27 | ALOX12 | P18054 | Arachidonate 12-lipoxygenase |
| NO.27 | ALOX15 | P16050 | Arachidonate 15-lipoxygenase |
| NO.27 | AVPR1A | P37288 | Vasopressin V1a receptor |
| NO.27 | AVPR2 | P30518 | Vasopressin V2 receptor |
| NO.27 | BRD4 | O60885 | Bromodomain-containing protein 4 |
| NO.27 | CA1 | P00915 | Carbonic anhydrase I |
| NO.27 | CA12 | O43570 | Carbonic anhydrase XII |
| NO.27 | CA2 | P00918 | Carbonic anhydrase II |
| NO.27 | CA7 | P43166 | Carbonic anhydrase VII |
| NO.27 | CA9 | Q16790 | Carbonic anhydrase IX |
| NO.27 | CACNA1B | Q00975 | Voltage-gated N-type calcium channel alpha-1B subunit |
| NO.27 | CCR2 | P41597 | C-C chemokine receptor type 2 |
| NO.27 | CHRM1 | P11229 | Muscarinic acetylcholine receptor M1 (by homology) |
| NO.27 | CHRM2 | P08172 | Muscarinic acetylcholine receptor M2 (by homology) |
| NO.27 | CHRM3 | P20309 | Muscarinic acetylcholine receptor M3 |
| NO.27 | CHRNA7 | P36544 | Neuronal acetylcholine receptor protein alpha-7 subunit |
| NO.27 | CNR2 | P34972 | Cannabinoid receptor 2 |
| NO.27 | CTSB | P07858 | Cathepsin (B and K) |
| NO.27 | CTSK | P43235 | Cathepsin K |
| NO.27 | CTSL | P07711 | Cathepsin L |
| NO.27 | CTSS | P25774 | Cathepsin S |
| NO.27 | CTSV | O60911 | Cathepsin (V and K) |
| NO.27 | CYP11B1 | P15538 | Cytochrome P450 11B1 |
| NO.27 | CYP11B2 | P19099 | Cytochrome P450 11B2 |
| NO.27 | CYP24A1 | Q07973 | Cytochrome P450 24A1 |
| NO.27 | CYP27A1 | Q02318 | Sterol 26-hydroxylase, mitochondrial |
| NO.27 | CYP2C19 | P33261 | Cytochrome P450 2C19 |
| NO.27 | DBF4 | Q9UBU7 | Activator of S phase kinase |
| NO.27 | CDC7 | O00311 | Cell division cycle 7-related protein kinase |
| NO.27 | DRD2 | P14416 | Dopamine D2 receptor |
| NO.27 | DUSP3 | P51452 | Dual specificity protein phosphatase 3 |
| NO.27 | EEF2K | O00418 | Serine/threonine-protein kinase EEF2K |
| NO.27 | EPHX1 | P07099 | Epoxide hydrolase 1 |
| NO.27 | EPHX2 | P34913 | Epoxide hydratase |
| NO.27 | FAAH | O00519 | Anandamide amidohydrolase |
| NO.27 | FKBP1A | P62942 | FK506-binding protein 1A |
| NO.27 | GABRA1 | P14867 | Gamma-aminobutyric acid receptor subunit alpha-1 |
| NO.27 | GABRB2 | P47870 | Gamma-aminobutyric acid receptor subunit beta-2 |
| NO.27 | GABRG2 | P18507 | Gamma-aminobutyric acid receptor subunit gamma-2 |
| NO.27 | GABRA2 | P47869 | Gamma-aminobutyric acid receptor subunit alpha-2 |
| NO.27 | GABRB3 | P28472 | Gamma-aminobutyric acid receptor subunit beta-3 |
| NO.27 | GABRA3 | P34903 | Gamma-aminobutyric acid receptor subunit alpha-3 |
| NO.27 | GABRA5 | P31644 | Gamma-aminobutyric acid receptor subunit alpha-5 |
| NO.27 | GABRA6 | Q16445 | Gamma-aminobutyric acid receptor subunit alpha-6 |
| NO.27 | GRK2 | P25098 | G-protein coupled receptor kinase 2 |
| NO.27 | GRM1 | Q13255 | Metabotropic glutamate receptor 1 |
| NO.27 | GRM2 | Q14416 | Metabotropic glutamate receptor 2 |
| NO.27 | GRM5 | P41594 | Metabotropic glutamate receptor 5 (by homology) |
| NO.27 | HCRTR1 | O43613 | Orexin receptor 1 |
| NO.27 | HCRTR2 | O43614 | Orexin receptor 2 |
| NO.27 | HLCS | P50747 | Biotin--protein ligase |
| NO.27 | HSP90AA1 | P07900 | Heat shock protein HSP 90-alpha |
| NO.27 | JAK1 | P23458 | Tyrosine-protein kinase JAK1 |
| NO.27 | JAK2 | O60674 | Tyrosine-protein kinase JAK2 |
| NO.27 | KCNA5 | P22460 | Voltage-gated potassium channel subunit Kv1.5 |
| NO.27 | LRRK2 | Q5S007 | Leucine-rich repeat serine/threonine-protein kinase 2 |
| NO.27 | MAOA | P21397 | Monoamine oxidase A |
| NO.27 | MAOB | P27338 | Monoamine oxidase B |
| NO.27 | MAPK14 | Q16539 | MAP kinase p38 alpha |
| NO.27 | MMP1 | P03956 | Matrix metalloproteinase 1 |
| NO.27 | MMP13 | P45452 | Matrix metalloproteinase 13 |
| NO.27 | MMP3 | P08254 | Matrix metalloproteinase 3 |
| NO.27 | MMP8 | P22894 | Matrix metalloproteinase 8 |
| NO.27 | MMP9 | P14780 | Matrix metalloproteinase 9 |
| NO.27 | MTNR1A | P48039 | Melatonin receptor 1A |
| NO.27 | MTNR1B | P49286 | Melatonin receptor 1B |
| NO.27 | NAAA | Q02083 | N-acylsphingosine-amidohydrolase |
| NO.27 | NMT1 | P30419 | Peptide N-myristoyltransferase 1 |
| NO.27 | NOS1 | P29475 | Nitric-oxide synthase, brain |
| NO.27 | NOS3 | P29474 | Nitric-oxide synthase, endothelial |
| NO.27 | NQO2 | P16083 | Quinone reductase 2 |
| NO.27 | P2RX7 | Q99572 | P2X purinoceptor 7 |
| NO.27 | PARP1 | P09874 | Poly [ADP-ribose] polymerase-1 |
| NO.27 | PDE5A | O76074 | Phosphodiesterase 5A |
| NO.27 | PDE9A | O76083 | Phosphodiesterase 9A |
| NO.27 | PSEN2 | P49810 | Presenilin-2 |
| NO.27 | PSENEN | Q9NZ42 | Gamma-secretase subunit PEN-2 |
| NO.27 | NCSTN | Q92542 | Nicastrin |
| NO.27 | APH1A | Q96BI3 | Gamma-secretase subunit APH-1A |
| NO.27 | PSEN1 | P49768 | Presenilin-1 |
| NO.27 | APH1B | Q8WW43 | Gamma-secretase subunit APH-1B |
| NO.27 | PSMB5 | P28074 | Proteasome Macropain subunit MB1 |
| NO.27 | S1PR1 | P21453 | Sphingosine 1-phosphate receptor Edg-1 |
| NO.27 | S1PR3 | Q99500 | Sphingosine 1-phosphate receptor Edg-3 |
| NO.27 | SIRT1 | Q96EB6 | NAD-dependent deacetylase sirtuin 1 |
| NO.27 | SIRT2 | Q8IXJ6 | NAD-dependent deacetylase sirtuin 2 |
| NO.27 | SLC18A3 | Q16572 | Vesicular acetylcholine transporter |
| NO.27 | SLC6A2 | P23975 | Norepinephrine transporter |
| NO.27 | SLC6A3 | Q01959 | Dopamine transporter |
| NO.27 | SLC6A4 | P31645 | Serotonin transporter |
| NO.27 | SPHK2 | Q9NRA0 | Sphingosine kinase 2 |
| NO.27 | TGM2 | P21980 | Protein-glutamine gamma-glutamyltransferase |
| NO.27 | THRB | P10828 | Thyroid hormone receptor beta-1 |
| NO.27 | TNKS | O95271 | Tankyrase-1 |
| NO.27 | TNKS2 | Q9H2K2 | Tankyrase-2 |
| NO.27 | TRPM8 | Q7Z2W7 | Transient receptor potential cation channel subfamily M member 8 (by homology) |
| NO.27 | TRPV1 | Q8NER1 | Vanilloid receptor |
| NO.27 | TSPO | P30536 | Translocator protein (by homology) |
| NO.27 | SQLE | Q14534 | Squalene monooxygenase |
| NO.27 | GGPS1 | O95749 | Geranylgeranyl pyrophosphate synthase |
| NO.27 | LSS | P48449 | Lanosterol synthase |
| NO.27 | FNTB | P49356 | Protein farnesyltransferase subunit beta |
| NO.27 | CDC25C | P30307 | M-phase inducer phosphatase 3 |
| NO.27 | FNTA | P49354 | Protein farnesyltransferase/geranylgeranyltransferase type-1 subunit alpha |
| NO.27 | ICMT | O60725 | Protein-S-isoprenylcysteine O-methyltransferase |
| NO.28 | PTGS1 | P23219 | Prostaglandin G/H synthase 1 |
| NO.28 | CHRM3 | [P20309](https://www.uniprot.org/uniprot/P20309) | Muscarinic acetylcholine receptor M3 |
| NO.28 | CHRM1 | [P11229](https://www.uniprot.org/uniprot/P11229) | Muscarinic acetylcholine receptor M1 |
| NO.28 | PTGS2 | P35354 | Prostaglandin G/H synthase 2 |
| NO.28 | GABRA2 | P47869 | Gamma-aminobutyric acid receptor subunit alpha-2 |
| NO.28 | CHRM2 | P08172 | Muscarinic acetylcholine receptor M2 |
| NO.28 | ADRA1B | P35368 | Alpha-1B adrenergic receptor |
| NO.28 | CHRNA2 | Q15822 | Neuronal acetylcholine receptor subunit alpha-2 |
| NO.28 | GABRA1 | [P14867](https://www.uniprot.org/uniprot/P14867) | Gamma-aminobutyric acid receptor subunit alpha-1 |
| NO.28 | NCOA2 | Q15596 | Nuclear receptor coactivator 2 |
| NO.28 | GABRA6 | Q16445 | Gamma-aminobutyric-acid receptor subunit alpha-6 |
| NO.28 | SLC6A2 | P23975 | Sodium-dependent noradrenaline transporter |
| NO.28 | ADRA1A | P35348 | Alpha-1A adrenergic receptor |
| NO.28 | SLC6A3 | Q01959 | Sodium-dependent dopamine transporter |
| NO.28 | ADRB2 | P07550 | Beta-2 adrenergic receptor |
| NO.28 | PPARA | Q07869 | Peroxisome proliferator-activated receptor alpha |
| NO.28 | CNR2 | P34972 | Cannabinoid receptor 2 |
| NO.28 | FAAH | O00519 | Anandamide amidohydrolase |
| NO.28 | TRPV1 | Q8NER1 | Vanilloid receptor |
| NO.28 | GLI2 | P10070 | Zinc finger protein GLI2 |
| NO.28 | GLI1 | P08151 | Zinc finger protein GLI1 |
| NO.29 | PTGS1 | P23219 | Prostaglandin G/H synthase 1 |
| NO.29 | CHRM1 | [P11229](https://www.uniprot.org/uniprot/P11229) | Muscarinic acetylcholine receptor M1 |
| NO.29 | PTGS2 | P35354 | Prostaglandin G/H synthase 2 |
| NO.29 | RXRA | P19793 | Retinoic acid receptor RXR-alpha |
| NO.29 | ADRA1B | P35368 | Alpha-1B adrenergic receptor |
| NO.29 | GABRA1 | [P14867](https://www.uniprot.org/uniprot/P14867) | Gamma-aminobutyric acid receptor subunit alpha-1 |
| NO.29 | PPARA | Q07869 | Peroxisome proliferator-activated receptor alpha |
| NO.29 | CNR2 | P34972 | Cannabinoid receptor 2 |
| NO.29 | NR1H3 | Q13133 | LXR-alpha |
| NO.29 | CYP19A1 | P11511 | Cytochrome P450 19A1 |
| NO.29 | AR | P10275 | Androgen Receptor (by homology) |
| NO.29 | ESR1 | P03372 | Estrogen receptor alpha |
| NO.29 | CHRM2 | P08172 | Muscarinic acetylcholine receptor M2 |
| NO.29 | ACHE | P22303 | Acetylcholinesterase |
| NO.29 | SLC6A2 | P23975 | Norepinephrine transporter |
| NO.29 | SLC6A4 | P31645 | Serotonin transporter |
| NO.29 | CYP2C19 | P33261 | Cytochrome P450 2C19 |
| NO.30 | PTGS1 | P23219 | Prostaglandin G/H synthase 1 |
| NO.30 | PTGS2 | P35354 | Prostaglandin G/H synthase 2 |
| NO.30 | SLC6A2 | P23975 | Sodium-dependent noradrenaline transporter |
| NO.30 | NCOA2 | Q15596 | Nuclear receptor coactivator 2 |
| NO.30 | PPARA | Q07869 | Peroxisome proliferator-activated receptor alpha |
| NO.30 | CNR2 | P34972 | Cannabinoid receptor 2 |
| NO.30 | ADORA1 | P30542 | Adenosine A1 receptor |
| NO.30 | ADORA2A | P29274 | Adenosine A2a receptor |
| NO.30 | ADORA3 | P0DMS8 | Adenosine A3 receptor |
| NO.30 | AR | P10275 | Androgen Receptor |
| NO.30 | CYP19A1 | P11511 | Cytochrome P450 19A1 |
| NO.30 | ESR1 | P03372 | Estrogen receptor alpha |
| NO.30 | ESR2 | Q92731 | Estrogen receptor beta |
| NO.31 | RBP4 | P02753 | Retinol-binding protein 4 |
| NO.31 | CYP19A1 | P11511 | Cytochrome P450 19A1 |
| NO.31 | AR | P10275 | Androgen Receptor |
| NO.31 | RORC | P51449 | Nuclear receptor ROR-gamma |
| NO.31 | RXRB | P28702 | Retinoid X receptor beta |
| NO.31 | ADRA2B | P18089 | Alpha-2b adrenergic receptor |
| NO.31 | RARG | P13631 | Retinoic acid receptor gamma |
| NO.31 | RXRG | P48443 | Retinoid X receptor gamma |
| NO.31 | RARB | P10826 | Retinoic acid receptor beta |
| NO.31 | RARA | P10276 | Retinoic acid receptor alpha |
| NO.31 | RXRA | P19793 | Retinoid X receptor alpha |
| NO.31 | RORB | Q92753 | Nuclear receptor ROR-beta |
| NO.31 | MAPK1 | P28482 | MAP kinase ERK2 |
| NO.31 | RORA | P35398 | Nuclear receptor ROR-alpha |
| NO.31 | ADH1A | P07327 | Alcohol dehydrogenase alpha chain |
| NO.31 | SRD5A1 | P18405 | Steroid 5-alpha-reductase 1 |
| NO.31 | PGR | P06401 | Progesterone receptor |
| NO.31 | ACHE | P22303 | Acetylcholinesterase |
| NO.31 | TYMS | P04818 | Thymidylate synthase |
| NO.31 | SRD5A2 | P31213 | Steroid 5-alpha-reductase 2 |
| NO.31 | MPI | P34949 | Mannose-6-phosphate isomerase |
| NO.31 | CES2 | O00748 | Carboxylesterase 2 |
| NO.31 | MPO | P05164 | Myeloperoxidase |
| NO.31 | HTR7 | P34969 | Serotonin 7 (5-HT7) receptor |
| NO.31 | ADH1C | P00326 | Alcohol dehydrogenase gamma chain |
| NO.31 | CHRM1 | P11229 | Muscarinic acetylcholine receptor M1 |
| NO.31 | PTPN1 | P18031 | Protein-tyrosine phosphatase 1B |
| NO.31 | CHRNA4 | P43681 | Neuronal acetylcholine receptor subunit alpha-4 |
| NO.31 | CHRNB2 | P17787 | Neuronal acetylcholine receptor subunit beta-2 |
| NO.31 | P2RX7 | Q99572 | P2X purinoceptor 7 |
| NO.31 | BAX | Q07812 | Apoptosis regulator BAX |
| NO.31 | RPE65 | Q16518 | Retinoid isomerohydrolase |
| NO.31 | BCO2 | Q9BYV7 | Beta,beta-carotene 9',10'-oxygenase |
| NO.31 | RHO | P08100 | Rhodopsin |
| NO.32 | PTGS1 | P23219 | Prostaglandin G/H synthase 1 |
| NO.32 | PTGS2 | P35354 | Prostaglandin G/H synthase 2 |
| NO.32 | GABRA2 | P47869 | Gamma-aminobutyric acid receptor subunit alpha-2 |
| NO.32 | CHRM2 | P08172 | Muscarinic acetylcholine receptor M2 |
| NO.32 | ADRA1B | P35368 | Alpha-1B adrenergic receptor |
| NO.32 | GABRA1 | [P14867](https://www.uniprot.org/uniprot/P14867) | Gamma-aminobutyric acid receptor subunit alpha-1 |
| NO.32 | NCOA2 | Q15596 | Nuclear receptor coactivator 2 |
| NO.32 | GABRA6 | Q16445 | Gamma-aminobutyric-acid receptor subunit alpha-6 |
| NO.32 | PPARA | Q07869 | Peroxisome proliferator-activated receptor alpha |
| NO.32 | CNR2 | P34972 | Cannabinoid receptor 2 |
| NO.32 | FAAH | O00519 | Anandamide amidohydrolase |
| NO.32 | TRPV1 | Q8NER1 | Vanilloid receptor |
| NO.32 | GLI2 | P10070 | Zinc finger protein GLI2 |
| NO.32 | GLI1 | P08151 | Zinc finger protein GLI1 |
| NO.32 | GPR18 | Q14330 | N-arachidonyl glycine receptor |
| NO.33 | SQLE | Q14534 | Squalene monooxygenase |
| NO.33 | GGPS1 | O95749 | Geranylgeranyl pyrophosphate synthase |
| NO.33 | LSS | P48449 | Lanosterol synthase |
| NO.33 | FNTB | P49356 | Protein farnesyltransferase subunit beta |
| NO.33 | FNTA | P49354 | Protein farnesyltransferase/geranylgeranyltransferase type-1 subunit alpha |
| NO.34 | PTGS1 | P23219 | Prostaglandin G/H synthase 1 |
| NO.34 | CHRM3 | [P20309](https://www.uniprot.org/uniprot/P20309) | Muscarinic acetylcholine receptor M3 |
| NO.34 | CHRM1 | [P11229](https://www.uniprot.org/uniprot/P11229) | Muscarinic acetylcholine receptor M1 |
| NO.34 | PTGS2 | P35354 | Prostaglandin G/H synthase 2 |
| NO.34 | RXRA | P19793 | Retinoic acid receptor RXR-alpha |
| NO.34 | ADRA1B | P35368 | Alpha-1B adrenergic receptor |
| NO.34 | GABRA1 | [P14867](https://www.uniprot.org/uniprot/P14867) | Gamma-aminobutyric acid receptor subunit alpha-1 |
| NO.34 | NCOA2 | Q15596 | Nuclear receptor coactivator 2 |
| NO.34 | GABRA6 | Q16445 | Gamma-aminobutyric-acid receptor subunit alpha-6 |
| NO.34 | PPARA | Q07869 | Peroxisome proliferator-activated receptor alpha |
| NO.34 | CNR2 | P34972 | Cannabinoid receptor 2 |
| NO.34 | NR1H3 | Q13133 | LXR-alpha |
| NO.34 | CYP19A1 | P11511 | Cytochrome P450 19A1 |
| NO.34 | AR | P10275 | Androgen Receptor (by homology) |
| NO.34 | ESR1 | P03372 | Estrogen receptor alpha |
| NO.34 | CHRM2 | P08172 | Muscarinic acetylcholine receptor M2 |
| NO.34 | ACHE | P22303 | Acetylcholinesterase |
| NO.34 | SLC6A2 | P23975 | Norepinephrine transporter |
| NO.34 | SLC6A4 | P31645 | Serotonin transporter |
| NO.34 | CYP2C19 | P33261 | Cytochrome P450 2C19 |
| NO.35 | PPARA | Q07869 | Peroxisome proliferator-activated receptor alpha |
| NO.35 | CNR2 | P34972 | Cannabinoid receptor 2 |
| NO.35 | TRPV1 | Q8NER1 | Vanilloid receptor |
| NO.35 | GLI2 | P10070 | Zinc finger protein GLI2 |
| NO.35 | GLI1 | P08151 | Zinc finger protein GLI1 |
| NO.36 | CYP19A1 | P11511 | Cytochrome P450 19A1 |
| NO.36 | PPARA | Q07869 | Peroxisome proliferator-activated receptor alpha |
| NO.36 | CNR2 | P34972 | Cannabinoid receptor 2 |
| NO.36 | TRPV1 | Q8NER1 | Vanilloid receptor |
| NO.36 | PTPN2 | P17706 | T-cell protein-tyrosine phosphatase |
| NO.37 | CHRM3 | [P20309](https://www.uniprot.org/uniprot/P20309) | Muscarinic acetylcholine receptor M3 |
| NO.37 | CHRM1 | [P11229](https://www.uniprot.org/uniprot/P11229) | Muscarinic acetylcholine receptor M1 |
| NO.37 | PTGS2 | P35354 | Prostaglandin G/H synthase 2 |
| NO.37 | ACHE | P22303 | Acetylcholinesterase |
| NO.37 | CHRM2 | P08172 | Muscarinic acetylcholine receptor M2 |
| NO.37 | ADRA1B | P35368 | Alpha-1B adrenergic receptor |
| NO.37 | GABRA1 | [P14867](https://www.uniprot.org/uniprot/P14867) | Gamma-aminobutyric acid receptor subunit alpha-1 |
| NO.37 | DPP4 | P27487 | Dipeptidyl peptidase IV |
| NO.37 | GABRA6 | Q16445 | Gamma-aminobutyric-acid receptor subunit alpha-6 |
| NO.37 | SLC6A3 | Q01959 | Sodium-dependent dopamine transporter |
| NO.37 | SLC6A4 | P31645 | Serotonin transporter |
| NO.37 | SQLE | Q14534 | Squalene monooxygenase |
| NO.37 | LSS | P48449 | Lanosterol synthase |
| NO.38 | PTGS2 | P35354 | Prostaglandin G/H synthase 2 |
| NO.38 | RXRA | P19793 | Retinoic acid receptor RXR-alpha |
| NO.38 | SLC6A2 | P23975 | Sodium-dependent noradrenaline transporter |
| NO.38 | GABRA1 | [P14867](https://www.uniprot.org/uniprot/P14867) | Gamma-aminobutyric acid receptor subunit alpha-1 |
| NO.38 | MAOB | P27338 | Amine oxidase [flavin-containing] B |
| NO.38 | GLI2 | P10070 | Zinc finger protein GLI2 |
| NO.38 | GLI1 | P08151 | Zinc finger protein GLI1 |
| NO.38 | PPARA | Q07869 | Peroxisome proliferator-activated receptor alpha |
| NO.38 | CNR2 | P34972 | Cannabinoid receptor 2 |
| NO.38 | TRPV1 | Q8NER1 | Vanilloid receptor |
| NO.38 | ADORA1 | P30542 | Adenosine A1 receptor |
| NO.38 | ADORA2A | P29274 | Adenosine A2a receptor |
| NO.38 | ADORA3 | P0DMS8 | Adenosine A3 receptor |
| NO.39 | PTGS2 | P35354 | Prostaglandin G/H synthase 2 |
| NO.39 | SLC6A2 | P23975 | Sodium-dependent noradrenaline transporter |
| NO.39 | GABRA1 | [P14867](https://www.uniprot.org/uniprot/P14867) | Gamma-aminobutyric acid receptor subunit alpha-1 |
| NO.39 | SQLE | Q14534 | Squalene monooxygenase |
| NO.39 | IDO1 | P14902 | Indoleamine 2,3-dioxygenase |
| NO.39 | BACE1 | P56817 | Beta-secretase 1 |
| NO.39 | GRM5 | P41594 | Metabotropic glutamate receptor 5 |
| NO.39 | GCGR | P47871 | Glucagon receptor |
| NO.39 | PGR | P06401 | Progesterone receptor |
| NO.39 | PER2 | O15055 | Period circadian protein homolog 2 |
| NO.39 | PSEN2 | P49810 | Presenilin-2 |
| NO.39 | PSENEN | Q9NZ42 | Gamma-secretase subunit PEN-2 |
| NO.39 | NCSTN | Q92542 | Nicastrin |
| NO.39 | APH1A | Q96BI3 | Gamma-secretase subunit APH-1A |
| NO.39 | PSEN1 | P49768 | Presenilin-1 |
| NO.39 | APH1B | Q8WW43 | Gamma-secretase subunit APH-1B |
| NO.39 | SLC10A2 | Q12908 | Ileal bile acid transporter |
| NO.39 | NR3C1 | P04150 | Glucocorticoid receptor |
| NO.39 | KCNH2 | Q12809 | HERG |
| NO.39 | HSD17B2 | P37059 | Estradiol 17-beta-dehydrogenase 2 |
| NO.39 | NR1H3 | Q13133 | LXR-alpha |
| NO.39 | PDE2A | O00408 | Phosphodiesterase 2A |
| NO.39 | PDE10A | Q9Y233 | Phosphodiesterase 10A |
| NO.39 | TTL | Q8NG68 | Tubulin--tyrosine ligase |
| NO.39 | HSD17B3 | P37058 | Estradiol 17-beta-dehydrogenase 3 |
| NO.39 | AVPR1A | P37288 | Vasopressin V1a receptor |
| NO.39 | NPY5R | Q15761 | Neuropeptide Y receptor type 5 |
| NO.39 | MAPK14 | Q16539 | MAP kinase p38 alpha (by homology) |
| NO.39 | AKR1C3 | P42330 | Aldo-keto-reductase family 1 member C3 |
| NO.39 | MAPK8 | P45983 | c-Jun N-terminal kinase 1 |
| NO.39 | SLC6A3 | Q01959 | Dopamine transporter (by homology) |
| NO.39 | PABPC1 | P11940 | Polyadenylate-binding protein 1 |
| NO.39 | IL6ST | P40189 | Interleukin-6 receptor subunit beta |
| NO.39 | CYP2C9 | P11712 | Cytochrome P450 2C9 |
| NO.39 | CYP3A4 | P08684 | Cytochrome P450 3A4 |
| NO.39 | GRM2 | Q14416 | Metabotropic glutamate receptor 2 |
| NO.39 | PRKCD | Q05655 | Protein kinase C delta |
| NO.39 | TRPV1 | Q8NER1 | Vanilloid receptor |
| NO.39 | PRCP | P42785 | Lysosomal Pro-X carboxypeptidase |
| NO.39 | OXTR | P30559 | Oxytocin receptor (by homology) |
| NO.39 | JAK1 | P23458 | Tyrosine-protein kinase JAK1 |
| NO.39 | JAK2 | O60674 | Tyrosine-protein kinase JAK2 |
| NO.39 | CDC25A | P30304 | Dual specificity phosphatase Cdc25A |
| NO.39 | NR1H2 | P55055 | LXR-beta |
| NO.39 | PGGT1B | P53609 | Geranylgeranyl transferase type I beta subunit |
| NO.39 | KCNA5 | P22460 | Voltage-gated potassium channel subunit Kv1.5 |
| NO.39 | KCNA3 | P22001 | Voltage-gated potassium channel subunit Kv1.3 |
| NO.39 | SCN9A | Q15858 | Sodium channel protein type IX alpha subunit |
| NO.39 | CYP11B1 | P15538 | Cytochrome P450 11B1 |
| NO.39 | EPHX2 | P34913 | Epoxide hydratase |
| NO.39 | CYP11B2 | P19099 | Cytochrome P450 11B2 |
| NO.39 | F2R | P25116 | Proteinase-activated receptor 1 |
| NO.39 | CHRM1 | P11229 | Muscarinic acetylcholine receptor M1 |
| NO.39 | CHRM3 | P20309 | Muscarinic acetylcholine receptor M3 |
| NO.39 | TNK2 | Q07912 | Tyrosine kinase non-receptor protein 2 |
| NO.39 | CASR | P41180 | Calcium sensing receptor |
| NO.39 | HRH3 | Q9Y5N1 | Histamine H3 receptor |
| NO.39 | CHRM4 | P08173 | Muscarinic acetylcholine receptor M4 |
| NO.39 | CHRM5 | P08912 | Muscarinic acetylcholine receptor M5 |
| NO.39 | GABBR2 | O75899 | Gamma-aminobutyric acid type B receptor subunit 2 |
| NO.39 | GABBR1 | Q9UBS5 | Gamma-aminobutyric acid type B receptor subunit 1 |
| NO.39 | JAK3 | P52333 | Tyrosine-protein kinase JAK3 |
| NO.39 | ABHD6 | Q9BV23 | Monoacylglycerol lipase ABHD6 |
| NO.39 | PTPN1 | P18031 | Protein-tyrosine phosphatase 1B |
| NO.39 | CCR1 | P32246 | C-C chemokine receptor type 1 |
| NO.39 | CCR9 | P51686 | C-C chemokine receptor type 9 |
| NO.39 | BRS3 | P32247 | Bombesin receptor subtype-3 |
| NO.39 | ICMT | O60725 | Isoprenylcysteine carboxyl methyltransferase |
| NO.39 | LYPLA2 | O95372 | Acyl-protein thioesterase 2 |
| NO.39 | PRKCG | P05129 | Protein kinase C gamma |
| NO.39 | PRKCA | P17252 | Protein kinase C alpha |
| NO.39 | PRKCB | P05771 | Protein kinase C beta |
| NO.39 | NOS1 | P29475 | Nitric-oxide synthase, brain |
| NO.39 | PRKCE | Q02156 | Protein kinase C epsilon |
| NO.39 | PRKCH | P24723 | Protein kinase C eta |
| NO.39 | PRKCQ | Q04759 | Protein kinase C theta |
| NO.39 | NPY2R | P49146 | Neuropeptide Y receptor type 2 |
| NO.39 | HIF1A | Q16665 | Hypoxia-inducible factor 1 alpha |
| NO.39 | RASGRP1 | O95267 | RAS guanyl-releasing protein 1 (by homology) |
| NO.39 | FDFT1 | P37268 | Squalene synthetase |
| NO.39 | GCK | P35557 | Hexokinase type IV |
| NO.39 | EPHX1 | P07099 | Epoxide hydrolase 1 |
| NO.39 | OPRL1 | P41146 | Nociceptin receptor |
| NO.39 | LIPE | Q05469 | Hormone sensitive lipase |
| NO.39 | TRPA1 | O75762 | Transient receptor potential cation channel subfamily A member 1 |
| NO.39 | GLUL | P15104 | Glutamine synthetase |
| NO.39 | THRA | P10827 | Thyroid hormone receptor alpha |
| NO.39 | PYGL | P06737 | Liver glycogen phosphorylase |
| NO.39 | CNR2 | P34972 | Cannabinoid receptor 2 |
| NO.39 | P2RX7 | Q99572 | P2X purinoceptor 7 |
| NO.39 | SIGMAR1 | Q99720 | Sigma opioid receptor |
| NO.39 | C5AR1 | P21730 | C5a anaphylatoxin chemotactic receptor |
| NO.39 | MAP3K8 | P41279 | Mitogen-activated protein kinase kinase kinase 8 |
| NO.39 | LSS | P48449 | Lanosterol synthase |
| NO.39 | GGPS1 | O95749 | Geranylgeranyl pyrophosphate synthase |
| NO.39 | FNTB | P49356 | Protein farnesyltransferase subunit beta |
| NO.39 | FNTA | P49354 | Protein farnesyltransferase/geranylgeranyltransferase type-1 subunit alpha |
| NO.40 | CHRM1 | P11229 | Muscarinic acetylcholine receptor M1 |
| NO.40 | CHRM3 | P20309 | Muscarinic acetylcholine receptor M3 |
| NO.40 | CHRM2 | P08172 | Muscarinic acetylcholine receptor M2 |
| NO.40 | UGT2B7 | P16662 | UDP-glucuronosyltransferase 2B7 |
| NO.40 | HSD11B1 | P28845 | 11-beta-hydroxysteroid dehydrogenase 1 |
| NO.40 | IDO1 | P14902 | Indoleamine 2,3-dioxygenase |
| NO.40 | PTGS1 | P23219 | Cyclooxygenase-1 |
| NO.40 | PGR | P06401 | Progesterone receptor |
| NO.40 | ICMT | O60725 | Isoprenylcysteine carboxyl methyltransferase |
| NO.41 | CHRM1 | P11229 | Muscarinic acetylcholine receptor M1 |
| NO.41 | CHRM3 | P20309 | Muscarinic acetylcholine receptor M3 |
| NO.41 | CHRM2 | P08172 | Muscarinic acetylcholine receptor M2 |
| NO.41 | GABRA1 | [P14867](https://www.uniprot.org/uniprot/P14867) | Gamma-aminobutyric acid receptor subunit alpha-1 |
| NO.41 | UGT2B7 | P16662 | UDP-glucuronosyltransferase 2B7 |
| NO.41 | HSD11B1 | P28845 | 11-beta-hydroxysteroid dehydrogenase 1 |
| NO.41 | SHBG | P04278 | Testis-specific androgen-binding protein |
| NO.41 | AR | P10275 | Androgen Receptor |
| NO.41 | POLA1 | P09884 | DNA polymerase alpha subunit |
| NO.41 | NR1I3 | Q14994 | Nuclear receptor subfamily 1 group I member 3 |
| NO.41 | ESR1 | P03372 | Estrogen receptor alpha |
| NO.41 | CDC25A | P30304 | Dual specificity phosphatase Cdc25A |
| NO.41 | CDC25B | P30305 | Dual specificity phosphatase Cdc25B |
| NO.41 | SHH | Q15465 | Sonic hedgehog protein (by homology) |
| NO.41 | ESR2 | Q92731 | Estrogen receptor beta |
| NO.41 | TRPM8 | Q7Z2W7 | Transient receptor potential cation channel subfamily M member 8 |
| NO.41 | NR1H3 | Q13133 | LXR-alpha |
| NO.41 | CA2 | P00918 | Carbonic anhydrase II |
| NO.41 | CA1 | P00915 | Carbonic anhydrase I |
| NO.41 | CA4 | P22748 | Carbonic anhydrase IV |
| NO.41 | IDO1 | P14902 | Indoleamine 2,3-dioxygenase |
| NO.41 | NPC1L1 | Q9UHC9 | Niemann-Pick C1-like protein 1 |
| NO.41 | GPBAR1 | Q8TDU6 | G-protein coupled bile acid receptor 1 |
| NO.41 | CYP17A1 | P05093 | Cytochrome P450 17A1 |
| NO.41 | CYP19A1 | P11511 | Cytochrome P450 19A1 |
| NO.41 | NR1H4 | Q96RI1 | Bile acid receptor FXR |
| NO.42 | CHRM4 | P08173 | Muscarinic acetylcholine receptor M4 |
| NO.42 | CHRM5 | P08912 | Muscarinic acetylcholine receptor M5 |
| NO.42 | CHRM1 | P11229 | Muscarinic acetylcholine receptor M1 |
| NO.42 | CHRM3 | P20309 | Muscarinic acetylcholine receptor M3 |
| NO.42 | ACHE | P22303 | Acetylcholinesterase |
| NO.42 | CHRM2 | P08172 | Muscarinic acetylcholine receptor M2 |
| NO.42 | DRD1 | P21728 | Dopamine D1 receptor |
| NO.42 | HTR1D | P28221 | Serotonin 1d (5-HT1d) receptor |
| NO.42 | AURKB | Q96GD4 | Serine/threonine-protein kinase Aurora-B |
| NO.42 | RPS6KB1 | P23443 | Ribosomal protein S6 kinase 1 |
| NO.42 | AURKA | O14965 | Serine/threonine-protein kinase Aurora-A |
| NO.42 | PNP | P00491 | Purine nucleoside phosphorylase |
| NO.42 | ADRB2 | P07550 | Adrenergic receptor beta |
| NO.42 | ADRB1 | P08588 | Beta-1 adrenergic receptor |
| NO.42 | ADRB3 | P13945 | Beta-3 adrenergic receptor |
| NO.42 | DPP7 | Q9UHL4 | Dipeptidyl peptidase II |
| NO.42 | FAP | Q12884 | Fibroblast activation protein alpha |
| NO.42 | HRH4 | Q9H3N8 | Histamine H4 receptor |
| NO.42 | KCNJ1 | P48048 | ATP-sensitive inward rectifier potassium channel 1 |
| NO.42 | OPRL1 | P41146 | Nociceptin receptor |
| NO.42 | TRPV3 | Q8NET8 | Transient receptor potential cation channel subfamily V member 3 |
| NO.42 | CHRNA3 | P32297 | Neuronal acetylcholine receptor subunit alpha-3 |
| NO.42 | MTAP | Q13126 | S-methyl-5-thioadenosine phosphorylase |
| NO.42 | PDE9A | O76083 | Phosphodiesterase 9A |
| NO.42 | PDE1C | Q14123 | Phosphodiesterase 1C |
| NO.42 | HTR2C | P28335 | Serotonin 2c (5-HT2c) receptor |
| NO.42 | ROCK2 | O75116 | Rho-associated protein kinase 2 |
| NO.42 | PDE5A | O76074 | Phosphodiesterase 5A |
| NO.42 | MAN1B1 | Q9UKM7 | Endoplasmic reticulum mannosyl-oligosaccharide 1,2-alpha-mannosidase |
| NO.42 | PDE11A | Q9HCR9 | Phosphodiesterase 11A |
| NO.42 | MAN2A1 | Q16706 | Alpha-mannosidase 2A1 |
| NO.42 | PDE10A | Q9Y233 | Phosphodiesterase 10A |
| NO.42 | ADRA1D | P25100 | Alpha-1d adrenergic receptor |
| NO.42 | LRRK2 | Q5S007 | Leucine-rich repeat serine/threonine-protein kinase 2 |
| NO.42 | METAP2 | P50579 | Methionine aminopeptidase 2 |
| NO.42 | HTR1F | P30939 | Serotonin 1f (5-HT1f) receptor |
| NO.42 | HTR1B | P28222 | Serotonin 1b (5-HT1b) receptor |
| NO.42 | HTR1A | P08908 | Serotonin 1a (5-HT1a) receptor |
| NO.42 | ADORA1 | P30542 | Adenosine A1 receptor |
| NO.42 | HTR7 | P34969 | Serotonin 7 (5-HT7) receptor |
| NO.42 | EHMT1 | Q9H9B1 | Histone-lysine N-methyltransferase, H3 lysine-9 specific 5 |
| NO.42 | EHMT2 | Q96KQ7 | Histone-lysine N-methyltransferase, H3 lysine-9 specific 3 |
| NO.42 | PNMT | P11086 | Phenylethanolamine N-methyltransferase |
| NO.42 | PLG | P00747 | Plasminogen |
| NO.42 | TOP2A | P11388 | DNA topoisomerase II alpha |
| NO.42 | NOS2 | P35228 | Nitric oxide synthase, inducible |
| NO.42 | BACE1 | P56817 | Beta-secretase 1 |
| NO.42 | NMT1 | P30419 | Peptide N-myristoyltransferase 1 |
| NO.42 | SCN9A | Q15858 | Sodium channel protein type IX alpha subunit |
| NO.42 | PRMT6 | Q96LA8 | Protein arginine N-methyltransferase 6 |
| NO.42 | PRMT8 | Q9NR22 | Protein arginine N-methyltransferase 8 |
| NO.42 | PRMT1 | Q99873 | Protein-arginine N-methyltransferase 1 |
| NO.42 | EGFR | P00533 | Epidermal growth factor receptor erbB1 |
| NO.42 | MAPK14 | Q16539 | MAP kinase p38 alpha (by homology) |
| NO.42 | PARP1 | P09874 | Poly [ADP-ribose] polymerase-1 |
| NO.42 | PFKFB4 | Q16877 | Fructose-2,6-bisphosphatase 4 |
| NO.42 | PFKFB3 | Q16875 | Fructose-2,6-bisphosphatase 3 |
| NO.42 | MAPK11 | Q15759 | MAP kinase p38 beta |
| NO.42 | MAPK9 | P45984 | c-Jun N-terminal kinase 2 |
| NO.42 | FLT3 | P36888 | Tyrosine-protein kinase receptor FLT3 |
| NO.42 | HTR4 | Q13639 | Serotonin 4 (5-HT4) receptor |
| NO.42 | HTR1E | P28566 | Serotonin 1e (5-HT1e) receptor |
| NO.42 | ADRA1B | P35368 | Alpha-1b adrenergic receptor |
| NO.42 | P2RX7 | Q99572 | P2X purinoceptor 7 |
| NO.42 | MDM2 | Q00987 | p53-binding protein Mdm-2 |
| NO.42 | DRD4 | P21917 | Dopamine D4 receptor |
| NO.42 | F10 | P00742 | Thrombin and coagulation factor X |
| NO.42 | CTRB1 | P17538 | Beta-chymotrypsin |
| NO.42 | CHIA | Q9BZP6 | Acidic mammalian chitinase |
| NO.42 | SLC6A4 | P31645 | Serotonin transporter |
| NO.42 | GRIN1 | Q05586 | Glutamate receptor ionotropic, NMDA 1 |
| NO.42 | GRIN2B | Q13224 | Glutamate receptor ionotropic, NMDA 2B |
| NO.42 | MAPK8 | P45983 | c-Jun N-terminal kinase 1 |
| NO.42 | GSK3B | P49841 | Glycogen synthase kinase-3 beta |
| NO.42 | DUSP3 | P51452 | Dual specificity protein phosphatase 3 |
| NO.42 | GSK3A | P49840 | Glycogen synthase kinase-3 alpha |
| NO.42 | CA2 | P00918 | Carbonic anhydrase II |
| NO.42 | ERAP2 | Q6P179 | Endoplasmic reticulum aminopeptidase 2 |
| NO.42 | ERAP1 | Q9NZ08 | Endoplasmic reticulum aminopeptidase 1 |
| NO.42 | ROCK1 | Q13464 | Rho-associated protein kinase 1 |
| NO.42 | PABPC1 | P11940 | Polyadenylate-binding protein 1 |
| NO.42 | PRKCI | P41743 | Protein kinase C iota |
| NO.42 | ILK | Q13418 | Serine/threonine-protein kinase ILK-1 |
| NO.42 | BIRC2 | Q13490 | Baculoviral IAP repeat-containing protein 2 |
| NO.42 | MPI | P34949 | Mannose-6-phosphate isomerase |
| NO.42 | QRFPR | Q96P65 | Pyroglutamylated RFamide peptide receptor |
| NO.42 | FLT1 | P17948 | Vascular endothelial growth factor receptor 1 |
| NO.42 | KIT | P10721 | Stem cell growth factor receptor |
| NO.42 | KDR | P35968 | Vascular endothelial growth factor receptor 2 |
| NO.42 | MMP13 | P45452 | Matrix metalloproteinase 13 |
| NO.42 | MTOR | P42345 | Serine/threonine-protein kinase mTOR |
| NO.42 | PIK3CD | O00329 | PI3-kinase p110-delta subunit |
| NO.42 | MMP1 | P03956 | Matrix metalloproteinase 1 |
| NO.42 | PIK3CA | P42336 | PI3-kinase p110-alpha subunit |
| NO.42 | MMP7 | P09237 | Matrix metalloproteinase 7 |
| NO.42 | MMP12 | P39900 | Matrix metalloproteinase 12 |
| NO.42 | MMP8 | P22894 | Matrix metalloproteinase 8 |
| NO.42 | TKT | P29401 | Transketolase |
| NO.42 | CHEK2 | O96017 | Serine/threonine-protein kinase Chk2 |
| NO.42 | NUDT1 | P36639 | 7,8-dihydro-8-oxoguanine triphosphatase |
| NO.42 | CHEK1 | O14757 | Serine/threonine-protein kinase Chk1 |
| NO.42 | ACVRL1 | P37023 | Serine/threonine-protein kinase receptor R3 |
| NO.43 | CHRM3 | P20309 | Muscarinic acetylcholine receptor M3 |
| NO.43 | CHRM2 | P08172 | Muscarinic acetylcholine receptor M2 |
| NO.43 | UGT2B7 | P16662 | UDP-glucuronosyltransferase 2B7 |
| NO.43 | HSD11B1 | P28845 | 11-beta-hydroxysteroid dehydrogenase 1 |
| NO.43 | IDO1 | P14902 | Indoleamine 2,3-dioxygenase |
| NO.43 | PTGS1 | P23219 | Cyclooxygenase-1 |
| NO.43 | PGR | P06401 | Progesterone receptor |
| NO.43 | ICMT | O60725 | Isoprenylcysteine carboxyl methyltransferase |
| NO.44 | CHRM1 | P11229 | Muscarinic acetylcholine receptor M1 |
| NO.44 | CHRM3 | P20309 | Muscarinic acetylcholine receptor M3 |
| NO.44 | CHRM2 | P08172 | Muscarinic acetylcholine receptor M2 |
| NO.44 | UGT2B7 | P16662 | UDP-glucuronosyltransferase 2B7 |
| NO.44 | HSD11B1 | P28845 | 11-beta-hydroxysteroid dehydrogenase 1 |
| NO.44 | IDO1 | P14902 | Indoleamine 2,3-dioxygenase |
| NO.44 | PTGS1 | P23219 | Cyclooxygenase-1 |
| NO.44 | PGR | P06401 | Progesterone receptor |
| NO.44 | ICMT | O60725 | Isoprenylcysteine carboxyl methyltransferase |
| NO.45 | CHRM3 | [P20309](https://www.uniprot.org/uniprot/P20309) | Muscarinic acetylcholine receptor M3 |
| NO.45 | CHRM1 | P11229 | Muscarinic acetylcholine receptor M1 |
| NO.45 | GABRA2 | P47869 | Gamma-aminobutyric acid receptor subunit alpha-2 |
| NO.45 | GABRA1 | [P14867](https://www.uniprot.org/uniprot/P14867) | Gamma-aminobutyric acid receptor subunit alpha-1 |
| NO.45 | GABRA6 | Q16445 | Gamma-aminobutyric-acid receptor subunit alpha-6 |
| NO.45 | ADRA1B | P35368 | Alpha-1B adrenergic receptor |
| NO.45 | CHRNA7 | P36544 | Neuronal acetylcholine receptor protein, alpha-7 chain |
| NO.45 | CYP19A1 | P11511 | Cytoc+A2:A44hrome P450 19A1 |
| NO.45 | PTPN1 | P18031 | Protein-tyrosine phosphatase 1B |
| NO.45 | NR1H3 | Q13133 | LXR-alpha |
| NO.45 | CYP17A1 | P05093 | Cytochrome P450 17A1 |
| NO.45 | AR | P10275 | Androgen Receptor (by homology) |
| NO.45 | ESR1 | P03372 | Estrogen receptor alpha |
| NO.45 | CHRM2 | P08172 | Muscarinic acetylcholine receptor M2 |
| NO.45 | ACHE | P22303 | Acetylcholinesterase |
| NO.45 | SLC6A2 | P23975 | Norepinephrine transporter |
| NO.45 | SLC6A4 | P31645 | Serotonin transporter |
| NO.45 | CYP2C19 | P33261 | Cytochrome P450 2C19 |
| NO.45 | RORC | P51449 | Nuclear receptor ROR-gamma |
| NO.45 | SREBF2 | Q12772 | Sterol regulatory element-binding protein 2 |
| NO.45 | NPC1L1 | Q9UHC9 | Niemann-Pick C1-like protein 1 |
| NO.45 | CYP51A1 | Q16850 | Cytochrome P450 51 (by homology) |
| NO.45 | HMGCR | P04035 | HMG-CoA reductase |
| NO.45 | NR1I3 | Q14994 | Nuclear receptor subfamily 1 group I member 3 |
| NO.45 | BCHE | P06276 | Butyrylcholinesterase |
| NO.45 | HSD11B1 | P28845 | 11-beta-hydroxysteroid dehydrogenase 1 |
| NO.45 | ESR2 | Q92731 | Estrogen receptor beta |
| NO.45 | SQLE | Q14534 | Squalene monooxygenase |
| NO.45 | SHBG | P04278 | Testis-specific androgen-binding protein |
| NO.45 | POLB | P06746 | DNA polymerase beta |
| NO.45 | PDE4D | Q08499 | Phosphodiesterase 4D |
| NO.45 | CES2 | O00748 | Carboxylesterase 2 |
| NO.45 | PTPRF | P10586 | Receptor-type tyrosine-protein phosphatase F (LAR) |
| NO.45 | PTPN2 | P17706 | T-cell protein-tyrosine phosphatase |
| NO.45 | PLA2G1B | P04054 | Phospholipase A2 group 1B |
| NO.45 | ACP1 | P24666 | Low molecular weight phosphotyrosine protein phosphatase |
| NO.45 | AKR1B10 | O60218 | Aldo-keto reductase family 1 member B10 |
| NO.45 | UGT2B7 | P16662 | UDP-glucuronosyltransferase 2B7 |
| NO.45 | CDC25B | P30305 | Dual specificity phosphatase Cdc25B |
| NO.45 | ATP12A | P54707 | Potassium-transporting ATPase alpha chain 2 |
| NO.45 | PTPN6 | P29350 | Protein-tyrosine phosphatase 1C |
| NO.45 | RORA | P35398 | Nuclear receptor ROR-alpha |
| NO.45 | CD81 | P60033 | CD81 antigen |
| NO.45 | G6PD | P11413 | Glucose-6-phosphate 1-dehydrogenase |
| NO.45 | PREP | P48147 | Prolyl endopeptidase |
| NO.45 | FABP4 | P15090 | Fatty acid binding protein adipocyte |
| NO.45 | PPARG | P37231 | Peroxisome proliferator-activated receptor gamma |
| NO.45 | PPARA | Q07869 | Peroxisome proliferator-activated receptor alpha |
| NO.45 | TERT | O14746 | Telomerase reverse transcriptase |
| NO.45 | FABP3 | P05413 | Fatty acid binding protein muscle |
| NO.45 | FABP5 | Q01469 | Fatty acid binding protein epidermal |
| NO.45 | HSD11B2 | P80365 | 11-beta-hydroxysteroid dehydrogenase 2 |
| NO.45 | PPARD | Q03181 | Peroxisome proliferator-activated receptor delta |
| NO.45 | FABP1 | P07148 | Fatty acid-binding protein, liver |
| NO.45 | FNTA | P49354 | Protein farnesyltransferase/geranylgeranyltransferase type-1 subunit alpha |
| NO.45 | FNTB | P49356 | Protein farnesyltransferase subunit beta |
| NO.45 | PTPN11 | Q06124 | Protein-tyrosine phosphatase 2C |
| NO.46 | CHRM3 | [P20309](https://www.uniprot.org/uniprot/P20309) | Muscarinic acetylcholine receptor M3 |
| NO.46 | CHRM1 | P11229 | Muscarinic acetylcholine receptor M1 |
| NO.46 | SLC6A2 | P23975 | Sodium-dependent noradrenaline transporter |
| NO.46 | GABRA2 | P47869 | Gamma-aminobutyric acid receptor subunit alpha-2 |
| NO.46 | GABRA1 | [P14867](https://www.uniprot.org/uniprot/P14867) | Gamma-aminobutyric acid receptor subunit alpha-1 |
| NO.46 | GABRA6 | Q16445 | Gamma-aminobutyric-acid receptor subunit alpha-6 |
| NO.46 | ADRA1B | P35368 | Alpha-1B adrenergic receptor |
| NO.46 | CHRNA7 | P36544 | Neuronal acetylcholine receptor protein, alpha-7 chain |
| NO.46 | CYP19A1 | P11511 | Cytoc+A2:A44hrome P450 19A1 |
| NO.46 | PTPN1 | P18031 | Protein-tyrosine phosphatase 1B |
| NO.46 | NR1H3 | Q13133 | LXR-alpha |
| NO.46 | CYP17A1 | P05093 | Cytochrome P450 17A1 |
| NO.46 | AR | P10275 | Androgen Receptor (by homology) |
| NO.46 | ESR1 | P03372 | Estrogen receptor alpha |
| NO.46 | CHRM2 | P08172 | Muscarinic acetylcholine receptor M2 |
| NO.46 | ACHE | P22303 | Acetylcholinesterase |
| NO.46 | SLC6A4 | P31645 | Serotonin transporter |
| NO.46 | CYP2C19 | P33261 | Cytochrome P450 2C19 |
| NO.46 | RORC | P51449 | Nuclear receptor ROR-gamma |
| NO.46 | SREBF2 | Q12772 | Sterol regulatory element-binding protein 2 |
| NO.46 | NPC1L1 | Q9UHC9 | Niemann-Pick C1-like protein 1 |
| NO.46 | CYP51A1 | Q16850 | Cytochrome P450 51 (by homology) |
| NO.46 | HMGCR | P04035 | HMG-CoA reductase |
| NO.46 | NR1I3 | Q14994 | Nuclear receptor subfamily 1 group I member 3 |
| NO.46 | BCHE | P06276 | Butyrylcholinesterase |
| NO.46 | HSD11B1 | P28845 | 11-beta-hydroxysteroid dehydrogenase 1 |
| NO.46 | ESR2 | Q92731 | Estrogen receptor beta |
| NO.46 | SQLE | Q14534 | Squalene monooxygenase |
| NO.46 | SHBG | P04278 | Testis-specific androgen-binding protein |
| NO.46 | POLB | P06746 | DNA polymerase beta |
| NO.46 | PDE4D | Q08499 | Phosphodiesterase 4D |
| NO.46 | CES2 | O00748 | Carboxylesterase 2 |
| NO.46 | PTPRF | P10586 | Receptor-type tyrosine-protein phosphatase F (LAR) |
| NO.46 | PTPN2 | P17706 | T-cell protein-tyrosine phosphatase |
| NO.46 | PLA2G1B | P04054 | Phospholipase A2 group 1B |
| NO.46 | ACP1 | P24666 | Low molecular weight phosphotyrosine protein phosphatase |
| NO.46 | AKR1B10 | O60218 | Aldo-keto reductase family 1 member B10 |
| NO.46 | UGT2B7 | P16662 | UDP-glucuronosyltransferase 2B7 |
| NO.46 | CDC25B | P30305 | Dual specificity phosphatase Cdc25B |
| NO.46 | ATP12A | P54707 | Potassium-transporting ATPase alpha chain 2 |
| NO.46 | PTPN6 | P29350 | Protein-tyrosine phosphatase 1C |
| NO.46 | RORA | P35398 | Nuclear receptor ROR-alpha |
| NO.46 | CD81 | P60033 | CD81 antigen |
| NO.46 | G6PD | P11413 | Glucose-6-phosphate 1-dehydrogenase |
| NO.46 | PREP | P48147 | Prolyl endopeptidase |
| NO.46 | FABP4 | P15090 | Fatty acid binding protein adipocyte |
| NO.46 | PPARG | P37231 | Peroxisome proliferator-activated receptor gamma |
| NO.46 | PPARA | Q07869 | Peroxisome proliferator-activated receptor alpha |
| NO.46 | TERT | O14746 | Telomerase reverse transcriptase |
| NO.46 | FABP3 | P05413 | Fatty acid binding protein muscle |
| NO.46 | FABP5 | Q01469 | Fatty acid binding protein epidermal |
| NO.46 | HSD11B2 | P80365 | 11-beta-hydroxysteroid dehydrogenase 2 |
| NO.46 | PPARD | Q03181 | Peroxisome proliferator-activated receptor delta |
| NO.46 | FABP1 | P07148 | Fatty acid-binding protein, liver |
| NO.46 | FNTA | P49354 | Protein farnesyltransferase/geranylgeranyltransferase type-1 subunit alpha |
| NO.46 | FNTB | P49356 | Protein farnesyltransferase subunit beta |
| NO.46 | PTPN11 | Q06124 | Protein-tyrosine phosphatase 2C |
| NO.47 | CHRM1 | P11229 | Muscarinic acetylcholine receptor M1 |
| NO.47 | GABRA2 | P47869 | Gamma-aminobutyric acid receptor subunit alpha-2 |
| NO.47 | SLC6A2 | P23975 | Sodium-dependent noradrenaline transporter |
| NO.47 | GABRA1 | [P14867](https://www.uniprot.org/uniprot/P14867) | Gamma-aminobutyric acid receptor subunit alpha-1 |
| NO.47 | GABRA6 | Q16445 | Gamma-aminobutyric-acid receptor subunit alpha-6 |
| NO.47 | CHRNA7 | P36544 | Neuronal acetylcholine receptor protein, alpha-7 chain |
| NO.47 | CYP19A1 | P11511 | Cytoc+A2:A44hrome P450 19A1 |
| NO.47 | PTPN1 | P18031 | Protein-tyrosine phosphatase 1B |
| NO.47 | NR1H3 | Q13133 | LXR-alpha |
| NO.47 | CYP17A1 | P05093 | Cytochrome P450 17A1 |
| NO.47 | AR | P10275 | Androgen Receptor (by homology) |
| NO.47 | ESR1 | P03372 | Estrogen receptor alpha |
| NO.47 | CHRM2 | P08172 | Muscarinic acetylcholine receptor M2 |
| NO.47 | ACHE | P22303 | Acetylcholinesterase |
| NO.47 | SLC6A4 | P31645 | Serotonin transporter |
| NO.47 | CYP2C19 | P33261 | Cytochrome P450 2C19 |
| NO.47 | RORC | P51449 | Nuclear receptor ROR-gamma |
| NO.47 | SREBF2 | Q12772 | Sterol regulatory element-binding protein 2 |
| NO.47 | NPC1L1 | Q9UHC9 | Niemann-Pick C1-like protein 1 |
| NO.47 | CYP51A1 | Q16850 | Cytochrome P450 51 (by homology) |
| NO.47 | HMGCR | P04035 | HMG-CoA reductase |
| NO.47 | NR1I3 | Q14994 | Nuclear receptor subfamily 1 group I member 3 |
| NO.47 | BCHE | P06276 | Butyrylcholinesterase |
| NO.47 | HSD11B1 | P28845 | 11-beta-hydroxysteroid dehydrogenase 1 |
| NO.47 | ESR2 | Q92731 | Estrogen receptor beta |
| NO.47 | SQLE | Q14534 | Squalene monooxygenase |
| NO.47 | SHBG | P04278 | Testis-specific androgen-binding protein |
| NO.47 | POLB | P06746 | DNA polymerase beta |
| NO.47 | PDE4D | Q08499 | Phosphodiesterase 4D |
| NO.47 | CES2 | O00748 | Carboxylesterase 2 |
| NO.47 | PTPRF | P10586 | Receptor-type tyrosine-protein phosphatase F (LAR) |
| NO.47 | PTPN2 | P17706 | T-cell protein-tyrosine phosphatase |
| NO.47 | PLA2G1B | P04054 | Phospholipase A2 group 1B |
| NO.47 | ACP1 | P24666 | Low molecular weight phosphotyrosine protein phosphatase |
| NO.47 | AKR1B10 | O60218 | Aldo-keto reductase family 1 member B10 |
| NO.47 | UGT2B7 | P16662 | UDP-glucuronosyltransferase 2B7 |
| NO.47 | CDC25B | P30305 | Dual specificity phosphatase Cdc25B |
| NO.47 | ATP12A | P54707 | Potassium-transporting ATPase alpha chain 2 |
| NO.47 | PTPN6 | P29350 | Protein-tyrosine phosphatase 1C |
| NO.47 | RORA | P35398 | Nuclear receptor ROR-alpha |
| NO.47 | CD81 | P60033 | CD81 antigen |
| NO.47 | G6PD | P11413 | Glucose-6-phosphate 1-dehydrogenase |
| NO.47 | PREP | P48147 | Prolyl endopeptidase |
| NO.47 | FABP4 | P15090 | Fatty acid binding protein adipocyte |
| NO.47 | PPARG | P37231 | Peroxisome proliferator-activated receptor gamma |
| NO.47 | PPARA | Q07869 | Peroxisome proliferator-activated receptor alpha |
| NO.47 | TERT | O14746 | Telomerase reverse transcriptase |
| NO.47 | FABP3 | P05413 | Fatty acid binding protein muscle |
| NO.47 | FABP5 | Q01469 | Fatty acid binding protein epidermal |
| NO.47 | HSD11B2 | P80365 | 11-beta-hydroxysteroid dehydrogenase 2 |
| NO.47 | PPARD | Q03181 | Peroxisome proliferator-activated receptor delta |
| NO.47 | FABP1 | P07148 | Fatty acid-binding protein, liver |
| NO.47 | FNTA | P49354 | Protein farnesyltransferase/geranylgeranyltransferase type-1 subunit alpha |
| NO.47 | FNTB | P49356 | Protein farnesyltransferase subunit beta |
| NO.47 | PTPN11 | Q06124 | Protein-tyrosine phosphatase 2C |
| NO.48 | PTGS1 | P23219 | Prostaglandin G/H synthase 1 |
| NO.48 | PTGS2 | P35354 | Prostaglandin G/H synthase 2 |
| NO.48 | SLC6A2 | P23975 | Sodium-dependent noradrenaline transporter |
| NO.48 | SLC6A3 | Q01959 | Sodium-dependent dopamine transporter |
| NO.48 | CHRM1 | [P11229](https://www.uniprot.org/uniprot/P11229) | Muscarinic acetylcholine receptor M1 |
| NO.48 | PDE3A | Q14432 | CGMP-inhibited 3',5'-cyclic phosphodiesterase A |
| NO.48 | ADRA1A | P35348 | Alpha-1A adrenergic receptor |
| NO.48 | CYP2A6 | P11509 | Cytochrome P450 2A6 |
| NO.48 | ALB | P02768 | Serum albumin |
| NO.48 | GABRB1 | P18505 | Gamma-aminobutyric acid receptor subunit beta-1 |
| NO.48 | PMM2 | O15305 | Phosphomannomutase 2 |
| NO.48 | SLC35E2B | P0CK96 | Solute carrier family 35 member E2B |
| NO.48 | SLC35E2A | P0CK97 | Solute carrier family 35 member E2A |
| NO.48 | SLC35E1 | Q96K37 | Solute carrier family 35 member E1 |
| NO.48 | SLC35E4 | Q6ICL7 | Solute carrier family 35 member E4 |
| NO.48 | SLC35E3 | Q7Z769 | Solute carrier family 35 member E3 |
| NO.49 | CA4 | P22748 | Carbonic anhydrase IV |
| NO.49 | CYP19A1 | P11511 | Cytochrome P450 19A1 |
| NO.49 | CES1 | P23141 | Acyl coenzyme A:cholesterol acyltransferase |
| NO.49 | CES2 | O00748 | Carboxylesterase 2 |
| NO.49 | CA2 | P00918 | Carbonic anhydrase II |
| NO.49 | NR1I3 | Q14994 | Nuclear receptor subfamily 1 group I member 3 (by homology) |
| NO.49 | CA1 | P00915 | Carbonic anhydrase I |
| NO.49 | AR | P10275 | Androgen Receptor |
| NO.49 | EPHX1 | P07099 | Epoxide hydrolase 1 |
| NO.49 | PGR | P06401 | Progesterone receptor |
| NO.49 | SRD5A2 | P31213 | Steroid 5-alpha-reductase 2 |
| NO.49 | SRD5A1 | P18405 | Steroid 5-alpha-reductase 1 |
| NO.49 | PAOX | Q6QHF9 | Polyamine oxidase |
| NO.49 | ICMT | O60725 | Isoprenylcysteine carboxyl methyltransferase |
| NO.49 | TBXAS1 | P24557 | Thromboxane-A synthase |
| NO.49 | ALDH2 | P05091 | Aldehyde dehydrogenase |
| NO.49 | ACACB | O00763 | Acetyl-CoA carboxylase 2 |
| NO.49 | RORC | P51449 | Nuclear receptor ROR-gamma |
| NO.49 | SCN9A | Q15858 | Sodium channel protein type IX alpha subunit |
| NO.49 | MTNR1A | P48039 | Melatonin receptor 1A |
| NO.49 | MTNR1B | P49286 | Melatonin receptor 1B |
| NO.49 | KDR | P35968 | Vascular endothelial growth factor receptor 2 |
| NO.49 | MCHR1 | Q99705 | Melanin-concentrating hormone receptor 1 |
| NO.49 | HSD3B1 | P14060 | 3-beta-hydroxysteroid dehydrogenase/delta 5-->4-isomerase type I |
| NO.49 | ADH1B | P00325 | Alcohol dehydrogenase 1B |
| NO.49 | ADH1A | P07327 | Alcohol dehydrogenase 1A |
| NO.49 | ADH1C | P00326 | Alcohol dehydrogenase 1C |
| NO.49 | ADH7 | P40394 | Alcohol dehydrogenase class 4 mu/sigma chain |
| NO.49 | PAM | P19021 | Peptidyl-glycine alpha-amidating monooxygenase |
| NO.49 | CA3 | P07451 | Carbonic anhydrase 3 |
| NO.49 | DNM1 | Q05193 | Dynamin-1 |
| NO.49 | FAAH | O00519 | Fatty-acid amide hydrolase 1 |
| NO.49 | FABP3 | P05413 | Fatty acid-binding protein, heart |
| NO.49 | FDPS | P14324 | Farnesyl pyrophosphate synthase |
| NO.49 | GGPS1 | O95749 | Geranylgeranyl pyrophosphate synthase |
| NO.49 | GNAI1 | P63096 | Guanine nucleotide-binding protein G(i) subunit alpha-1 |
| NO.49 | GNAO1 | P09471 | Guanine nucleotide-binding protein G(o) subunit alpha |
| NO.49 | GNAI3 | P08754 | Guanine nucleotide-binding protein G(k) subunit alpha |
| NO.49 | HAO1 | Q9UJM8 | Hydroxyacid oxidase 1 |
| NO.49 | EPHX2 | P34913 | Bifunctional epoxide hydrolase 2 |
| NO.49 | LPAR1 | Q92633 | Lysophosphatidic acid receptor 1 |
| NO.49 | LPAR2 | Q9HBW0 | Lysophosphatidic acid receptor 2 |
| NO.49 | LPAR3 | Q9UBY5 | Lysophosphatidic acid receptor 3 |
| NO.49 | LPAR4 | Q99677 | Lysophosphatidic acid receptor 4 |
| NO.49 | LPAR6 | P43657 | Lysophosphatidic acid receptor 6 |
| NO.49 | MGLL | Q99685 | Monoglyceride lipase |
| NO.49 | MPEG1 | Q2M385 | Macrophage-expressed gene 1 protein |
| NO.49 | OXER1 | Q8TDS5 | Oxoeicosanoid receptor 1 |
| NO.49 | PAFAH1B2 | P68402 | Platelet-activating factor acetylhydrolase IB subunit beta |
| NO.49 | PLA2G4C | Q9UP65 | Cytosolic phospholipase A2 gamma |
| NO.49 | PLA2G2C | Q5R387 | Putative inactive group IIC secretory phospholipase A2 |
| NO.49 | S1PR2 | O95136 | Sphingosine 1-phosphate receptor 2 |
| NO.49 | S1PR4 | O95977 | Sphingosine 1-phosphate receptor 4 |
| NO.49 | S1PR3 | Q99500 | Sphingosine 1-phosphate receptor 3 |
| NO.49 | SLC22A1 | O15245 | Solute carrier family 22 member 1 |
| NO.49 | SLC22A8 | Q8TCC7 | Solute carrier family 22 member 8 |
| NO.49 | TLR2 | O60603 | Toll-like receptor 2 |
| NO.50 | SLC6A2 | P23975 | Sodium-dependent noradrenaline transporter |
| NO.50 | MAOB | [P27338](https://www.uniprot.org/uniprot/P27338) | Amine oxidase [flavin-containing] B |
| NO.50 | NCOA2 | Q15596 | Nuclear receptor coactivator 2 |
| NO.50 | RXRA | P19793 | Retinoic acid receptor RXR-alpha |
| NO.50 | PTGS1 | [P23219](https://www.uniprot.org/uniprot/P23219) | Prostaglandin G/H synthase 1 |
| NO.50 | IL6 | P05231 | Interleukin-6 |
| NO.50 | CASP3 | P42574 | Caspase-3 |
| NO.50 | IVL | P07476 | Involucrin |
| NO.50 | RASGRF2 | O14827 | Ras-specific guanine nucleotide-releasing factor 2 |
| NO.50 | BAK1 | Q16611 | Bcl-2 homologous antagonist/killer |
| NO.50 | TLR4 | O00206 | Toll-like receptor 4 |
| NO.50 | LPL | P06858 | Lipoprotein lipase |
| NO.50 | HMGCR | P04035 | 3-hydroxy-3-methylglutaryl-coenzyme A reductase |
| NO.50 | PPARA | Q07869 | Peroxisome proliferator-activated receptor alpha |
| NO.50 | TLR2 | O60603 | Toll-like receptor 2 |
| NO.50 | DEFB4A | O15263 | Beta-defensin 2 |
| NO.50 | HIRA | P54198 | Protein HIRA |
| NO.50 | SERTAD3 | Q9UJW9 | SERTA domain-containing protein 3 |
| NO.50 | NR1H4 | Q96RI1 | Bile acid receptor |
| NO.50 | GLS2 | Q9UI32 | Glutaminase liver isoform, mitochondrial |
| NO.50 | IGHD | P01880 | Ig delta chain C region |
| NO.50 | GGPS1 | O95749 | Geranylgeranyl pyrophosphate synthase |
| NO.50 | LSS | P48449 | Lanosterol synthase |
| NO.50 | FNTB | P49356 | Protein farnesyltransferase subunit beta |
| NO.50 | FNTA | P49354 | Protein farnesyltransferase/geranylgeranyltransferase type-1 subunit alpha |
| NO.50 | HSD11B1 | P28845 | 11-beta-hydroxysteroid dehydrogenase 1 |
| NO.50 | HSD11B2 | P80365 | 11-beta-hydroxysteroid dehydrogenase 2 |
| NO.50 | AKR1C3 | P42330 | Aldo-keto-reductase family 1 member C3 |
| NO.50 | AR | P10275 | Androgen Receptor |
| NO.50 | BACE1 | P56817 | Beta-secretase 1 |
| NO.50 | BRS3 | P32247 | Bombesin receptor subtype-3 |
| NO.50 | C5AR1 | P21730 | C5a anaphylatoxin chemotactic receptor |
| NO.50 | CNR1 | P21554 | Cannabinoid receptor 1 (by homology) |
| NO.50 | CNR2 | P34972 | Cannabinoid receptor 2 |
| NO.50 | PTGS2 | P35354 | Cyclooxygenase-2 |
| NO.50 | CYP11B1 | P15538 | Cytochrome P450 11B1 |
| NO.50 | CYP11B2 | P19099 | Cytochrome P450 11B2 |
| NO.50 | EPHX2 | P34913 | Epoxide hydratase |
| NO.50 | GABBR1 | Q9UBS5 | GABA-B receptor (by homology) |
| NO.50 | PSEN2 | P49810 | Presenilin-2 |
| NO.50 | PSENEN | Q9NZ42 | Gamma-secretase subunit PEN-2 |
| NO.50 | NCSTN | Q92542 | Nicastrin |
| NO.50 | APH1A | Q96BI3 | Gamma-secretase subunit APH-1A |
| NO.50 | PSEN1 | P49768 | Presenilin-1 |
| NO.50 | APH1B | Q8WW43 | Gamma-secretase |
| NO.50 | GCGR | P47871 | Glucagon receptor |
| NO.50 | G6PD | P11413 | Glucose-6-phosphate 1-dehydrogenase |
| NO.50 | KCNH2 | Q12809 | HERG |
| NO.50 | LIPE | Q05469 | Hormone sensitive lipase |
| NO.50 | PLA2G7 | Q13093 | LDL-associated phospholipase A2 |
| NO.50 | PYGL | P06737 | Liver glycogen phosphorylase |
| NO.50 | NR1H2 | P55055 | LXR-beta |
| NO.50 | MGLL | Q99685 | Monoglyceride lipase |
| NO.50 | MDM2 | Q00987 | p53-binding protein Mdm-2 |
| NO.50 | PER2 | O15055 | Period circadian protein homolog 2 |
| NO.50 | GPR88 | Q9GZN0 | Probable G-protein coupled receptor 88 |
| NO.50 | PGR | P06401 | Progesterone receptor |
| NO.50 | PRKCA | P17252 | Protein kinase C alpha |
| NO.50 | PRKCB | P05771 | Protein kinase C beta |
| NO.50 | PRKCD | Q05655 | Protein kinase C delta |
| NO.50 | PRKCE | Q02156 | Protein kinase C epsilon |
| NO.50 | PRKCH | P24723 | Protein kinase C eta |
| NO.50 | PRKCG | P05129 | Protein kinase C gamma |
| NO.50 | PRKCQ | Q04759 | Protein kinase C theta |
| NO.50 | F2R | P25116 | Proteinase-activated receptor 1 |
| NO.50 | RASGRP1 | O95267 | RAS guanyl-releasing protein 1 (by homology) |
| NO.50 | SIGMAR1 | Q99720 | Sigma opioid receptor |
| NO.50 | SQLE | Q14534 | Squalene monooxygenase |
| NO.50 | SHBG | P04278 | Testis-specific androgen-binding protein |
| NO.50 | TBXAS1 | P24557 | Thromboxane-A synthase |
| NO.50 | TRPA1 | O75762 | Transient receptor potential cation channel subfamily A member 1 |
| NO.50 | JAK2 | O60674 | Tyrosine-protein kinase JAK2 |
| NO.50 | JAK3 | P52333 | Tyrosine-protein kinase JAK3 |
| NO.50 | KDR | P35968 | Vascular endothelial growth factor receptor 2 |
| NO.50 | KCNA3 | P22001 | Voltage-gated potassium channel subunit Kv1.3 |
| NO.52 | ADH1A | P07327 | Alcohol dehydrogenase alpha chain |
| NO.52 | ADH1C | P00326 | Alcohol dehydrogenase gamma chain |
| NO.52 | ADORA2A | P29274 | Adenosine A2a receptor |
| NO.52 | AKR1C3 | P42330 | Aldo-keto-reductase family 1 member C3 |
| NO.52 | ALOX15 | P16050 | Arachidonate 15-lipoxygenase |
| NO.52 | ALOX5 | P09917 | Arachidonate 5-lipoxygenase |
| NO.52 | AVPR1A | P37288 | Vasopressin V1a receptor |
| NO.52 | AVPR2 | P30518 | Vasopressin V2 receptor |
| NO.52 | BRD2 | P25440 | Bromodomain-containing protein 2 |
| NO.52 | BRD3 | Q15059 | Bromodomain-containing protein 3 |
| NO.52 | BRD4 | O60885 | Bromodomain-containing protein 4 |
| NO.52 | CA1 | P00915 | Carbonic anhydrase I |
| NO.52 | CA12 | O43570 | Carbonic anhydrase XII |
| NO.52 | CA7 | P43166 | Carbonic anhydrase VII |
| NO.52 | CA9 | Q16790 | Carbonic anhydrase IX |
| NO.52 | CACNA1B | Q00975 | Voltage-gated N-type calcium channel alpha-1B subunit |
| NO.52 | CACNA1I | Q9P0X4 | Voltage-gated T-type calcium channel alpha-1I subunit |
| NO.52 | CACNA2D1 | P54289 | Voltage-gated calcium channel alpha2/delta subunit 1 |
| NO.52 | CCNC | P24863 | Cyclin C |
| NO.52 | CCR2 | P41597 | C-C chemokine receptor type 2 |
| NO.52 | CDK8 | P49336 | Cell division protein kinase 8 |
| NO.52 | CHRM1 | P11229 | Muscarinic acetylcholine receptor M1 (by homology) |
| NO.52 | CHRM2 | P08172 | Muscarinic acetylcholine receptor M2 (by homology) |
| NO.52 | CHRNA3 | P32297 | Neuronal acetylcholine receptor subunit alpha-3 |
| NO.52 | CHRNB4 | P30926 | Neuronal acetylcholine receptor subunit beta-4 |
| NO.52 | CHRNA4 | P43681 | Neuronal acetylcholine receptor subunit alpha-4 |
| NO.52 | CHRNB2 | P17787 | Neuronal acetylcholine receptor subunit beta-2 |
| NO.52 | CHRNA7 | P36544 | Neuronal acetylcholine receptor protein alpha-7 subunit |
| NO.52 | CNR2 | P34972 | Cannabinoid receptor 2 |
| NO.52 | CREBBP | Q92793 | CREB-binding protein/p53 |
| NO.52 | CRHR1 | P34998 | Corticotropin releasing factor receptor 1 (by homology) |
| NO.52 | CTSB | P07858 | Cathepsin (B and K) |
| NO.52 | CTSK | P43235 | Cathepsin K |
| NO.52 | CTSL | P07711 | Cathepsin L |
| NO.52 | CTSV | O60911 | Cathepsin (V and K) |
| NO.52 | CYP11B1 | P15538 | Cytochrome P450 11B1 |
| NO.52 | CYP11B2 | P19099 | Cytochrome P450 11B2 |
| NO.52 | CYP17A1 | P05093 | Cytochrome P450 17A1 |
| NO.52 | CYP19A1 | P11511 | Cytochrome P450 19A1 |
| NO.52 | CYP24A1 | Q07973 | Cytochrome P450 24A1 |
| NO.52 | CYP27A1 | Q02318 | Sterol 26-hydroxylase, mitochondrial |
| NO.52 | CYP2C19 | P33261 | Cytochrome P450 2C19 |
| NO.52 | DRD1 | P21728 | Dopamine D1 receptor |
| NO.52 | DRD2 | P14416 | Dopamine D2 receptor |
| NO.52 | DUSP3 | P51452 | Dual specificity protein phosphatase 3 |
| NO.52 | EGFR | P00533 | Epidermal growth factor receptor erbB1 |
| NO.52 | EPHX1 | P07099 | Epoxide hydrolase 1 |
| NO.52 | EPHX2 | P34913 | Epoxide hydratase |
| NO.52 | FKBP1A | P62942 | FK506-binding protein 1A |
| NO.52 | FLT4 | P35916 | Vascular endothelial growth factor receptor 3 |
| NO.52 | GLI1 | P08151 | Zinc finger protein GLI1 |
| NO.52 | GLI2 | P10070 | Zinc finger protein GLI2 |
| NO.52 | GRM1 | Q13255 | Metabotropic glutamate receptor 1 |
| NO.52 | GRM2 | Q14416 | Metabotropic glutamate receptor 2 |
| NO.52 | GRM5 | P41594 | Metabotropic glutamate receptor 5 (by homology) |
| NO.52 | GSK3B | P49841 | Glycogen synthase kinase-3 beta |
| NO.52 | HCRTR1 | O43613 | Orexin receptor 1 |
| NO.52 | HCRTR2 | O43614 | Orexin receptor 2 |
| NO.52 | HRH1 | P35367 | Histamine H1 receptor |
| NO.52 | HSD17B1 | P14061 | Estradiol 17-beta-dehydrogenase 1 |
| NO.52 | HSD17B2 | P37059 | Estradiol 17-beta-dehydrogenase 2 |
| NO.52 | HTR6 | P50406 | Serotonin 6 (5-HT6) receptor |
| NO.52 | IGF1R | P08069 | Insulin-like growth factor I receptor |
| NO.52 | IKBKB | O14920 | Inhibitor of nuclear factor kappa B kinase beta subunit |
| NO.52 | KCNA5 | P22460 | Voltage-gated potassium channel subunit Kv1.5 |
| NO.52 | KCNH2 | Q12809 | HERG |
| NO.52 | KDR | P35968 | Vascular endothelial growth factor receptor 2 |
| NO.52 | MAOA | P21397 | Monoamine oxidase A |
| NO.52 | MAOB | P27338 | Monoamine oxidase B |
| NO.52 | MAPK14 | Q16539 | MAP kinase p38 alpha |
| NO.52 | MGLL | Q99685 | Monoglyceride lipase |
| NO.52 | MMP1 | P03956 | Matrix metalloproteinase 1 |
| NO.52 | MMP13 | P45452 | Matrix metalloproteinase 13 |
| NO.52 | MMP9 | P14780 | Matrix metalloproteinase 9 |
| NO.52 | MTNR1A | P48039 | Melatonin receptor 1A |
| NO.52 | MTNR1B | P49286 | Melatonin receptor 1B |
| NO.52 | NMT1 | P30419 | Peptide N-myristoyltransferase 1 |
| NO.52 | NQO2 | P16083 | Quinone reductase 2 |
| NO.52 | NR3C1 | P04150 | Glucocorticoid receptor |
| NO.52 | P2RX7 | Q99572 | P2X purinoceptor 7 |
| NO.52 | PARP1 | P09874 | Poly [ADP-ribose] polymerase-1 |
| NO.52 | PDE4A | P27815 | Phosphodiesterase 4A |
| NO.52 | PDE4B | Q07343 | Phosphodiesterase 4B |
| NO.52 | PIM1 | P11309 | Serine/threonine-protein kinase PIM1 |
| NO.52 | PREP | P48147 | Prolyl endopeptidase |
| NO.52 | PSEN2 | P49810 | Presenilin-2 |
| NO.52 | PSENEN | Q9NZ42 | Gamma-secretase subunit PEN-2 |
| NO.52 | NCSTN | Q92542 | Nicastrin |
| NO.52 | APH1A | Q96BI3 | Gamma-secretase subunit APH-1A |
| NO.52 | PSEN1 | P49768 | Presenilin-1 |
| NO.52 | APH1B | Q8WW43 | Gamma-secretase subunit APH-1B |
| NO.52 | PTGS2 | P35354 | Cyclooxygenase-2 |
| NO.52 | PTPN2 | P17706 | T-cell protein-tyrosine phosphatase |
| NO.52 | RAPGEF4 | Q8WZA2 | Rap guanine nucleotide exchange factor 4 |
| NO.52 | S1PR1 | P21453 | Sphingosine 1-phosphate receptor Edg-1 |
| NO.52 | S1PR3 | Q99500 | Sphingosine 1-phosphate receptor Edg-3 |
| NO.52 | SPHK2 | Q9NRA0 | Sphingosine kinase 2 |
| NO.52 | SRD5A1 | P18405 | Steroid 5-alpha-reductase 1 |
| NO.52 | TAC3 | Q9UHF0 | Tachykinin-3 |
| NO.52 | TACR1 | P25103 | Neurokinin 1 receptor |
| NO.52 | TACR2 | P21452 | Neurokinin 2 receptor |
| NO.52 | TBXA2R | P21731 | Thromboxane A2 receptor |
| NO.52 | TGM2 | P21980 | Protein-glutamine gamma-glutamyltransferase |
| NO.52 | THRA | P10827 | Thyroid hormone receptor alpha |
| NO.52 | THRB | P10828 | Thyroid hormone receptor beta-1 |
| NO.52 | TRPM8 | Q7Z2W7 | Transient receptor potential cation channel subfamily M member 8 (by homology) |
| NO.52 | TSPO | P30536 | Translocator protein (by homology) |
| NO.52 | SQLE | Q14534 | Squalene monooxygenase |
| NO.52 | GGPS1 | O95749 | Geranylgeranyl pyrophosphate synthase |
| NO.53 | ADORA1 | P30542 | Adenosine A1 receptor |
| NO.53 | ADORA2A | P29274 | Adenosine A2a receptor |
| NO.53 | ADORA3 | P0DMS8 | Adenosine A3 receptor |
| NO.53 | CXCR3 | P49682 | C-X-C chemokine receptor type 3 |
| NO.53 | SIGMAR1 | Q99720 | Sigma opioid receptor |
| NO.53 | PGR | P06401 | Progesterone receptor |
| NO.53 | NR5A2 | O00482 | Orphan nuclear receptor LRH-1 |
| NO.53 | NR5A1 | Q13285 | Steroidogenic factor 1 |
| NO.53 | KMT5A | Q9NQR1 | N-lysine methyltransferase KMT5A |
| NO.54 | PTGS1 | [P23219](https://www.uniprot.org/uniprot/P23219) | Prostaglandin G/H synthase 1 |
| NO.54 | NCOA2 | [Q15596](https://www.uniprot.org/uniprot/Q15596) | Nuclear receptor coactivator 2 |
| NO.54 | IGHG1 | [P01857](https://www.uniprot.org/uniprot/P01857) | Ig gamma-1 chain C region |
| NO.54 | RELA | [Q04206](https://www.uniprot.org/uniprot/Q04206) | Transcription factor p65 |
| NO.54 | IL10 | [P22301](https://www.uniprot.org/uniprot/P22301) | Interleukin-10 |
| NO.54 | TNF | [P01375](https://www.uniprot.org/uniprot/P01375) | Tumor necrosis factor |
| NO.54 | IL6 | [P05231](https://www.uniprot.org/uniprot/P05231) | Interleukin-6 |
| NO.54 | PTGS2 | P35354 | Prostaglandin G/H synthase 2 |
| NO.54 | PTGER3 | [P43115](https://www.uniprot.org/uniprot/P43115) | Prostaglandin E2 receptor EP3 subtype |
| NO.54 | CA2 | P00918 | Carbonic anhydrase II |
| NO.54 | CA1 | P00915 | Carbonic anhydrase I |
| NO.54 | HSD17B3 | P37058 | Estradiol 17-beta-dehydrogenase 3 |
| NO.54 | FABP4 | P15090 | Fatty acid binding protein adipocyte |
| NO.54 | PPARA | Q07869 | Peroxisome proliferator-activated receptor alpha |
| NO.54 | FABP3 | P05413 | Fatty acid binding protein muscle |
| NO.54 | FABP5 | Q01469 | Fatty acid binding protein epidermal |
| NO.54 | PPARD | Q03181 | Peroxisome proliferator-activated receptor delta |
| NO.54 | FABP2 | P12104 | Fatty acid binding protein intestinal |
| NO.54 | CPT1A | P50416 | Carnitine O-palmitoyltransferase 1, liver isoform (by homology) |
| NO.54 | VDR | P11473 | Vitamin D receptor |
| NO.54 | SLC22A6 | Q4U2R8 | Solute carrier family 22 member 6 (by homology) |
| NO.54 | FFAR1 | O14842 | Free fatty acid receptor 1 |
| NO.54 | GPBAR1 | Q8TDU6 | G-protein coupled bile acid receptor 1 |
| NO.54 | AKR1B10 | O60218 | Aldo-keto reductase family 1 member B10 |
| NO.54 | UGT2B7 | P16662 | UDP-glucuronosyltransferase 2B7 |
| NO.54 | PTPN1 | P18031 | Protein-tyrosine phosphatase 1B |
| NO.54 | HSD11B1 | P28845 | 11-beta-hydroxysteroid dehydrogenase 1 |
| NO.54 | KDM2A | Q9Y2K7 | Lysine-specific demethylase 2A |
| NO.54 | PHF8 | Q9UPP1 | Histone lysine demethylase PHF8 |
| NO.54 | KDM5C | P41229 | Lysine-specific demethylase 5C |
| NO.54 | HMGCR | P04035 | HMG-CoA reductase |
| NO.54 | SERPINA6 | P08185 | Corticosteroid binding globulin |
| NO.54 | SHBG | P04278 | Testis-specific androgen-binding protein |
| NO.54 | G6PD | P11413 | Glucose-6-phosphate 1-dehydrogenase |
| NO.54 | GABBR1 | Q9UBS5 | GABA-B receptor (by homology) |
| NO.54 | NR1H4 | Q96RI1 | Bile acid receptor FXR |
| NO.54 | ALOX5 | P09917 | Arachidonate 5-lipoxygenase |
| NO.54 | MPEG1 | Q2M385 | Macrophage-expressed gene 1 protein |
| NO.54 | PLA2G4A | P47712 | Cytosolic phospholipase A2 |
| NO.54 | POLB | P06746 | DNA polymerase beta (by homology) |
| NO.54 | TRPV1 | Q8NER1 | Vanilloid receptor |
| NO.54 | SLC1A1 | P43005 | Excitatory amino acid transporter 3 |
| NO.54 | CNR1 | P21554 | Cannabinoid receptor 1 (by homology) |
| NO.54 | CNR2 | P34972 | Cannabinoid receptor 2 (by homology) |
| NO.54 | CYP19A1 | P11511 | Cytochrome P450 19A1 |
| NO.54 | PTGFR | P43088 | Prostanoid FP receptor |
| NO.54 | GRIN2B | Q13224 | Glutamate [NMDA] receptor subunit epsilon 2 |
| NO.54 | F2R | P25116 | Proteinase-activated receptor 1 |
| NO.54 | DCK | P27707 | Deoxycytidine kinase |
| NO.54 | ACER2 | Q5QJU3 | Alkaline ceramidase 2 |
| NO.54 | ADH1B | P00325 | Alcohol dehydrogenase 1B |
| NO.54 | ADH1A | P07327 | Alcohol dehydrogenase 1A |
| NO.54 | ADH1C | P00326 | Alcohol dehydrogenase 1C |
| NO.54 | ADH7 | P40394 | Alcohol dehydrogenase class 4 mu/sigma chain |
| NO.54 | PAM | P19021 | Peptidyl-glycine alpha-amidating monooxygenase |
| NO.54 | APEX1 | P27695 | DNA-(apurinic or apyrimidinic site) lyase |
| NO.54 | ASAH1 | Q13510 | Acid ceramidase |
| NO.54 | COL4A3BP | Q9Y5P4 | Collagen type IV alpha-3-binding protein |
| NO.54 | CA14 | Q9ULX7 | Carbonic anhydrase 14 |
| NO.54 | CA3 | P07451 | Carbonic anhydrase 3 |
| NO.54 | CA7 | P43166 | Carbonic anhydrase 7 |
| NO.54 | DAGLA | Q9Y4D2 | Sn1-specific diacylglycerol lipase alpha |
| NO.54 | POLA1 | P09884 | DNA polymerase alpha catalytic subunit |
| NO.54 | POLL | Q9UGP5 | DNA polymerase lambda |
| NO.54 | POLM | Q9NP87 | DNA-directed DNA/RNA polymerase mu |
| NO.54 | DNM1 | Q05193 | Dynamin-1 |
| NO.54 | ENPP2 | Q13822 | Ectonucleotide pyrophosphatase/phosphodiesterase family member 2 |
| NO.54 | CES1 | P23141 | Liver carboxylesterase 1 |
| NO.54 | CES2 | O00748 | Cocaine esterase |
| NO.54 | FAAH | O00519 | Fatty-acid amide hydrolase 1 |
| NO.54 | FDPS | P14324 | Farnesyl pyrophosphate synthase |
| NO.54 | FUT7 | Q11130 | Alpha-(1,3)-fucosyltransferase 7 |
| NO.54 | GBA2 | Q9HCG7 | Non-lysosomal glucosylceramidase |
| NO.54 | GGPS1 | O95749 | Geranylgeranyl pyrophosphate synthase |
| NO.54 | GNAI1 | P63096 | Guanine nucleotide-binding protein G(i) subunit alpha-1 |
| NO.54 | GNAI3 | P08754 | Guanine nucleotide-binding protein G(k) subunit alpha |
| NO.54 | GNAO1 | P09471 | Guanine nucleotide-binding protein G(o) subunit alpha |
| NO.54 | GPR174 | Q9BXC1 | Probable G-protein coupled receptor 174 |
| NO.54 | GPR34 | Q9UPC5 | Probable G-protein coupled receptor 34 |
| NO.54 | HAO1 | Q9UJM8 | Hydroxyacid oxidase 1 |
| NO.54 | EPHX1 | P07099 | Epoxide hydrolase 1 |
| NO.54 | EPHX2 | P34913 | Bifunctional epoxide hydrolase 2 |
| NO.54 | KAT2B | Q92831 | Histone acetyltransferase KAT2B |
| NO.54 | KDM5A | P29375 | Lysine-specific demethylase 5A |
| NO.54 | PRKCA | P17252 | Protein kinase C alpha type |
| NO.54 | LPAR1 | Q92633 | Lysophosphatidic acid receptor 1 |
| NO.54 | LPAR2 | Q9HBW0 | Lysophosphatidic acid receptor 2 |
| NO.54 | LPAR3 | Q9UBY5 | Lysophosphatidic acid receptor 3 |
| NO.54 | LPAR4 | Q99677 | Lysophosphatidic acid receptor 4 |
| NO.54 | LPAR5 | Q9H1C0 | Lysophosphatidic acid receptor 5 |
| NO.54 | LPAR6 | P43657 | Lysophosphatidic acid receptor 6 |
| NO.54 | SELP | P16109 | P-selectin |
| NO.54 | SLC25A20 | O43772 | Mitochondrial carnitine/acylcarnitine carrier protein |
| NO.54 | CDC25A | P30304 | M-phase inducer phosphatase 1 |
| NO.54 | CDC25B | P30305 | M-phase inducer phosphatase 2 |
| NO.54 | CDC25C | P30307 | M-phase inducer phosphatase 3 |
| NO.54 | NAAA | Q02083 | N-acylethanolamine-hydrolyzing acid amidase |
| NO.54 | NOD1 | Q9Y239 | Nucleotide-binding oligomerization domain-containing protein 1 |
| NO.54 | SMPD2 | O60906 | Sphingomyelin phosphodiesterase 2 |
| NO.54 | P2RY10 | O00398 | Putative P2Y purinoceptor 10 |
| NO.54 | PLA2G4B | P0C869 | Cytosolic phospholipase A2 beta |
| NO.54 | PLA2G4C | Q9UP65 | Cytosolic phospholipase A2 gamma |
| NO.54 | PLA2G5 | P39877 | Calcium-dependent phospholipase A2 |
| NO.54 | PLA2G2A | P14555 | Phospholipase A2, membrane associated |
| NO.54 | PLA2G2C | Q5R387 | Putative inactive group IIC secretory phospholipase A2 |
| NO.54 | PLA2G10 | O15496 | Group 10 secretory phospholipase A2 |
| NO.54 | PAOX | Q6QHF9 | Peroxisomal N(1)-acetyl-spermine/spermidine oxidase |
| NO.54 | PGA5 | P0DJD9 | Pepsin A-5 |
| NO.54 | PLCG2 | P16885 | 1-phosphatidylinositol 4,5-bisphosphate phosphodiesterase gamma-2 |
| NO.54 | POLH | Q9Y253 | DNA polymerase eta |
| NO.54 | POLK | Q9UBT6 | DNA polymerase kappa |
| NO.54 | ACP1 | P24666 | Low molecular weight phosphotyrosine protein phosphatase |
| NO.54 | PPARG | P37231 | Peroxisome proliferator-activated receptor gamma |
| NO.54 | PTPN13 | Q12923 | Tyrosine-protein phosphatase non-receptor type 13 |
| NO.54 | PTPRC | P08575 | Receptor-type tyrosine-protein phosphatase C |
| NO.54 | RARB | P10826 | Retinoic acid receptor beta |
| NO.54 | S1PR2 | O95136 | Sphingosine 1-phosphate receptor 2 |
| NO.54 | S1PR3 | Q99500 | Sphingosine 1-phosphate receptor 3 |
| NO.54 | S1PR4 | O95977 | Sphingosine 1-phosphate receptor 4 |
| NO.54 | SLC22A8 | Q8TCC7 | Solute carrier family 22 member 8 |
| NO.54 | SPHK1 | Q9NYA1 | Sphingosine kinase 1 |
| NO.54 | IARS | P41252 | Isoleucine--tRNA ligase, cytoplasmic |
| NO.54 | THRA | P10827 | Thyroid hormone receptor alpha |
| NO.54 | THRB | P10828 | Thyroid hormone receptor beta |
| NO.54 | TLR2 | O60603 | Toll-like receptor 2 |
| NO.54 | TOP2A | P11388 | DNA topoisomerase 2-alpha |
| NO.54 | VEGFA | P15692 | Vascular endothelial growth factor A |
| NO.55 | TTPA | P49638 | Alpha-tocopherol transfer protein |
| NO.55 | PTGS2 | P35354 | Prostaglandin G/H synthase 2 |
| NO.55 | UGT2B7 | P16662 | UDP-glucuronosyltransferase 2B7 |
| NO.55 | HSD11B1 | P28845 | 11-beta-hydroxysteroid dehydrogenase 1 |
| NO.55 | PTGS1 | P23219 | Cyclooxygenase-1 |
| NO.55 | NR1H3 | Q13133 | LXR-alpha |
| NO.55 | CNR2 | P34972 | Cannabinoid receptor 2 |
| NO.55 | GCGR | P47871 | Glucagon receptor |
| NO.55 | PTPN1 | P18031 | Protein-tyrosine phosphatase 1B |
| NO.55 | BACE1 | P56817 | Beta-secretase 1 |
| NO.55 | PRKCG | P05129 | Protein kinase C gamma |
| NO.55 | PRKCD | Q05655 | Protein kinase C delta |
| NO.55 | PRKCA | P17252 | Protein kinase C alpha |
| NO.55 | PRKCB | P05771 | Protein kinase C beta |
| NO.55 | PRKCE | Q02156 | Protein kinase C epsilon |
| NO.55 | PRKCH | P24723 | Protein kinase C eta |
| NO.55 | PRKCQ | Q04759 | Protein kinase C theta |
| NO.55 | CYP19A1 | P11511 | Cytochrome P450 19A1 |
| NO.55 | NPY2R | P49146 | Neuropeptide Y receptor type 2 |
| NO.55 | CHRM1 | P11229 | Muscarinic acetylcholine receptor M1 |
| NO.55 | CHRM3 | P20309 | Muscarinic acetylcholine receptor M3 |
| NO.55 | CCKBR | P32239 | Cholecystokinin B receptor (by homology) |
| NO.55 | KCNA5 | P22460 | Voltage-gated potassium channel subunit Kv1.5 |
| NO.55 | CHRM2 | P08172 | Muscarinic acetylcholine receptor M2 |
| NO.55 | TBXA2R | P21731 | Thromboxane A2 receptor |
| NO.55 | DRD2 | P14416 | Dopamine D2 receptor |
| NO.55 | SLC6A9 | P48067 | Glycine transporter 1 (by homology) |
| NO.55 | PSEN2 | P49810 | Presenilin-2 |
| NO.55 | PSENEN | Q9NZ42 | Gamma-secretase subunit PEN-2 |
| NO.55 | NCSTN | Q92542 | Nicastrin |
| NO.55 | APH1A | Q96BI3 | Gamma-secretase subunit APH-1A |
| NO.55 | PSEN1 | P49768 | Presenilin-1 |
| NO.55 | APH1B | Q8WW43 | Gamma-secretase subunit APH-1B |
| NO.55 | GRM5 | P41594 | Metabotropic glutamate receptor 5 |
| NO.55 | PER2 | O15055 | Period circadian protein homolog 2 |
| NO.55 | CHRM4 | P08173 | Muscarinic acetylcholine receptor M4 |
| NO.55 | TACR1 | P25103 | Neurokinin 1 receptor |
| NO.55 | EPHX2 | P34913 | Epoxide hydratase |
| NO.55 | OXTR | P30559 | Oxytocin receptor (by homology) |
| NO.55 | HSD11B2 | P80365 | 11-beta-hydroxysteroid dehydrogenase 2 |
| NO.55 | CHRM5 | P08912 | Muscarinic acetylcholine receptor M5 |
| NO.55 | PDE2A | O00408 | Phosphodiesterase 2A |
| NO.55 | PDE10A | Q9Y233 | Phosphodiesterase 10A |
| NO.55 | AR | P10275 | Androgen Receptor (by homology) |
| NO.55 | ESR1 | P03372 | Estrogen receptor alpha |
| NO.55 | ACHE | P22303 | Acetylcholinesterase |
| NO.55 | SLC6A2 | P23975 | Norepinephrine transporter |
| NO.55 | SLC6A4 | P31645 | Serotonin transporter |
| NO.55 | CYP2C19 | P33261 | Cytochrome P450 2C19 |
| NO.55 | CCR1 | P32246 | C-C chemokine receptor type 1 |
| NO.55 | TRPV1 | Q8NER1 | Vanilloid receptor |
| NO.55 | PABPC1 | P11940 | Polyadenylate-binding protein 1 |
| NO.55 | LYPLA1 | O75608 | Acyl-protein thioesterase 1 |
| NO.55 | CSF1R | P07333 | Macrophage colony stimulating factor receptor |
| NO.55 | LYPLA2 | O95372 | Acyl-protein thioesterase 2 |
| NO.55 | KIT | P10721 | Stem cell growth factor receptor |
| NO.55 | NR1I3 | Q14994 | Nuclear receptor subfamily 1 group I member 3 (by homology) |
| NO.55 | EPAS1 | Q99814 | Endothelial PAS domain-containing protein 1 |
| NO.55 | NR3C2 | P08235 | Mineralocorticoid receptor |
| NO.55 | PGR | P06401 | Progesterone receptor |
| NO.55 | PDE4A | P27815 | Phosphodiesterase 4A |
| NO.55 | PDE4B | Q07343 | Phosphodiesterase 4B |
| NO.55 | PDE4C | Q08493 | Phosphodiesterase 4C |
| NO.55 | MGLL | Q99685 | Monoglyceride lipase |
| NO.55 | CACNA1B | Q00975 | Voltage-gated N-type calcium channel alpha-1B subunit |
| NO.56 | PRKCA | P17252 | Protein kinase C alpha |
| NO.56 | SREBF2 | Q12772 | Sterol regulatory element-binding protein 2 |
| NO.56 | SLC2A1 | P11166 | Glucose transporter |
| NO.56 | SLC2A3 | P11169 | Solute carrier family 2, facilitated glucose transporter member 3 |
| NO.56 | SLC2A2 | P11168 | Solute carrier family 2, facilitated glucose transporter member 2 |
| NO.56 | KIT | P10721 | Stem cell growth factor receptor |
| NO.56 | FLT3 | P36888 | Tyrosine-protein kinase receptor FLT3 |
| NO.56 | PDE10A | Q9Y233 | Phosphodiesterase 10A (by homology) |
| NO.56 | MCHR1 | Q99705 | Melanin-concentrating hormone receptor 1 |
| NO.56 | SYK | P43405 | Tyrosine-protein kinase SYK |
| NO.56 | KCNK2 | O95069 | Potassium channel subfamily K member 2 (by homology) |
| NO.56 | PTPN1 | P18031 | Protein-tyrosine phosphatase 1B |
| NO.56 | MPI | P34949 | Mannose-6-phosphate isomerase |
| NO.56 | F2R | P25116 | Proteinase-activated receptor 1 |
| NO.56 | MAPK14 | Q16539 | MAP kinase p38 alpha |
| NO.56 | MMP13 | P45452 | Matrix metalloproteinase 13 |
| NO.56 | FLT1 | P17948 | Vascular endothelial growth factor receptor 1 |
| NO.56 | SCARB1 | Q8WTV0 | Scavenger receptor class B member 1 |
| NO.56 | HCRTR2 | O43614 | Orexin receptor 2 |
| NO.56 | HCRTR1 | O43613 | Orexin receptor 1 |
| NO.56 | GRM2 | Q14416 | Metabotropic glutamate receptor 2 |
| NO.56 | IDH1 | O75874 | Isocitrate dehydrogenase [NADP] cytoplasmic |
| NO.56 | JAK3 | P52333 | Tyrosine-protein kinase JAK3 |
| NO.56 | ADORA2A | P29274 | Adenosine A2a receptor (by homology) |
| NO.56 | JAK2 | O60674 | Tyrosine-protein kinase JAK2 |
| NO.56 | FAAH | O00519 | Anandamide amidohydrolase |
| NO.56 | GPR119 | Q8TDV5 | Glucose-dependent insulinotropic receptor |
| NO.56 | HLA-DRB1 | P01911 | HLA class II histocompatibility antigen, DRB1-1 beta chain |
| NO.56 | ABHD12 | Q8N2K0 | Monoacylglycerol lipase ABHD12 |
| NO.56 | ABHD16A | O95870 | Protein ABHD16A |
| NO.56 | ACER2 | Q5QJU3 | Alkaline ceramidase 2 |
| NO.56 | CHRM1 | P11229 | Muscarinic acetylcholine receptor M1 |
| NO.56 | CHRM2 | P08172 | Muscarinic acetylcholine receptor M2 |
| NO.56 | CHRM3 | P20309 | Muscarinic acetylcholine receptor M3 |
| NO.56 | CHRM4 | P08173 | Muscarinic acetylcholine receptor M4 |
| NO.56 | CHRM5 | P08912 | Muscarinic acetylcholine receptor M5 |
| NO.56 | RNPEP | Q9H4A4 | Aminopeptidase B |
| NO.56 | LAP3 | P28838 | Cytosol aminopeptidase |
| NO.56 | ANPEP | P15144 | Aminopeptidase N |
| NO.56 | ASAH1 | Q13510 | Acid ceramidase |
| NO.56 | ATF1 | P18846 | Cyclic AMP-dependent transcription factor ATF-1 |
| NO.56 | COL4A3BP | Q9Y5P4 | Collagen type IV alpha-3-binding protein |
| NO.56 | CAPN2 | P17655 | Calpain-2 catalytic subunit |
| NO.56 | CTSG | P08311 | Cathepsin G |
| NO.56 | CTSH | P09668 | Pro-cathepsin H |
| NO.56 | CTSK | P43235 | Cathepsin K |
| NO.56 | UGCG | Q16739 | Ceramide glucosyltransferase |
| NO.56 | BCHE | P06276 | Cholinesterase |
| NO.56 | CNR2 | P34972 | Cannabinoid receptor 2 |
| NO.56 | CTRB1 | P17538 | Chymotrypsinogen B |
| NO.56 | DAGLA | Q9Y4D2 | Sn1-specific diacylglycerol lipase alpha |
| NO.56 | DAGLB | Q8NCG7 | Sn1-specific diacylglycerol lipase beta |
| NO.56 | HSD17B3 | P37058 | Testosterone 17-beta-dehydrogenase 3 |
| NO.56 | DNPEP | Q9ULA0 | Aspartyl aminopeptidase |
| NO.56 | POLL | Q9UGP5 | DNA polymerase lambda |
| NO.56 | POLM | Q9NP87 | DNA-directed DNA/RNA polymerase mu |
| NO.56 | CES2 | O00748 | Cocaine esterase |
| NO.56 | FUT7 | Q11130 | Alpha-(1,3)-fucosyltransferase 7 |
| NO.56 | GBA2 | Q9HCG7 | Non-lysosomal glucosylceramidase |
| NO.56 | GABRA1 | P14867 | Gamma-aminobutyric acid receptor subunit alpha-1 |
| NO.56 | GABRA6 | Q16445 | Gamma-aminobutyric acid receptor subunit alpha-6 |
| NO.56 | GABRB1 | P18505 | Gamma-aminobutyric acid receptor subunit beta-1 |
| NO.56 | GABRB2 | P47870 | Gamma-aminobutyric acid receptor subunit beta-2 |
| NO.56 | GGPS1 | O95749 | Geranylgeranyl pyrophosphate synthase |
| NO.56 | GBA | P04062 | Glucosylceramidase |
| NO.56 | GPR174 | Q9BXC1 | Probable G-protein coupled receptor 174 |
| NO.56 | GPR34 | Q9UPC5 | Probable G-protein coupled receptor 34 |
| NO.56 | GPR88 | Q9GZN0 | Probable G-protein coupled receptor 88 |
| NO.56 | RASGRP1 | O95267 | RAS guanyl-releasing protein 1 |
| NO.56 | GSTA1 | P08263 | Glutathione S-transferase A1 |
| NO.56 | GSTM1 | P09488 | Glutathione S-transferase Mu 1 |
| NO.56 | EPHX1 | P07099 | Epoxide hydrolase 1 |
| NO.56 | KCNA5 | P22460 | Potassium voltage-gated channel subfamily A member 5 |
| NO.56 | KLK3 | P07288 | Prostate-specific antigen |
| NO.56 | LNPEP | Q9UIQ6 | Leucyl-cystinyl aminopeptidase |
| NO.56 | LTA4H | P09960 | Leukotriene A-4 hydrolase |
| NO.56 | ALOX5 | P09917 | Arachidonate 5-lipoxygenase |
| NO.56 | LPAR3 | Q9UBY5 | Lysophosphatidic acid receptor 3 |
| NO.56 | LPAR2 | Q9HBW0 | Lysophosphatidic acid receptor 2 |
| NO.56 | LCT | P09848 | Lactase-phlorizin hydrolase |
| NO.56 | LTB4R | Q15722 | Leukotriene B4 receptor 1 |
| NO.56 | MDH2 | P40926 | Malate dehydrogenase, mitochondrial |
| NO.56 | MMP1 | P03956 | Interstitial collagenase |
| NO.56 | MMP7 | P09237 | Matrilysin |
| NO.56 | MMP2 | P08253 | 72 kDa type IV collagenase |
| NO.56 | MMP3 | P08254 | Stromelysin-1 |
| NO.56 | MMP9 | P14780 | Matrix metalloproteinase-9 |
| NO.56 | CDC25A | P30304 | M-phase inducer phosphatase 1 |
| NO.56 | MTNR1A | P48039 | Melatonin receptor type 1A |
| NO.56 | NAAA | Q02083 | N-acylethanolamine-hydrolyzing acid amidase |
| NO.56 | NFKB1 | P19838 | Nuclear factor NF-kappa-B p105 subunit |
| NO.56 | NOD1 | Q9Y239 | Nucleotide-binding oligomerization domain-containing protein 1 |
| NO.56 | OXER1 | Q8TDS5 | Oxoeicosanoid receptor 1 |
| NO.56 | P2RY10 | O00398 | Putative P2Y purinoceptor 10 |
| NO.56 | P2RY12 | Q9H244 | P2Y purinoceptor 12 |
| NO.56 | PLA2G4B | P0C869 | Cytosolic phospholipase A2 beta |
| NO.56 | PLA2G2A | P14555 | Phospholipase A2, membrane associated |
| NO.56 | PLA2G2C | Q5R387 | Putative inactive group IIC secretory phospholipase A2 |
| NO.56 | PLA2G2D | Q9UNK4 | Group IID secretory phospholipase A2 |
| NO.56 | POLH | O75417 | DNA polymerase eta |
| NO.56 | POLK | Q9UBT6 | DNA polymerase kappa |
| NO.56 | PPARA | Q07869 | Peroxisome proliferator-activated receptor alpha |
| NO.56 | PPARG | P37231 | Peroxisome proliferator-activated receptor gamma |
| NO.56 | PRTN3 | P24158 | Myeloblastin |
| NO.56 | PTGES | O14684 | Prostaglandin E synthase |
| NO.56 | PTPN6 | P29350 | Tyrosine-protein phosphatase non-receptor type 6 |
| NO.56 | RARB | P10826 | Retinoic acid receptor beta |
| NO.56 | S1PR2 | O95136 | Sphingosine 1-phosphate receptor 2 |
| NO.56 | S1PR1 | P21453 | Sphingosine 1-phosphate receptor 1 |
| NO.56 | S1PR4 | O95977 | Sphingosine 1-phosphate receptor 4 |
| NO.56 | S1PR3 | Q99500 | Sphingosine 1-phosphate receptor 3 |
| NO.56 | S1PR5 | Q9H228 | Sphingosine 1-phosphate receptor 5 |
| NO.56 | SCN3A | Q9NY46 | Sodium channel protein type 3 subunit alpha |
| NO.56 | SCN8A | Q9UQD0 | Sodium channel protein type 8 subunit alpha |
| NO.56 | SPHK1 | Q9NYA1 | Sphingosine kinase 1 |
| NO.56 | STS | P08842 | Steryl-sulfatase |
| NO.56 | SI | P14410 | Sucrase-isomaltase, intestinal |
| NO.56 | TAOK3 | Q9H2K8 | Serine/threonine-protein kinase TAO3 |
| NO.56 | THRA | P10827 | Thyroid hormone receptor alpha |
| NO.56 | THRB | P10828 | Thyroid hormone receptor beta |
| NO.56 | TAS1R1 | Q7RTX1 | Taste receptor type 1 member 1 |
| NO.56 | UBLCP1 | Q8WVY7 | Ubiquitin-like domain-containing CTD phosphatase 1 |
| NO.57 | ACACB | O00763 | Acetyl-CoA carboxylase 2 |
| NO.57 | AGTR1 | P30556 | Type-1 angiotensin II receptor (by homology) |
| NO.57 | AKR1C3 | P42330 | Aldo-keto-reductase family 1 member C3 |
| NO.57 | APP | P05067 | Beta amyloid A4 protein |
| NO.57 | AVPR1A | P37288 | Vasopressin V1a receptor |
| NO.57 | BACE1 | P56817 | Beta-secretase 1 |
| NO.57 | C5AR1 | P21730 | C5a anaphylatoxin chemotactic receptor |
| NO.57 | CACNA1B | Q00975 | Voltage-gated N-type calcium channel alpha-1B subunit |
| NO.57 | CCKBR | P32239 | Cholecystokinin B receptor (by homology) |
| NO.57 | CCR1 | P32246 | C-C chemokine receptor type 1 |
| NO.57 | CDC25A | P30304 | Dual specificity phosphatase Cdc25A |
| NO.57 | CHRM1 | P11229 | Muscarinic acetylcholine receptor M1 |
| NO.57 | CHRM3 | P20309 | Muscarinic acetylcholine receptor M3 |
| NO.57 | CHRM4 | P08173 | Muscarinic acetylcholine receptor M4 |
| NO.57 | CHRM5 | P08912 | Muscarinic acetylcholine receptor M5 |
| NO.57 | CNR2 | P34972 | Cannabinoid receptor 2 |
| NO.57 | CRHR1 | P34998 | Corticotropin releasing factor receptor 1 |
| NO.57 | CSF1R | P07333 | Macrophage colony stimulating factor receptor |
| NO.57 | DRD2 | P14416 | Dopamine D2 receptor |
| NO.57 | F2R | P25116 | Proteinase-activated receptor 1 |
| NO.57 | FASN | P49327 | Fatty acid synthase |
| NO.57 | GCGR | P47871 | Glucagon receptor |
| NO.57 | GLRA1 | P23415 | Glycine receptor subunit alpha-1 |
| NO.57 | GRM2 | Q14416 | Metabotropic glutamate receptor 2 |
| NO.57 | GRM5 | P41594 | Metabotropic glutamate receptor 5 |
| NO.57 | KCNA3 | P22001 | Voltage-gated potassium channel subunit Kv1.3 |
| NO.57 | KCNA5 | P22460 | Voltage-gated potassium channel subunit Kv1.5 |
| NO.57 | KIT | P10721 | Stem cell growth factor receptor |
| NO.57 | LYPLA1 | O75608 | Acyl-protein thioesterase 1 |
| NO.57 | LYPLA2 | O95372 | Acyl-protein thioesterase 2 |
| NO.57 | MAP2K1 | Q02750 | Dual specificity mitogen-activated protein kinase kinase 1 |
| NO.57 | MDM2 | Q00987 | p53-binding protein Mdm-2 |
| NO.57 | MTNR1A | P48039 | Melatonin receptor 1A |
| NO.57 | MTNR1B | P49286 | Melatonin receptor 1B |
| NO.57 | NPY2R | P49146 | Neuropeptide Y receptor type 2 |
| NO.57 | NR1H2 | P55055 | LXR-beta |
| NO.57 | NR1H3 | Q13133 | LXR-alpha |
| NO.57 | NR3C1 | P04150 | Glucocorticoid receptor |
| NO.57 | OXTR | P30559 | Oxytocin receptor (by homology) |
| NO.57 | PABPC1 | P11940 | Polyadenylate-binding protein 1 |
| NO.57 | PDE10A | Q9Y233 | Phosphodiesterase 10A |
| NO.57 | PDE2A | O00408 | Phosphodiesterase 2A |
| NO.57 | PDE4A | P27815 | Phosphodiesterase 4A |
| NO.57 | PDE4B | Q07343 | Phosphodiesterase 4B |
| NO.57 | PDE4C | Q08493 | Phosphodiesterase 4C |
| NO.57 | PER2 | O15055 | Period circadian protein homolog 2 |
| NO.57 | PGGT1B | P53609 | Geranylgeranyl transferase type I |
| NO.57 | PGR | P06401 | Progesterone receptor |
| NO.57 | PRCP | P42785 | Lysosomal Pro-X carboxypeptidase |
| NO.57 | PRKCA | P17252 | Protein kinase C alpha |
| NO.57 | PRKCB | P05771 | Protein kinase C beta |
| NO.57 | PRKCD | Q05655 | Protein kinase C delta |
| NO.57 | PRKCE | Q02156 | Protein kinase C epsilon |
| NO.57 | PRKCG | P05129 | Protein kinase C gamma |
| NO.57 | PRKCH | P24723 | Protein kinase C eta |
| NO.57 | PRKCQ | Q04759 | Protein kinase C theta |
| NO.57 | PSEN2 | P49810 | Presenilin-2 |
| NO.57 | PSENEN | Q9NZ42 | Gamma-secretase subunit PEN-2 |
| NO.57 | NCSTN | Q92542 | Nicastrin |
| NO.57 | APH1A | Q96BI3 | Gamma-secretase subunit APH-1A |
| NO.57 | PSEN1 | P49768 | Presenilin-1 |
| NO.57 | APH1B | Q8WW43 | Gamma-secretase subunit APH-1B |
| NO.57 | PTPN1 | P18031 | Protein-tyrosine phosphatase 1B |
| NO.57 | SLC10A2 | Q12908 | Ileal bile acid transporter |
| NO.57 | SLC6A9 | P48067 | Glycine transporter 1 (by homology) |
| NO.57 | SQLE | Q14534 | Squalene monooxygenase |
| NO.57 | TACR1 | P25103 | Neurokinin 1 receptor |
| NO.57 | TRPV1 | Q8NER1 | Vanilloid receptor |
| NO.57 | GGPS1 | O95749 | Geranylgeranyl pyrophosphate synthase |
| NO.57 | LSS | P48449 | Lanosterol synthase |
| NO.57 | FNTB | P49356 | Protein farnesyltransferase subunit beta |
| NO.57 | FNTA | P49354 | Protein farnesyltransferase/geranylgeranyltransferase type-1 subunit alpha |
| NO.58 | PTGS1 | [P23219](https://www.uniprot.org/uniprot/P23219) | Prostaglandin G/H synthase 1 |
| NO.58 | PTGS2 | P35354 | Prostaglandin G/H synthase 2 |
| NO.58 | NCOA2 | [Q15596](https://www.uniprot.org/uniprot/Q15596) | Nuclear receptor coactivator 2 |
| NO.58 | RXRA | [P19793](https://www.uniprot.org/uniprot/P19793) | Retinoic acid receptor RXR-alpha |
| NO.58 | SLC6A2 | [P23975](https://www.uniprot.org/uniprot/P23975) | Sodium-dependent noradrenaline transporter |
| NO.58 | FABP4 | P15090 | Fatty acid binding protein adipocyte |
| NO.58 | FABP3 | P05413 | Fatty acid binding protein muscle |
| NO.58 | PPARG | P37231 | Peroxisome proliferator-activated receptor gamma |
| NO.58 | CNR1 | P21554 | Cannabinoid receptor 1 (by homology) |
| NO.58 | HSD11B1 | P28845 | 11-beta-hydroxysteroid dehydrogenase 1 |
| NO.58 | FAAH | O00519 | Anandamide amidohydrolase |
| NO.58 | FFAR1 | O14842 | Free fatty acid receptor 1 |
| NO.58 | PPARA | Q07869 | Peroxisome proliferator-activated receptor alpha |
| NO.58 | PTPN1 | P18031 | Protein-tyrosine phosphatase 1B |
| NO.58 | CYP19A1 | P11511 | Cytochrome P450 19A1 |
| NO.58 | CYP17A1 | P05093 | Cytochrome P450 17A1 |
| NO.58 | FABP5 | Q01469 | Fatty acid binding protein epidermal |
| NO.58 | FABP1 | P07148 | Fatty acid-binding protein, liver |
| NO.58 | CNR2 | P34972 | Cannabinoid receptor 2 |
| NO.58 | PPARD | Q03181 | Peroxisome proliferator-activated receptor delta |
| NO.58 | NR1H3 | Q13133 | LXR-alpha |
| NO.58 | CES2 | O00748 | Carboxylesterase 2 |
| NO.58 | SCD | O00767 | Acyl-CoA desaturase |
| NO.58 | PTGES | O14684 | Prostaglandin E synthase |
| NO.58 | NOS2 | P35228 | Nitric oxide synthase, inducible (by homology) |
| NO.58 | HRH3 | Q9Y5N1 | Histamine H3 receptor |
| NO.58 | HRH4 | Q9H3N8 | Histamine H4 receptor |
| NO.58 | TNKS | O95271 | Tankyrase-1 |
| NO.58 | GABRB3 | P28472 | Gamma-aminobutyric acid receptor subunit beta-3 |
| NO.58 | GABRA3 | P34903 | Gamma-aminobutyric acid receptor subunit alpha-3 |
| NO.58 | GABRG2 | P18507 | Gamma-aminobutyric acid receptor subunit gamma-2 |
| NO.58 | GABRA1 | P14867 | Gamma-aminobutyric acid receptor subunit alpha-1 |
| NO.58 | GABRA5 | P31644 | Gamma-aminobutyric acid receptor subunit alpha-5 |
| NO.58 | GABRA2 | P47869 | Gamma-aminobutyric acid receptor subunit alpha-2 |
| NO.58 | ALOX5 | P09917 | Arachidonate 5-lipoxygenase |
| NO.58 | LIMK2 | P53671 | LIM domain kinase 2 |
| NO.58 | IMPDH2 | P12268 | Inosine-5'-monophosphate dehydrogenase 2 |
| NO.58 | C5AR1 | P21730 | C5a anaphylatoxin chemotactic receptor |
| NO.58 | HTR2A | P28223 | Serotonin 2a (5-HT2a) receptor (by homology) |
| NO.58 | NAAA | Q02083 | N-acylsphingosine-amidohydrolase (by homology) |
| NO.58 | NPY5R | Q15761 | Neuropeptide Y receptor type 5 |
| NO.58 | TERT | O14746 | Telomerase reverse transcriptase |
| NO.58 | SLC9A1 | P19634 | Sodium/hydrogen exchanger 1 |
| NO.58 | HTR6 | P50406 | Serotonin 6 (5-HT6) receptor |
| NO.58 | MAOB | P27338 | Monoamine oxidase B |
| NO.58 | GRM5 | P41594 | Metabotropic glutamate receptor 5 |
| NO.58 | QPCT | Q16769 | Glutaminyl-peptide cyclotransferase |
| NO.58 | CYP11B1 | P15538 | Cytochrome P450 11B1 |
| NO.58 | CYP11B2 | P19099 | Cytochrome P450 11B2 |
| NO.58 | AR | P10275 | Androgen Receptor |
| NO.58 | ABL1 | P00519 | Tyrosine-protein kinase ABL |
| NO.58 | YES1 | P07947 | Tyrosine-protein kinase YES |
| NO.58 | LCK | P06239 | Tyrosine-protein kinase LCK |
| NO.58 | SRC | P12931 | Tyrosine-protein kinase SRC |
| NO.58 | EPHB4 | P54760 | Ephrin receptor |
| NO.58 | ALOX5AP | P20292 | 5-lipoxygenase activating protein |
| NO.58 | RARA | P10276 | Retinoic acid receptor alpha |
| NO.58 | PIM1 | P11309 | Serine/threonine-protein kinase PIM1 |
| NO.58 | SLC6A9 | P48067 | Glycine transporter 1 (by homology) |
| NO.58 | KDR | P35968 | Vascular endothelial growth factor receptor 2 |
| NO.58 | NTRK1 | P04629 | Nerve growth factor receptor Trk-A |
| NO.58 | MAPK1 | P28482 | MAP kinase ERK2 |
| NO.58 | HIPK1 | Q86Z02 | Homeodomain-interacting protein kinase 1 |
| NO.58 | HTR2C | P28335 | Serotonin 2c (5-HT2c) receptor |
| NO.58 | RORC | P51449 | Nuclear receptor ROR-gamma |
| NO.58 | PGGT1B | P53609 | Geranylgeranyl transferase type-1 subunit beta |
| NO.58 | FNTA | P49354 | Protein farnesyltransferase |
| NO.58 | GCGR | P47871 | Glucagon receptor |
| NO.58 | MAPK14 | Q16539 | MAP kinase p38 alpha |
| NO.58 | PARP2 | Q9UGN5 | Poly [ADP-ribose] polymerase 2 |
| NO.58 | AURKB | Q96GD4 | Serine/threonine-protein kinase Aurora-B |
| NO.58 | JAK2 | O60674 | Tyrosine-protein kinase JAK2 |
| NO.58 | ROCK2 | O75116 | Rho-associated protein kinase 2 |
| NO.58 | MCL1 | Q07820 | Induced myeloid leukemia cell differentiation protein Mcl-1 |
| NO.58 | KIF11 | P52732 | Kinesin-like protein 1 |
| NO.58 | BCL2 | P10415 | Apoptosis regulator Bcl-2 |
| NO.58 | TRPV1 | Q8NER1 | Vanilloid receptor |
| NO.58 | PDE5A | O76074 | Phosphodiesterase 5A |
| NO.58 | CHRNA4 | P43681 | Neuronal acetylcholine receptor subunit alpha-4 |
| NO.58 | CHRNB2 | P17787 | Neuronal acetylcholine receptor subunit beta-2 |
| NO.58 | CHRNA7 | P36544 | Neuronal acetylcholine receptor protein alpha-7 subunit |
| NO.58 | F2R | P25116 | Proteinase-activated receptor 1 |
| NO.58 | CASR | P41180 | Calcium sensing receptor |
| NO.58 | OPRD1 | P41143 | Delta opioid receptor |
| NO.58 | BRPF1 | P55201 | Peregrin |
| NO.58 | AOC3 | Q16853 | Amine oxidase, copper containing |
| NO.58 | CHUK | O15111 | Inhibitor of NF-kappa-B kinase (IKK) |
| NO.58 | PDE6D | O43924 | Phosphodiesterase 6D |
| NO.58 | NAMPT | P43490 | Nicotinamide phosphoribosyltransferase |
| NO.58 | CRHR1 | P34998 | Corticotropin releasing factor receptor 1 |
| NO.58 | CSF1R | P07333 | Macrophage colony stimulating factor receptor |
| NO.58 | KIT | P10721 | Stem cell growth factor receptor |
| NO.58 | ABHD6 | Q9BV23 | Monoacylglycerol lipase ABHD6 |
| NO.58 | SCN9A | Q15858 | Sodium channel protein type IX alpha subunit |
| NO.58 | TBXA2R | P21731 | Thromboxane A2 receptor |
| NO.58 | MTNR1A | P48039 | Melatonin receptor 1A |
| NO.58 | MTNR1B | P49286 | Melatonin receptor 1B |
| NO.58 | MTOR | P42345 | Serine/threonine-protein kinase mTOR |
| NO.58 | GSK3A | P49840 | Glycogen synthase kinase-3 alpha |
| NO.58 | LIPE | Q05469 | Hormone sensitive lipase |
| NO.58 | GRIA2 | P42262 | Glutamate receptor ionotropic, AMPA 2 |
| NO.58 | CD38 | P28907 | Lymphocyte differentiation antigen CD38 |
| NO.58 | VCP | P55072 | Transitional endoplasmic reticulum ATPase |
| NO.58 | GRIN1 | Q05586 | Glutamate receptor ionotropic, NMDA 1 |
| NO.58 | GRIN2B | Q13224 | Glutamate receptor ionotropic, NMDA 2B |
| NO.58 | CALCRL | Q16602 | Calcitonin gene-related peptide type 1 receptor |
| NO.58 | RAMP1 | O60894 | Receptor activity-modifying protein 1 |
| NO.58 | PFKFB3 | Q16875 | 6-phosphofructo-2-kinase/fructose-2,6-bisphosphatase 3 |
| NO.58 | PAM | P19021 | Peptidyl-glycine alpha-amidating monooxygenase |
| NO.58 | DAGLA | Q9Y4D2 | Sn1-specific diacylglycerol lipase alpha |
| NO.58 | POLB | P06746 | DNA polymerase beta |
| NO.58 | POLM | Q9NP87 | DNA-directed DNA/RNA polymerase mu |
| NO.58 | POLL | Q9UGP5 | DNA polymerase lambda |
| NO.58 | CES1 | P23141 | Liver carboxylesterase 1 |
| NO.58 | FFAR4 | Q5NUL3 | Free fatty acid receptor 4 |
| NO.58 | GPR174 | Q9BXC1 | Probable G-protein coupled receptor 174 |
| NO.58 | GPR34 | Q9UPC5 | Probable G-protein coupled receptor 34 |
| NO.58 | EPHX2 | P34913 | Bifunctional epoxide hydrolase 2 |
| NO.58 | KAT2B | Q92831 | Histone acetyltransferase KAT2B |
| NO.58 | KAT5 | Q92993 | Histone acetyltransferase KAT5 |
| NO.58 | PRKCA | P17252 | Protein kinase C alpha type |
| NO.58 | LPAR1 | Q92633 | Lysophosphatidic acid receptor 1 |
| NO.58 | LPAR2 | Q9HBW0 | Lysophosphatidic acid receptor 2 |
| NO.58 | LPAR3 | Q9UBY5 | Lysophosphatidic acid receptor 3 |
| NO.58 | LPAR4 | Q99677 | Lysophosphatidic acid receptor 4 |
| NO.58 | LPAR6 | P43657 | Lysophosphatidic acid receptor 6 |
| NO.58 | MGLL | Q99685 | Monoglyceride lipase |
| NO.58 | OXER1 | Q8TDS5 | Oxoeicosanoid receptor 1 |
| NO.58 | P2RY10 | O00398 | Putative P2Y purinoceptor 10 |
| NO.58 | PAFAH1B2 | P68402 | Platelet-activating factor acetylhydrolase IB subunit beta |
| NO.58 | PLA2G4C | Q9UP65 | Cytosolic phospholipase A2 gamma |
| NO.58 | POLH | Q9Y253 | DNA polymerase eta |
| NO.58 | POLK | Q9UBT6 | DNA polymerase kappa |
| NO.58 | PTPN13 | Q12923 | Tyrosine-protein phosphatase non-receptor type 13 |
| NO.58 | PTPRC | P08575 | Receptor-type tyrosine-protein phosphatase C |
| NO.58 | IARS | P41252 | Isoleucine--tRNA ligase, cytoplasmic |
| NO.59 | PTGS2 | P35354 | Prostaglandin G/H synthase 2 |
| NO.59 | RXRA | [P19793](https://www.uniprot.org/uniprot/P19793) | Retinoic acid receptor RXR-alpha |
| NO.59 | CHRM3 | [P20309](https://www.uniprot.org/uniprot/P20309) | Muscarinic acetylcholine receptor M3 |
| NO.59 | CHRM1 | [P11229](https://www.uniprot.org/uniprot/P11229) | Muscarinic acetylcholine receptor M1 |
| NO.59 | GABRA1 | [P14867](https://www.uniprot.org/uniprot/P14867) | Gamma-aminobutyric acid receptor subunit alpha-1 |
| NO.59 | PPARA | [Q07869](https://www.uniprot.org/uniprot/Q07869) | Peroxisome proliferator-activated receptor alpha |
| NO.59 | AR | P10275 | Androgen Receptor |
| NO.59 | CDC25A | P30304 | Dual specificity phosphatase Cdc25A |
| NO.59 | CDC25B | P30305 | Dual specificity phosphatase Cdc25B |
| NO.59 | CNR2 | P34972 | Cannabinoid receptor 2 |
| NO.59 | DHCR7 | Q9UBM7 | Anti-estrogen binding site (AEBS) (by homology) |
| NO.59 | EPHX2 | P34913 | Epoxide hydratase |
| NO.59 | GCGR | P47871 | Glucagon receptor |
| NO.59 | GLRA1 | P23415 | Glycine receptor subunit alpha-1 |
| NO.59 | NPC1L1 | Q9UHC9 | Niemann-Pick C1-like protein 1 |
| NO.59 | PRKCA | P17252 | Protein kinase C alpha |
| NO.59 | PRKCB | P05771 | Protein kinase C beta |
| NO.59 | PRKCD | Q05655 | Protein kinase C delta |
| NO.59 | PRKCE | Q02156 | Protein kinase C epsilon |
| NO.59 | PRKCG | P05129 | Protein kinase C gamma |
| NO.59 | PRKCH | P24723 | Protein kinase C eta |
| NO.59 | PRKCQ | Q04759 | Protein kinase C theta |
| NO.59 | RORA | P35398 | Nuclear receptor ROR-alpha |
| NO.59 | RORC | P51449 | Nuclear receptor ROR-gamma |
| NO.59 | UGT2B7 | P16662 | UDP-glucuronosyltransferase 2B7 |
| NO.59 | SQLE | Q14534 | Squalene monooxygenase |
| NO.60 | HTR2A | P28223 | 5-hydroxytryptamine receptor 2A |
| NO.60 | FAAH2 | Q6GMR7 | Fatty-acid amide hydrolase 2 |
| NO.60 | DCN | P07585 | Decorin |
| NO.60 | HTR7 | P34969 | 5-hydroxytryptamine receptor 7 |
| NO.60 | GJA1 | P17302 | Gap junction alpha-1 protein |
| NO.60 | QRSL1 | Q9H0R6 | Glutamyl-tRNA(Gln) amidotransferase subunit A, mitochondrial |
| NO.60 | ACACB | O00763 | Acetyl-CoA carboxylase 2 |
| NO.60 | ALOX5AP | P20292 | 5-lipoxygenase activating protein |
| NO.60 | CES1 | P23141 | Acyl coenzyme A: cholesterol acyltransferase |
| NO.60 | ECE1 | P42892 | Endothelin-converting enzyme 1 |
| NO.60 | HSD11B2 | P80365 | 11-beta-hydroxysteroid dehydrogenase 2 |
| NO.60 | ICAM1 | P05362 | Intercellular adhesion molecule-1 |
| NO.60 | KDR | P35968 | Vascular endothelial growth factor receptor 2 |
| NO.60 | MAPK14 | Q16539 | MAP kinase p38 alpha |
| NO.60 | PFKFB3 | Q16875 | 6-phosphofructo-2-kinase/fructose-2,6-bisphosphatase 3 |
| NO.60 | PLAU | P00749 | Urokinase-type plasminogen activator |
| NO.60 | PTAFR | P25105 | Platelet activating factor receptor |
| NO.60 | PTPN1 | P18031 | Protein-tyrosine phosphatase 1B |
| NO.60 | SELE | P16581 | Selectin E |
| NO.60 | SIRT2 | Q8IXJ6 | NAD-dependent deacetylase sirtuin 2 |
| NO.60 | SIRT3 | Q9NTG7 | NAD-dependent deacetylase sirtuin 3 |
| NO.60 | ACER2 | Q5QJU3 | Alkaline ceramidase 2 |
| NO.60 | ADH1B | P00325 | Alcohol dehydrogenase 1B |
| NO.60 | ADH7 | P40394 | Alcohol dehydrogenase class 4 mu/sigma chain |
| NO.60 | PAM | P19021 | Peptidyl-glycine alpha-amidating monooxygenase |
| NO.60 | LAP3 | P28838 | Cytosol aminopeptidase |
| NO.60 | COL4A3BP | Q9Y5P4 | Collagen type IV alpha-3-binding protein |
| NO.60 | CNR1 | P21554 | Cannabinoid receptor 1 |
| NO.60 | CNR2 | P34972 | Cannabinoid receptor 2 |
| NO.60 | CYP1A2 | P05177 | Cytochrome P450 1A2 |
| NO.60 | DAGLA | Q9Y4D2 | Sn1-specific diacylglycerol lipase alpha |
| NO.60 | HSD17B3 | P37058 | Testosterone 17-beta-dehydrogenase 3 |
| NO.60 | POLB | P06746 | DNA polymerase beta |
| NO.60 | DNM1 | Q05193 | Dynamin-1 |
| NO.60 | ENPP2 | Q13822 | Ectonucleotide pyrophosphatase/phosphodiesterase family member 2 |
| NO.60 | CES2 | O00748 | Cocaine esterase |
| NO.60 | FAAH | O00519 | Fatty-acid amide hydrolase 1 |
| NO.60 | FABP3 | P05413 | Fatty acid-binding protein, heart |
| NO.60 | FFAR4 | Q5NUL3 | Free fatty acid receptor 4 |
| NO.60 | FDPS | P14324 | Farnesyl pyrophosphate synthase |
| NO.60 | GGPS1 | O95749 | Geranylgeranyl pyrophosphate synthase |
| NO.60 | GNAI1 | P63096 | Guanine nucleotide-binding protein G(i) subunit alpha-1 |
| NO.60 | GNAI3 | P08754 | Guanine nucleotide-binding protein G(k) subunit alpha |
| NO.60 | GNAO1 | P09471 | Guanine nucleotide-binding protein G(o) subunit alpha |
| NO.60 | GPR174 | Q9BXC1 | Probable G-protein coupled receptor 174 |
| NO.60 | GPR34 | Q9UPC5 | Probable G-protein coupled receptor 34 |
| NO.60 | HAO1 | Q9UJM8 | Hydroxyacid oxidase 1 |
| NO.60 | HMGCR | P04035 | 3-hydroxy-3-methylglutaryl-coenzyme A reductase |
| NO.60 | EPHX1 | P07099 | Epoxide hydrolase 1 |
| NO.60 | EPHX2 | P34913 | Bifunctional epoxide hydrolase 2 |
| NO.60 | KDM5A | P29375 | Lysine-specific demethylase 5A |
| NO.60 | PRKCA | P17252 | Protein kinase C alpha type |
| NO.60 | PRKCE | Q02156 | Protein kinase C epsilon type |
| NO.60 | LPAR1 | Q92633 | Lysophosphatidic acid receptor 1 |
| NO.60 | LPAR2 | Q9HBW0 | Lysophosphatidic acid receptor 2 |
| NO.60 | LPAR3 | Q9UBY5 | Lysophosphatidic acid receptor 3 |
| NO.60 | LPAR4 | Q99677 | Lysophosphatidic acid receptor 4 |
| NO.60 | LPAR5 | Q9H1C0 | Lysophosphatidic acid receptor 5 |
| NO.60 | LPAR6 | P43657 | Lysophosphatidic acid receptor 6 |
| NO.60 | SELP | P16109 | P-selectin |
| NO.60 | SLC25A20 | O43772 | Mitochondrial carnitine/acylcarnitine carrier protein |
| NO.60 | MGLL | Q99685 | Monoglyceride lipase |
| NO.60 | CDC25A | P30304 | M-phase inducer phosphatase 1 |
| NO.60 | CDC25B | P30305 | M-phase inducer phosphatase 2 |
| NO.60 | CDC25C | P30307 | M-phase inducer phosphatase 3 |
| NO.60 | NOD1 | Q9Y239 | Nucleotide-binding oligomerization domain-containing protein 1 |
| NO.60 | OXER1 | Q8TDS5 | Oxoeicosanoid receptor 1 |
| NO.60 | P2RY10 | O00398 | Putative P2Y purinoceptor 10 |
| NO.60 | PAFAH1B2 | P68402 | Platelet-activating factor acetylhydrolase IB subunit beta |
| NO.60 | PLA2G4A | P47712 | Cytosolic phospholipase A2 |
| NO.60 | PLA2G4C | Q9UP65 | Cytosolic phospholipase A2 gamma |
| NO.60 | PLA2G5 | P39877 | Calcium-dependent phospholipase A2 |
| NO.60 | PLA2G2A | P14555 | Phospholipase A2, membrane associated |
| NO.60 | PLA2G2C | Q5R387 | Putative inactive group IIC secretory phospholipase A2 |
| NO.60 | PLA2G10 | O15496 | Group 10 secretory phospholipase A2 |
| NO.60 | PDCD4 | Q53EL6 | Programmed cell death protein 4 |
| NO.60 | POLH | Q9Y253 | DNA polymerase eta |
| NO.60 | POLK | Q9UBT6 | DNA polymerase kappa |
| NO.60 | ACP1 | P24666 | Low molecular weight phosphotyrosine protein phosphatase |
| NO.60 | PPARA | Q07869 | Peroxisome proliferator-activated receptor alpha |
| NO.60 | PPARG | P37231 | Peroxisome proliferator-activated receptor gamma |
| NO.60 | S1PR2 | O95136 | Sphingosine 1-phosphate receptor 2 |
| NO.60 | SLC22A6 | Q4U2R8 | Solute carrier family 22 member 6 |
| NO.60 | SLC22A8 | Q8TCC7 | Solute carrier family 22 member 8 |
| NO.60 | SLCO2A1 | Q92959 | Solute carrier organic anion transporter family member 2A1 |
| NO.60 | SOAT2 | O75908 | Sterol O-acyltransferase 2 |
| NO.60 | SPHK1 | Q9NYA1 | Sphingosine kinase 1 |
| NO.60 | SPHK2 | Q9NRA0 | Sphingosine kinase 2 |
| NO.60 | TBXA2R | P21731 | Thromboxane A2 receptor |
| NO.60 | TLR2 | O60603 | Toll-like receptor 2 |
| NO.60 | TRPV1 | Q8NER1 | Transient receptor potential cation channel subfamily V member 1 |
| NO.60 | PTGS1 | [P23219](https://www.uniprot.org/uniprot/P23219) | Prostaglandin G/H synthase 1 |
| NO.60 | NCOA2 | [Q15596](https://www.uniprot.org/uniprot/Q15596) | Nuclear receptor coactivator 2 |
| NO.60 | PTGS2 | P35354 | Prostaglandin G/H synthase 2 |
| NO.61 | PTPN1 | P18031 | Protein-tyrosine phosphatase 1B |
| NO.61 | HMGCR | P04035 | HMG-CoA reductase |
| NO.61 | ACHE | P22303 | Acetylcholinesterase |
| NO.61 | VDR | P11473 | Vitamin D receptor |
| NO.61 | CDC25A | P30304 | Dual specificity phosphatase Cdc25A |
| NO.61 | SIRT2 | Q8IXJ6 | NAD-dependent deacetylase sirtuin 2 |
| NO.61 | CA2 | P00918 | Carbonic anhydrase II |
| NO.61 | CA1 | P00915 | Carbonic anhydrase I |
| NO.61 | UGT2B7 | P16662 | UDP-glucuronosyltransferase 2B7 |
| NO.61 | CYP19A1 | P11511 | Cytochrome P450 19A1 |
| NO.61 | CA9 | Q16790 | Carbonic anhydrase IX |
| NO.61 | HSD11B1 | P28845 | 11-beta-hydroxysteroid dehydrogenase 1 |
| NO.61 | OPRK1 | P41145 | Kappa Opioid receptor |
| NO.61 | BDKRB1 | P46663 | Bradykinin B1 receptor |
| NO.61 | F2 | P00734 | Thrombin |
| NO.61 | PRKCG | P05129 | Protein kinase C gamma (by homology) |
| NO.61 | PAM | P19021 | Peptidyl-glycine alpha-amidating monooxygenase |
| NO.61 | FAAH | O00519 | Anandamide amidohydrolase |
| NO.61 | PRKCQ | Q04759 | Protein kinase C theta |
| NO.61 | JAK3 | P52333 | Tyrosine-protein kinase JAK3 |
| NO.61 | JAK2 | O60674 | Tyrosine-protein kinase JAK2 |
| NO.61 | PRKCA | P17252 | Protein kinase C alpha |
| NO.61 | EGFR | P00533 | Epidermal growth factor receptor erbB1 |
| NO.61 | AVPR2 | P30518 | Vasopressin V2 receptor |
| NO.61 | ECE1 | P42892 | Endothelin-converting enzyme 1 |
| NO.61 | CRHR1 | P34998 | Corticotropin releasing factor receptor 1 (by homology) |
| NO.61 | CDK9 | P50750 | Cyclin-dependent kinase 9 |
| NO.61 | CCNT1 | O60563 | Cyclin-T1 |
| NO.61 | HSD17B3 | P37058 | Estradiol 17-beta-dehydrogenase 3 |
| NO.61 | ABL1 | P00519 | Tyrosine-protein kinase ABL |
| NO.61 | PRCP | P42785 | Lysosomal Pro-X carboxypeptidase |
| NO.61 | PPM1B | O75688 | Protein phosphatase 2C beta |
| NO.61 | PPP1CC | P36873 | Serine/threonine protein phosphatase PP1-gamma catalytic subunit |
| NO.61 | PTGS2 | P35354 | Cyclooxygenase-2 |
| NO.61 | MET | P08581 | Hepatocyte growth factor receptor |
| NO.61 | TGFBR1 | P36897 | TGF-beta receptor type I |
| NO.61 | CACNA1B | Q00975 | Voltage-gated N-type calcium channel alpha-1B subunit |
| NO.61 | MDM2 | Q00987 | p53-binding protein Mdm-2 |
| NO.61 | AVPR1A | P37288 | Vasopressin V1a receptor (by homology) |
| NO.61 | FLT1 | P17948 | Vascular endothelial growth factor receptor 1 |
| NO.61 | GLO1 | Q04760 | Glyoxalase I |
| NO.61 | AKT1 | P31749 | Serine/threonine-protein kinase AKT |
| NO.61 | BRAF | P15056 | Serine/threonine-protein kinase B-raf |
| NO.61 | PPP5C | Q9BPW0 | Serine/threonine-protein phosphatase |
| NO.61 | MAPK10 | P53779 | c-Jun N-terminal kinase 3 |
| NO.61 | KDR | P35968 | Vascular endothelial growth factor receptor 2 |
| NO.61 | NMBR | P28336 | Neuromedin B receptor |
| NO.61 | PDE6D | O43924 | Phosphodiesterase 6D |
| NO.61 | FKBP1A | P62942 | FK506-binding protein 1A |
| NO.61 | IDH1 | O75874 | Isocitrate dehydrogenase [NADP] cytoplasmic |
| NO.61 | AURKB | Q96GD4 | Serine/threonine-protein kinase Aurora-B |
| NO.61 | PFKFB3 | Q16875 | 6-phosphofructo-2-kinase/fructose-2,6-bisphosphatase 3 |
| NO.61 | SCARB1 | Q8WTV0 | Scavenger receptor class B member 1 |
| NO.61 | KCNK3 | O14649 | Potassium channel subfamily K member 3 |
| NO.61 | KCNK9 | Q9NPC2 | Potassium channel subfamily K member 9 |
| NO.61 | MAPK14 | Q16539 | MAP kinase p38 alpha |
| NO.61 | PDE10A | Q9Y233 | Phosphodiesterase 10A |
| NO.61 | SREBF2 | Q12772 | Sterol regulatory element-binding protein 2 |
| NO.61 | ELANE | P08246 | Leukocyte elastase |
| NO.61 | ADORA3 | P0DMS8 | Adenosine A3 receptor |
| NO.61 | LCK | P06239 | Tyrosine-protein kinase LCK |
| NO.61 | PDE4B | Q07343 | Phosphodiesterase 4B |
| NO.61 | CYP2C9 | P11712 | Cytochrome P450 2C9 |
| NO.61 | MCHR1 | Q99705 | Melanin-concentrating hormone receptor 1 |
| NO.61 | F10 | P00742 | Thrombin and coagulation factor X |
| NO.61 | CFTR | P13569 | Cystic fibrosis transmembrane conductance regulator |
| NO.61 | NPY1R | P25929 | Neuropeptide Y receptor type 1 |
| NO.61 | MOGAT2 | Q3SYC2 | 2-acylglycerol O-acyltransferase 2 |
| NO.61 | GNRHR | P30968 | Gonadotropin-releasing hormone receptor |
| NO.61 | MPO | P05164 | Myeloperoxidase |
| NO.61 | DCK | P27707 | Deoxycytidine kinase |
| NO.61 | SYK | P43405 | Tyrosine-protein kinase SYK |
| NO.61 | GPR119 | Q8TDV5 | Glucose-dependent insulinotropic receptor |
| NO.61 | FYN | P06241 | Tyrosine-protein kinase FYN |
| NO.61 | PDGFRB | P09619 | Platelet-derived growth factor receptor beta |
| NO.61 | KIT | P10721 | Stem cell growth factor receptor |
| NO.61 | YES1 | P07947 | Tyrosine-protein kinase YES |
| NO.61 | CCND3 | P30281 | G1/S-specific cyclin-D3 |
| NO.61 | CCND1 | P24385 | G1/S-specific cyclin-D1 |
| NO.61 | CDK4 | P11802 | Cyclin-dependent kinase 4 |
| NO.61 | CCND2 | P30279 | G1/S-specific cyclin-D2 |
| NO.61 | PDE4A | P27815 | Phosphodiesterase 4A |
| NO.61 | NTRK1 | P04629 | Nerve growth factor receptor Trk-A |
| NO.61 | SCN9A | Q15858 | Sodium channel protein type IX alpha subunit |
| NO.61 | CCR8 | P51685 | C-C chemokine receptor type 8 |
| NO.61 | PRKCD | Q05655 | Protein kinase C delta |
| NO.61 | BRD4 | O60885 | Bromodomain-containing protein 4 |
| NO.61 | MAOB | P27338 | Monoamine oxidase B |
| NO.61 | LIMK1 | P53667 | LIM domain kinase 1 |
| NO.61 | ERBB2 | P04626 | Receptor protein-tyrosine kinase erbB-2 |
| NO.61 | CDK5R1 | Q15078 | Cyclin-dependent kinase 5 activator 1 |
| NO.61 | CDK5 | Q00535 | Cyclin-dependent-like kinase 5 |
| NO.61 | ERBB4 | Q15303 | Receptor protein-tyrosine kinase erbB-4 |
| NO.61 | BDKRB2 | P30411 | Bradykinin B2 receptor |
| NO.61 | PDE5A | O76074 | Phosphodiesterase 5A |
| NO.61 | PDE3A | Q14432 | Phosphodiesterase 3 |
| NO.61 | PDE7A | Q13946 | Phosphodiesterase 7A |
| NO.61 | SMO | Q99835 | Smoothened homolog |
| NO.61 | PPARG | P37231 | Peroxisome proliferator-activated receptor gamma |
| NO.61 | LAP3 | P28838 | Cytosol aminopeptidase |
| NO.61 | POLL | Q9UGP5 | DNA polymerase lambda |
| NO.61 | POLM | Q9NP87 | DNA-directed DNA/RNA polymerase mu |
| NO.61 | ENPP2 | Q13822 | Ectonucleotide pyrophosphatase/phosphodiesterase family member 2 |
| NO.61 | CES1 | P02795 | Liver carboxylesterase 1 |
| NO.61 | CES2 | O00748 | Cocaine esterase |
| NO.61 | FABP3 | P05413 | Fatty acid-binding protein, heart |
| NO.61 | GPR174 | Q9BXC1 | Probable G-protein coupled receptor 174 |
| NO.61 | GPR34 | Q9UPC5 | Probable G-protein coupled receptor 34 |
| NO.61 | GSTK1 | Q9Y2Q3 | Glutathione S-transferase kappa 1 |
| NO.61 | HAO1 | Q9UJM8 | Hydroxyacid oxidase 1 |
| NO.61 | LPAR1 | Q92633 | Lysophosphatidic acid receptor 1 |
| NO.61 | LPAR2 | Q9HBW0 | Lysophosphatidic acid receptor 2 |
| NO.61 | LPAR3 | Q9UBY5 | Lysophosphatidic acid receptor 3 |
| NO.61 | LPAR4 | Q99677 | Lysophosphatidic acid receptor 4 |
| NO.61 | LPAR5 | Q9H1C0 | Lysophosphatidic acid receptor 5 |
| NO.61 | LPAR6 | P43657 | Lysophosphatidic acid receptor 6 |
| NO.61 | SLC25A20 | O43772 | Mitochondrial carnitine/acylcarnitine carrier protein |
| NO.61 | NOD1 | Q9Y239 | Nucleotide-binding oligomerization domain-containing protein 1 |
| NO.61 | P2RY10 | O00398 | Putative P2Y purinoceptor 10 |
| NO.61 | PLA2G2C | Q5R387 | Putative inactive group IIC secretory phospholipase A2 |
| NO.61 | PLCG2 | P16885 | 1-phosphatidylinositol 4,5-bisphosphate phosphodiesterase gamma-2 |
| NO.61 | POLH | Q9Y253 | DNA polymerase eta |
| NO.61 | POLK | Q9UBT6 | DNA polymerase kappa |
| NO.61 | PPM1A | P35813 | Protein phosphatase 1A |
| NO.61 | SLC22A8 | Q8TCC7 | Solute carrier family 22 member 8 |
| NO.61 | TLR2 | O60603 | Toll-like receptor 2 |
| NO.61 | VEGFA | P15692 | Vascular endothelial growth factor A |
